# Uranium and thorium complexes of the phosphaethynolate ion†
†Electronic supplementary information (ESI) available: Experimental details, NMR and IR spectra, crystallographic details and files in CIF format, computational data. CCDC 1406759–1406765. For ESI and crystallographic data in CIF or other electronic format see DOI: 10.1039/c5sc02150b


**DOI:** 10.1039/c5sc02150b

**Published:** 2015-07-20

**Authors:** Clément Camp, Nicholas Settineri, Julia Lefèvre, Andrew R. Jupp, José M. Goicoechea, Laurent Maron, John Arnold

**Affiliations:** a Heavy Element Chemistry Group , Chemical Sciences Division , Lawrence Berkeley National Laboratory and Department of Chemistry , University of California , Berkeley , California 94720 , USA . Email: arnold@berkeley.edu; b LPCNO , Université de Toulouse , INSA Toulouse , 135 Avenue de Rangueil , 31077 Toulouse , France . Email: maron@irsamc.ups-tlse.fr; c Department of Chemistry , University of Oxford , Inorganic Chemistry Laboratory , South Parks Road , Oxford , OX1 3QR , UK . Email: jose.goicoechea@chem.ox.ac.uk

## Abstract

New *tris*-amidinate actinide (Th, U) complexes containing a rare O-bound terminal phosphaethynolate (OCP^**–**^) ligand were synthesized and fully characterized. The cyanate (OCN^**–**^) and thiocyanate (SCN^**–**^) analogs were prepared for comparison and feature a preferential N-coordination to the actinide metals. The Th(amid)_3_(OCP) complex reacts with Ni(COD)_2_ to yield the heterobimetallic adduct (amid)_**3**_Th(μ-η^**1**^(O):η^**2**^(C,P)-OCP)Ni(COD) featuring an unprecedented reduced (OCP^**–**^) bent fragment bridging the two metals.

## Abstract 2

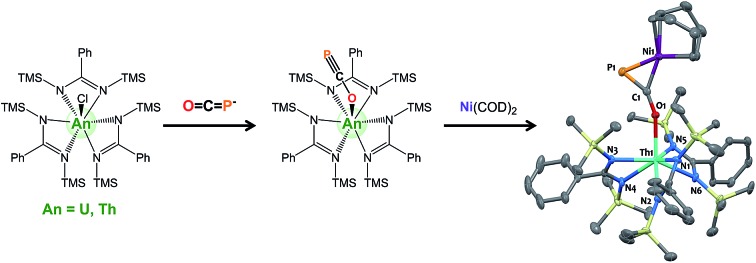
New *tris*-amidinate actinide (Th, U) complexes containing a rare O-bound terminal phosphaethynolate (OCP^–^) ligand were synthesized and fully characterized.

Efficient synthetic routes to the phosphaethynolate ion (OCP^–^) – the phosphorus analog of the cyanate anion – only appeared recently.^1^,^2^ Since then, reports by the Grützmacher and Goicoechea groups have shown that OCP^–^ exhibits a rich cycloaddition and redox chemistry which allowed the synthesis of a variety of phosphorus-containing organic derivatives.^1^,^3^–^8^ Particular interest arises from two recent studies which showed that OCP^–^ could act as a P-transfer reagent when treated with imidazolium salts^9^ or cyclotrisilene,^10^ suggesting that phosphaethynolate could be used as a convenient phosphide source. Recent computational studies also predicted the possibility to generate transition metal phosphides from M-PCO precursors through carbonyl loss.^11^ However the high reactivity of this anion can be difficult to control and generally yields diverse, unwanted decomposition products.^12^ This likely explains why thus far only a single example of a phosphaethynolate transition metal complex has been documented, Re(P=C=O)(CO)_2_(triphos) (triphos = MeC(CH_3_PPh_2_)_3_),^12^ featuring a terminal (OCP^–^) ligand which is P-bound to Re(i) and strongly bent around the pnictogen center (Fig. 1). While recent work by Grützmacher and coworkers has shown that OCP^–^ possesses an ambident nucleophilicity,^13^ several questions remain to be answered concerning its interaction with metal centers and how this compares with its cyanate and thiocyanate counterparts, as well as determining the reactivity profile of the M-bound phosphaethynolate species.

**Fig. 1:**
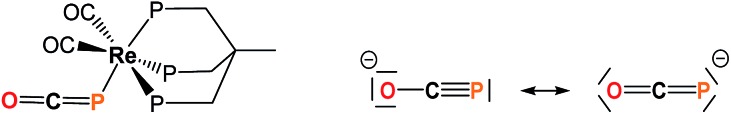
Left: structure of Re(PCO)(CO)_2_(triphos) (triphos = MeC(CH_3_PPh_2_)_3_).^14^ Right: resonance structures for the OCP^–^ anion.

We therefore targeted the use of Na(OCP)(dioxane)_*n*_^2^ as a source of the OCP^–^ ligand in order to explore its coordination properties with actinides. We reasoned that these oxophilic metals were suitable candidates to polarize the OCP^–^ moiety. Interaction of heteroallenes with actinide species has attracted substantial interest over the past few years notably due to the propensity of low-valent actinides to activate small molecules (CO_2_,^15^–^22^ CS_2_,^20^,^23^–^26^ azides^27^–^33^). Original uranium-mediated formations of cyanate involving reductive co-coupling of CO and NO^34^,^35^ and carbonylation of terminal nitrido^36^ or silylimido^37^ uranium derivatives have also been described. Here we report a series of uranium and thorium *tris*-amidinate complexes featuring linear OCP^–^, OCN^–^ and SCN^–^ ligands as well as preliminary reactivity studies involving the actinide-bound phosphaethynolate moiety with Ni(0).

Salt-metathesis reactions between the *tris*-amidinate chloride precursors MCl(amid)_3_ (**1** M = U^38^; **2** M = Th; amid = *N*,*N*′-bis(trimethylsilyl)benzamidinate) and Na(OCP)(dioxane)_2.9_ affords the desired phosphaethynolate complexes M(OCP)(amid)_3_ (**3** M = U; **4** M = Th) as block-shaped crystals in 76% and 63% isolated yields, respectively (Scheme 1). Both compounds exhibit ^1^H NMR resonance patterns in agreement with *C*_3_-symmetric solution species. The ^31^P NMR resonance for the OCP moiety in **4** (C_6_D_6_, 293 K) is significantly shifted downfield (*δ* = –334 ppm) compared to that reported for Re(P=C=O)(CO)_2_(triphos)^12^ (*δ* = –398 ppm) and alkali and alkaline earth phosphaethynolate salts (*δ*(^31^P) range: –362 to –397 ppm).^1^,^2^,^39^ Due to the paramagnetism of the U(iv) center, the ^31^P NMR signal for **3** is observed at even higher frequency (*δ* = –285 ppm). Compounds **3** and **4** feature strong IR absorption bands at almost identical wavenumbers (1685 cm^–1^ for **3**; 1683 cm^–1^ for **4**) corresponding to the C–O stretching vibrational mode of the OCP^–^ ligand. This feature appears at lower energy than that found in Re(P=C=O)(CO)_2_(triphos) (1860 cm^–1^) and alkali OCP^–^ salts (1730 to 1780 cm^–1^), which indicates weakening of the C–O bond order. As evidenced both by the low wavenumber IR absorption of *ν*_C–O_ and the downfield ^31^P NMR chemical shift, the OCP^–^ moiety in **3** and **4** is best described as a phosphaalkyne-type limiting resonance structure (see Fig. 1) in contrast with the P-bound phosphaketene-type Re(P=C=O)(CO)_2_(triphos)^12^ species. Altogether, these spectroscopic data suggest substantial strengthening of the C≡P bond upon coordination to the oxophilic actinide centers.

**Scheme 1:**
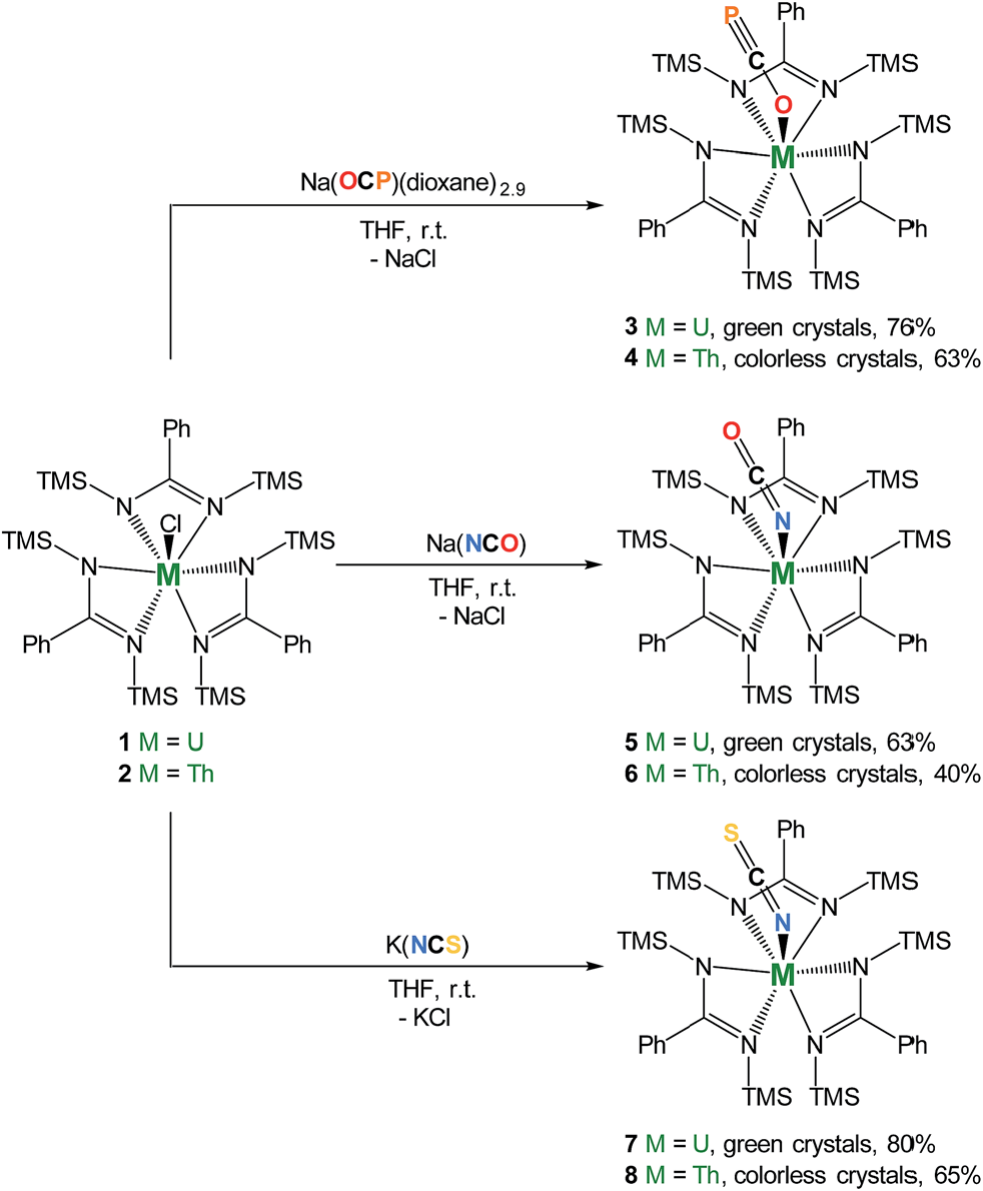
Synthesis of complexes M(OCP)(amid)_3_ (**3** M = U; **4** M = Th), M(OCN)(amid)_3_ (**5** M = U; **6** M = Th), and M(SCN)(amid)_3_ (**7** M = U; **8** M = Th). TMS = SiMe_3_.

The O-coordination of the OCP^–^ fragment is confirmed by X-ray crystallography (Fig. 2-top). Complex **4** crystallized as two independent molecules in the asymmetric unit, one of which featured an OCP^–^ group disordered over two positions with the C≡P motif pointing to two different directions in a 45 : 55 ratio. The discussion of metrical parameters for **4** is, therefore, performed on the non-disordered molecule only. The thorium and uranium analogs adopt *C*_3_-symmetry, with the three bidentate amidinate ligands wrapping around the actinide in a propeller-like geometry, and the OCP^–^ ligand pointing in the axial position. The O–C–P (179.1(4)° in **3**; 179.7(4)° in **4**) and An–O–C (170.9(3)° in **3**; 176.4(3)° in **4**) angles are close to linearity. The C≡P (1.576(5) Å in **3**; 1.561(4) Å in **4**) and C–O (1.219(6) Å in **3**; 1.246(4) Å in **4**) bond lengths are in the expected range (for comparison C≡P = 1.579(3) Å; C–O 1.212(4) Å in [K([18]crown-6)][PCO]),^1^ with a strengthening of the C≡P triple bond and a weakening of the C–O bond when compared with the metrical parameters computed for the OCP^–^ anion (C≡P = 1.625 Å; C–O 1.203 Å).^12^ The U–O (2.297(3) Å) bond length is slightly shorter than that for Th–O (2.318(2) Å), which is consistent with the larger ionic radius of Th(iv).^40^ Both distances are in the usual range and fall in between those found in actinide complexes of strongly donating aryloxide or siloxide ligands and An–O dative interactions.^41^–^46^ The U–N_amid_ and Th–N_amid_ bond distances average respectively 2.44(3) Å and 2.49(3) Å and compare well with related compounds.^38^,^47^–^49^

**Fig. 2:**
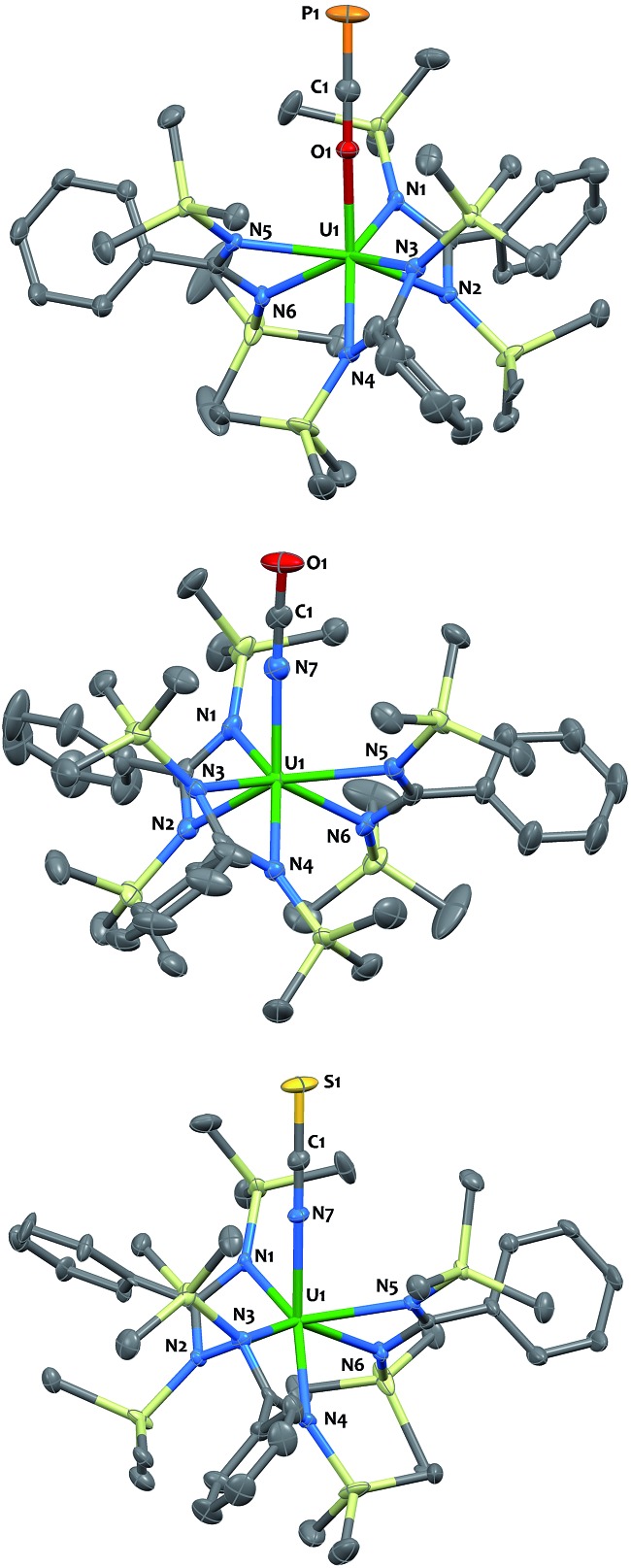
Solid-state molecular structures of compounds **3** (top), **5** (middle) and **7** (bottom) determined by single-crystal X-ray diffraction. Ellipsoids are represented with 50% probability. Metrical parameters are reported in ESI.†

The cyanate and thiocyanate counterparts **5–8** (Scheme 1) were prepared similarly, allowing a direct comparison of structurally analogous compounds. The ^1^H NMR patterns for these species are like those of compounds **1–4**, in agreement with An(iv) symmetric species in solution. The IR C–N stretches for compounds **5–8** (2199 cm^–1^ in **5**, 2200 cm^–1^ in **6**, 2021 cm^–1^ in **7** and 2018 cm^–1^ in **8**) are of high intensities and fall in the range of previously reported N-bound cyanate^36^,^37^,^50^ and thiocyanate^51^,^52^ actinide compounds. The solid-state molecular structures for compounds **5–8**, (uranium derivatives shown in Fig. 2) feature cyanate and thiocyanate moieties that are bound to the actinides in a linear fashion through the N-donor. The An–N_OCN_ (2.340(3) Å in **5**, 2.410(2) Å in **6**) and An–N_SCN_ (2.385(4) Å in **7**, 2.428 (4) Å in **8**) bond distances compare well with those reported for structurally characterized An(iv) cyanate and thiocyanate complexes.^36^,^37^,^50^–^53^ Overall, the heteroallene binding mode in these systems is similar for U(iv) and Th(iv). The most striking difference is the preferred O-coordination for OCP^–^*vs.* N-coordination in the case of OCN^–^ and SCN^–^.

Two major limiting resonance structures (Fig. 1) have to be taken into account to describe these ambiphilic heteroallene anions. Since OCN^–^ binds actinides through the N-terminus, at first glance, one could have expected OCP^–^ to behave similarly and bind through the pnictide donor, as is the case in Re(XCO)(CO)_2_(triphos) (X = P, N).^12^ Or, due to the oxophilicity of actinides, both could bind through the oxygen terminus. In fact, the difference in selectivity observed experimentally correlates with the computed partial charge in the OCP^–^ anion (*q*(O) = –0.65; *q*(P) = –0.44) and in the OCN^–^ anion (*q*(O) = –0.75; *q*(N) = –0.81);^12^ the preference is thus charge-driven.

DFT calculations are in line with the experimental observations and show that the N-bound mode is preferred with cyanate and thiocyanate anions, while the O-bound one is favored for the phosphaethynolate anion. This observation contrasts with the few previous studies which reported that the P-bound products are thermodynamically preferred;^11^–^13^ the O-bound complex **4** is 7.7 kcal mol^–1^ lower in energy than in the hypothetical P-bound analogue (Table 1). IR calculations are also in line with the experiment as the CO stretching frequency is 235 cm^–1^ lower for the O-bound complex **4** (1666 cm^–1^) than for the P-bound (1901 cm^–1^). In the case of the NCO ligand, the computed CO stretching frequency of Th–NCO (2230 cm^–1^) is 15 cm^–1^ lower than in Th–OCN (2245 cm^–1^). In the same way, for SCN, the C=N stretch is computed to be lower by 132 cm^–1^ for Th–NCS (2016 cm^–1^) over Th–SCN (2148 cm^–1^). These two sets of calculations fit with the experiment.

**Table 1:** DFT computed energy difference between the different coordination isomers in kcal mol^–1^

		Δ*E*/kcal mol^–1^
Th	Th–OCP/Th–PCO	–7.7
	Th–NCO/Th–OCN	–13.4
	Th–NCS/Th–SCN	–17.5
U	U–OCP/U–PCO	–6.2
	U–NCO/U–OCN	–15.4
	U–NCS/U–SCN	–19.4

NBO analysis of **4** indicates that the O-bound complex is preferred over the P-bound analog because of the donation from the lone pairs of the oxygen atom to the empty hybrid d/f orbital of the metal (Wiberg index of 0.42). It is worthy to note that there is no interaction between the C–P and the C–O bonds; the molecular orbitals within the OCP^–^ unit are localized onto either one or another. Contrarily, when OCP^–^ is coordinated through the lone pair of the phosphorus, a more covalent interaction is observed (Wiberg index of 0.90). Moreover, there is also a strong interaction between the lone pairs of the oxygen and the Th–P bond, giving rise to the formation of an allylic-type interaction between the three centers (P, C and O). As thorium prefers to be rather ionic, the O-bound configuration is the most energetically prominent isomer.

For the XCN^–^ anions (X = O, S), the N-bound derivatives are respectively 13.4 kcal mol^–1^ and 17.5 kcal mol^–1^ lower in energy than the O- and S-bound ones for Th (15.4 and 19.4 kcal mol^–1^ for U). The computed Δ*E* are surprisingly greater for OCN^–^ than for OCP^–^ given that the terminus charge density difference is more pronounced in OCP^–^ compared to OCN^–^ and the hard/soft mismatch is stronger for An–P bonding. This suggests that the P- *vs.* O-coordination selectivity is subtle in phosphaethynolate metal derivatives, with OCP^–^ behaving as an ambident Lewis base depending on the nature of the metal.

We have carried out preliminary reactivity investigations involving **4**. Formation of SCP^–^ has been observed upon reaction of alkali salts of the OCP^–^ anion with CS_2_; compound **4**, however, is stable with respect to [2 + 2] cycloaddition with CS_2_. Since the P-atom in **4** is protruding above the TMS groups, we reasoned that it could be accessible and act as a soft-donor to bind late transition metals. Accordingly, treatment of **4** with one equivalent of Ni(COD)_2_ results in a strong darkening of the solution and leads to the heterobimetallic adduct (amid)_3_Th(μ-η^1^(O):η^2^(C,P)-OCP)Ni(COD) **9** (Scheme 2). While **9** is the sole product resulting from the reaction of **4** with Ni(COD)_2_, NMR studies performed in benzene solution show that these species are in equilibrium. Unfortunately, these compounds exhibit similar solubility in common solvents, therefore preventing the quantitative isolation of **9** in pure form. The ^31^P NMR spectrum of **9** shows a drastic downfield shift of the signal (*δ*(^31^P) = –7.7 ppm) compared to **4** which is indicative of strong rearrangement of the phosphaethynolate moiety. Strong deshielding of the phosphorus atom is typically observed in related η^2^-phosphaalkene derivatives.^54^–^59^ The presence of an asymmetric COD environment in **9** is confirmed by ^1^H NMR which features two vinylic resonances at *δ* = 6.1 and 5.5 ppm.

**Scheme 2:**
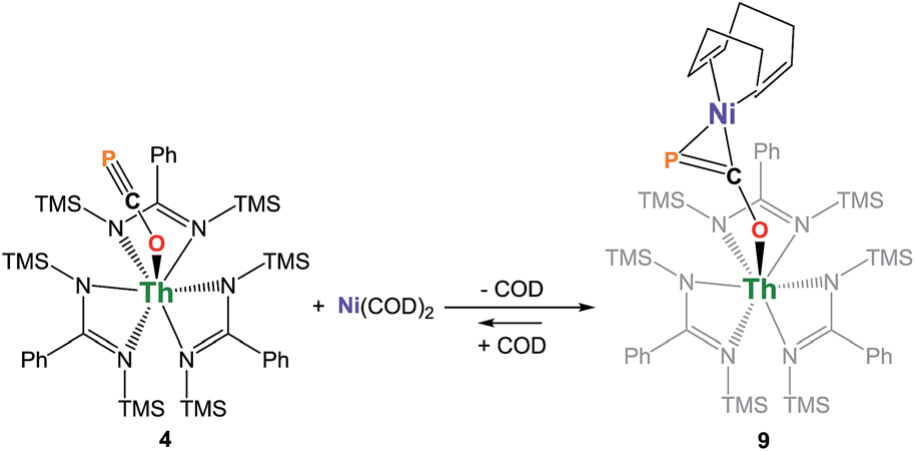
Synthesis of complex (amid)_3_Th(μ-η^1^(O):η^2^(C,P)-OCP)Ni(COD) **9**. COD = 1,5-cyclooctadiene.

Single-crystal X-ray diffraction analysis of **9** established unequivocally the formation of a three-membered nickel phosphametallacycle (C1–P1–Ni1 = 57.4(2)°) resulting from the addition of the Ni(0) center across the C≡P bond. The most striking feature of this structure (depicted in Fig. 3) is the strong bending of the OCP^–^ moiety (P–C–O angle = 148.1(3)°; Th–O–C angle = 157.5(3)°) bridging the two metals in an unprecedented μ-η^1^(O):η^2^(C,P) fashion. This is indicative of strong backbonding from the square-planar nickel center into the π* orbital of the ligated C–P unit. While these structural features are reminiscent of Ni(0) activation of phosphaalkynes^54^,^59^ and heteroallenes,^60^–^63^ the coordination mode, geometry and therefore bonding situation of the OCP^–^ moiety in **9** is unique. Electron-donation to the antibonding π* orbital results in significant elongation of the coordinated C–P bond length (1.660(4) Å) from the corresponding value of 1.561(4) Å found in **4** and falls in the range (1.630 to 1.694 Å) of side-on coordinated phosphaalkynes to nickel^54^,^59^ and other d-block metals.^55^–^58^ Both Ni1–C1 (1.895(4) Å) and Ni1–P1 (2.1705(13) Å) bond distances are within the expected range and compare well with the related phosphaalkyne complex [Ni(trop_2_NMe)(η^2^-(Ph_3_C)C≡P)]^59^ (resp. 1.887(4) and 2.2188(13) Å). The C–O bond distance (1.287(5) Å) is also elongated compared to that of **4**, while the Th–O distance (2.279(3) Å) is shortened which further indicates higher electron density on the OCP^–^ moiety.

**Fig. 3:**
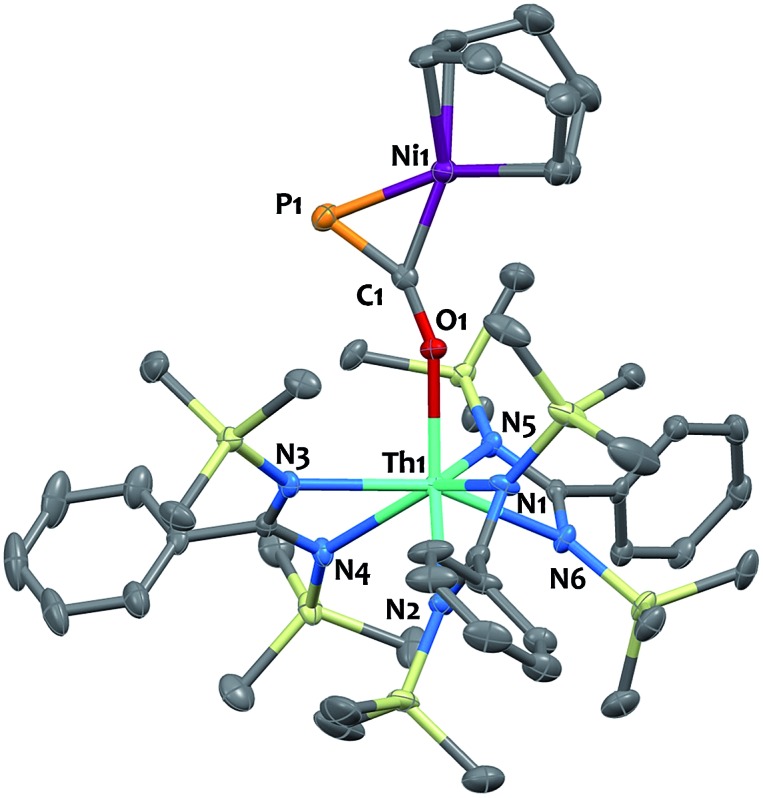
Solid-state molecular structure of compound **9** determined by single-crystal X-ray diffraction. Ellipsoids are represented with 50% probability.

Examination of the structure of **9** by DFT (see ESI† for computational details) provided an optimized structure reproducing the main experimental features. NBO analysis shows that the oxygen atom is interacting with the thorium metal center through an ionic bond involving donation from a lone pair of the oxygen to an unoccupied hybrid d/f orbital of the actinide. The phosphaethynolate π C≡P orbital overlaps with a d-orbital of the nickel to give the HOMO seen in Fig. 4.

**Fig. 4:**
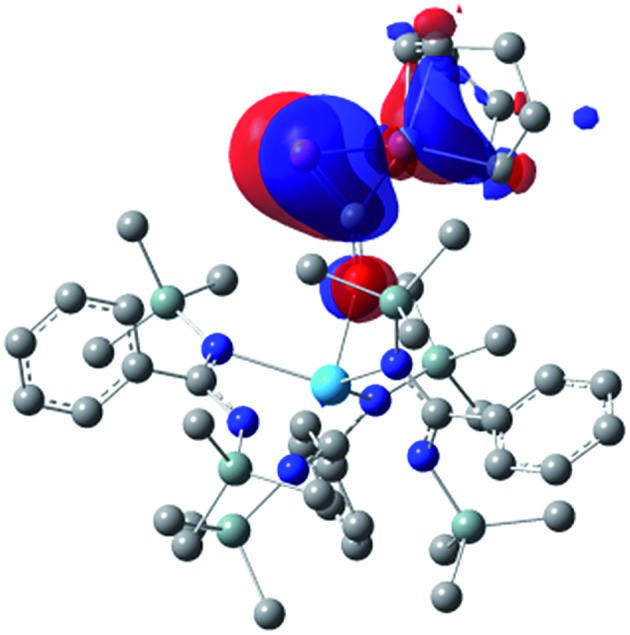
Computed HOMO orbital for complex **9**.

In summary, this study has proven the utility of Na(OCP)(dioxane)_*n*_ as a salt-metathesis reagent for accessing phosphaethynolate actinide complexes. Unlike the P-bound product favored with rhenium, O-bonding is preferred with actinides while cyanate and thiocyanate anions adopt N-bonding. Actinide coordination polarizes the OCP^–^ moiety and enhances its phosphaalkyne character. Addition of Ni(0) across the C≡P bond of the Th-bound phosphaethynolate results in the formation of an unprecedented reduced OCP^–^ moiety of bent-geometry bridging the two metals. These preliminary results pave the way towards the development of metal phosphaethynolate complexes both for reactivity purposes and to generate original heteropolymetallic architectures. Studies aiming at expanding actinide phosphaethynolate chemistry and uncovering the full range of reactivity of the metal-bound OCP^–^ moiety are ongoing in our group.

## Supplementary Material

Supplementary information

Crystal structure data

## References

[cit1] Jupp A. R., Goicoechea J. M. (2013). Angew. Chem., Int. Ed..

[cit2] Puschmann F. F., Stein D., Heift D., Hendriksen C., Gal Z. A., Grützmacher H.-F., Grützmacher H. (2011). Angew. Chem., Int. Ed..

[cit3] Heift D., Benkő Z., Grützmacher H. (2014). Angew. Chem., Int. Ed..

[cit4] Heift D., Benkő Z., Grützmacher H. (2014). Dalton Trans..

[cit5] Heift D., Benkő Z., Grützmacher H. (2014). Chem. - Eur. J..

[cit6] Chen X., Alidori S., Puschmann F. F., Santiso-Quinones G., Benkő Z., Li Z., Becker G., Grützmacher H.-F., Grützmacher H. (2014). Angew. Chem., Int. Ed..

[cit7] Jupp A. R., Goicoechea J. M. (2013). J. Am. Chem. Soc..

[cit8] Heift D., Benkő Z., Grützmacher H., Jupp A. R., Goicoechea J. M. (2015). Chem. Sci..

[cit9] Tondreau A. M., Benkő Z., Harmer J. R., Grützmacher H. (2014). Chem. Sci..

[cit10] Robinson T. P., Cowley M. J., Scheschkewitz D., Goicoechea J. M. (2015). Angew. Chem., Int. Ed..

[cit11] Lü W., Wang C., Luo Q., Li Q., Xie Y., King R. B., Schaefer III H. F. (2015). New J. Chem..

[cit12] Alidori S., Heift D., Santiso-Quinones G., Benkő Z., Grützmacher H., Caporali M., Gonsalvi L., Rossin A., Peruzzini M. (2012). Chem.–Eur. J..

[cit13] Heift D., Benkő Z., Grützmacher H. (2014). Dalton Trans..

[cit14] Alidori S., Heift D., Santiso-Quinones G., Benkő Z., Grützmacher H., Caporali M., Gonsalvi L., Rossin A., Peruzzini M. (2012). Chem.–Eur. J..

[cit15] Castro-Rodriguez I., Nakai H., Zakharov L. N., Rheingold A. L., Meyer K. (2004). Science.

[cit16] Summerscales O. T., Frey A. S. P., Cloke F. G. N., Hitchcock P. B. (2009). Chem. Commun..

[cit17] Castro-Rodriguez I., Meyer K. (2005). J. Am. Chem. Soc..

[cit18] Bart S. C., Anthon C., Heinemann F. W., Bill E., Edelstein N. M., Meyer K. (2008). J. Am. Chem. Soc..

[cit19] Tsoureas N., Castro L., Kilpatrick A. F. R., Cloke F. G. N., Maron L. (2015). Chem. Sci..

[cit20] Mougel V., Camp C., Pécaut J., Copéret C., Maron L., Kefalidis C. E., Mazzanti M. (2012). Angew. Chem., Int. Ed..

[cit21] Cooper O., Camp C., Pécaut J., Kefalidis C. E., Maron L., Gambarelli S., Mazzanti M. (2014). J. Am. Chem. Soc..

[cit22] Mansell S. M., Kaltsoyannis N., Arnold P. L. (2011). J. Am. Chem. Soc..

[cit23] Brennan J. G., Andersen R. A., Zalkin A. (1986). Inorg. Chem..

[cit24] Camp C., Cooper O., Andrez J., Pécaut J., Mazzanti M. (2015). Dalton Trans..

[cit25] Lam O. P., Heinemann F. W., Meyer K. (2011). Angew. Chem., Int. Ed..

[cit26] Lam O. P., Castro L., Kosog B., Heinemann F. W., Maron L., Meyer K. (2012). Inorg. Chem..

[cit27] Nocton G., Pécaut J., Mazzanti M. (2008). Angew. Chem., Int. Ed..

[cit28] King D. M., Tuna F., McInnes E. J. L., McMaster J., Lewis W., Blake A. J., Liddle S. T. (2012). Science.

[cit29] Camp C., Pécaut J., Mazzanti M. (2013). J. Am. Chem. Soc..

[cit30] Thomson R. K., Cantat T., Scott B. L., Morris D. E., Batista E. R., Kiplinger J. L. (2010). Nat. Chem..

[cit31] Evans W. J., Kozimor S. A., Ziller J. W. (2005). Science.

[cit32] Fox A. R., Arnold P. L., Cummins C. C. (2010). J. Am. Chem. Soc..

[cit33] Fortier S., Wu G., Hayton T. W. (2010). J. Am. Chem. Soc..

[cit34] Frey A. S. P., Cloke F. G. N., Coles M. P., Hitchcock P. B. (2010). Chem.–A Eur. J..

[cit35] Kefalidis C. E., Frey A. S. P., Roe S. M., Cloke F. G. N., Maron L. (2014). Dalton Trans..

[cit36] Cleaves P. A., King D. M., Kefalidis C. E., Maron L., Tuna F., McInnes E. J. L., McMaster J., Lewis W., Blake A. J., Liddle S. T. (2014). Angew. Chem., Int. Ed..

[cit37] Castro-Rodríguez I., Nakai H., Meyer K. (2006). Angew. Chem., Int. Ed..

[cit38] Wedler M., Knosel F., Noltemeyer M., Edelmann F. T. (1990). J. Organomet. Chem..

[cit39] Westerhausen M. (2002). J. Organomet. Chem..

[cit40] Shannon R. D. (1976). Acta Crystallogr., Sect. A: Cryst. Phys., Diffr., Theor. Gen. Crystallogr..

[cit41] Clark D. L., Grumbine S. K., Scott B. L., Watkin J. G. (1996). Organometallics.

[cit42] Lu E., Lewis W., Blake A. J., Liddle S. T. (2014). Angew. Chem., Int. Ed..

[cit43] Camp C., Andrez J., Pécaut J., Mazzanti M. (2013). Inorg. Chem..

[cit44] Camp C., Kefalidis C. E., Pécaut J., Maron L., Mazzanti M. (2013). Angew. Chem., Int. Ed..

[cit45] Pool J. A., Scott B. L., Kiplinger J. L. (2005). J. Am. Chem. Soc..

[cit46] Camp C., Mougel V., Pécaut J., Maron L., Mazzanti M. (2013). Chem.–A Eur. J..

[cit47] Karmel I. S. R., Elkin T., Fridman N., Eisen M. S. (2014). Dalton Trans..

[cit48] Villiers C., Thuéry P., Ephritikhine M. (2004). Eur. J. Inorg. Chem..

[cit49] Camp C., Antunes M. A., Garcia G., Ciofini I., Santos I. C., Pecaut J., Almeida M., Marcalo J., Mazzanti M. (2014). Chem. Sci..

[cit50] Thomson R. K., Scott B. L., Morris D. E., Kiplinger J. L. (2010). C. R. Chim..

[cit51] Al-Daher A. G. M., Bagnall K. W., Castellani C. B., Benetollo F., Bombieri G. (1984). Inorg. Chim. Acta.

[cit52] Fischer R. D., Klähne E., Kopf J. (1978). Z. Naturforsch., B: Anorg. Chem., Org. Chem..

[cit53] Bagnall K. W., Benetollo F., Forsellini E., Bombieri G. (1992). Polyhedron.

[cit54] Schaub T., Radius U. (2006). Z. Anorg. Allg. Chem..

[cit55] Laurent J. C. T. R. B.-S., Hitchcock P. B., Kroto H. W., Nixon J. F. (1981). J. Chem. Soc., Chem. Commun..

[cit56] Binger P., Biedenbach B., Herrmann A. T., Langhauser F., Betz P., Goddard R., Krüger C. (1990). Chem. Ber..

[cit57] Mansell S. M., Green M., Russell C. A. (2012). Dalton Trans..

[cit58] Burrows A. D., Dransfeld A., Green M., Jeffery J. C., Jones C., Lynam J. M., Nguyen M. T. (2001). Angew. Chem., Int. Ed..

[cit59] Trincado M., Rosenthal A. J., Vogt M., Grützmacher H. (2014). Eur. J. Inorg. Chem..

[cit60] Aresta M., Nobile C. F., Albano V. G., Forni E., Manassero M. (1975). J. Chem. Soc., Chem. Commun..

[cit61] Anderson J. S., Iluc V. M., Hillhouse G. L. (2010). Inorg. Chem..

[cit62] Mindiola D. J., Hillhouse G. L. (2002). Chem. Commun..

[cit63] Iluc V. M., Hillhouse G. L. (2014). J. Am. Chem. Soc..

