# Supplementary material for: Uranium and thorium complexes of the phosphaethynolate ion
Source: Chem Sci. 2015 Jul 20;6(11):6379–84. doi: 10.1039/c5sc02150b (PMC6054101; doi:10.1039/c5sc02150b)
Supplement: Supplementary file 1 [file SC-006-C5SC02150B-s001.pdf]

Electronic Supplementary Material (ESI) for Chemical Science.
This journal is © The Royal Society of Chemistry 2015

Supporting Information

Uranium and Thorium Complexes of the Phosphaethynolate Ion

Clément Camp,[a] Nicholas Settineri,[a] Julia Lefèvre,[b] Andrew R. Jupp,[c] Jose M. Goicoechea,[c] Laurent Maron[,b] and John Arnold*[,a]*

[a] Heavy Element Chemistry Group, Chemical Sciences Division, Lawrence Berkeley National Laboratory and Department of Chemistry, University of California, Berkeley, California 94720, United States. arnold@berkeley.edu

[b] LPCNO, Université de Toulouse, INSA Toulouse, 135 Avenue de Rangueil, 31077 Toulouse, France. maron@irsamc.ups-tlse.fr

[c] Department of Chemistry, University of Oxford, Inorganic Chemistry Laboratory, South Parks Road, Oxford, OX1 3QR, UK. jose.goicoechea@chem.ox.ac.uk

Content

A. Experimental section 2

B. NMR and IR spectroscopic data 5

C. X-ray crystallography 14

D. DFT calculations 18

E. References 67

A. Experimental section

General Considerations. Unless otherwise noted, all reactions were performed either using standard Schlenk line techniques or in an MBraun inert atmosphere glovebox under an atmosphere of purified nitrogen (<1 ppm O_2/H_2O). Glassware and cannulae were stored in an oven at ~160 °C for at least 12 h prior to use. Hexanes, THF and toluene were purified by passage through a column of activated alumina, stored over 3 or 4 Å molecular sieves, and degassed prior to use. C_6D_6 was dried over sodium/benzophenone and then vacuum-transferred to a storage flask and freeze-pump-thaw degassed before being stored over activated molecular sieves in the drybox. UCl_4,[1] $ThCl_4(DME)_2$,[2] (amid)Li(TMEDA)[3] and Na(OCP)$(dioxane)_{2.9}$[4] were prepared using literature procedures. All other reagents were acquired from commercial sources and used as received. NMR spectra were recorded on Bruker AVQ-400, AVB-400, DRX-500, AV-500, and AV-600 spectrometers. Chemical shifts were measured relative to residual solvent peaks, which were assigned relative to an external TMS standard set at 0.00 ppm. ^{31}P chemical shifts were referenced to an external standard (Ph_3PO for ^{31}P set at 23 ppm). ^{1}H and ^{13}C NMR assignments were routinely confirmed by $^{1}H-^{1}H$ COSY and $^{1}H-^{13}C$ HSQC experiments. Infrared (IR) spectra were recorded with a Thermo Scientific Nicolet iS10 series FTIR spectrophotometer as a Nujol mull between KBr plates. The uncorrected melting points were determined using sealed capillaries prepared under nitrogen on an Optmelt SRS. Elemental analyses were performed at the School of Human Sciences, Science Center, London Metropolitan University. The X-ray structural determinations were performed at CHEXRAY, University of California, Berkeley on a Bruker SMART APEX II QUAZAR diffractometer. Solution magnetic moments were obtained by Evan's Method. Details concerning X-ray diffraction analyses and DFT computational studies are provided in the following sections.

Synthetic methods

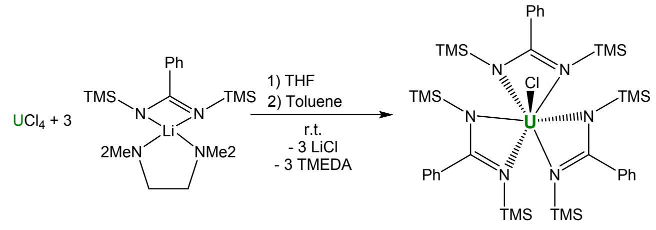

1 green crystals, 88%

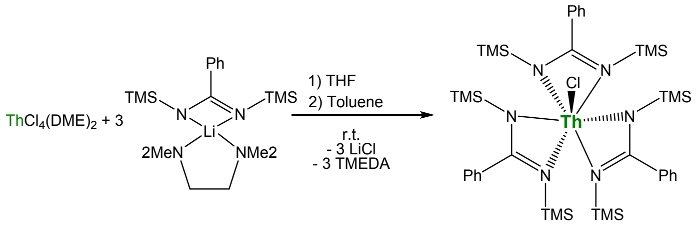

2 colorless crystals, 59%

UCl$(amid)_3$ (1)

This compound was prepared according to a modified published procedure.[5] A 50 mL THF solution of (amid)Li(TMEDA) (3.15 g, 8.13 mmol, 3 equiv.) was added to UCl_4 (1.03 g, 2.71 mmol, 1 equiv.) and the reaction mixture was vigorously stirred at room temperature for 15 h affording a green suspension. The THF solvent was then removed *in vacuo* and the residue was triturated with 2x10 mL toluene and taken to dryness. The green residue was then extracted with 3x30mL toluene and filtered. The resulting green filtrate was concentrated to 30 mL and allowed to crystallize at -40°C in a freezer for 24 h. This produced large bright green crystals that were recovered and dried *in vacuo* (2.55 g, 88 % yield). The crystal structure for **1** was previously reported by Edelmann and coworkers.[5] ^{1}H NMR (400 MHz, C_6D_6, 293 K) δ = 15.3 (br s, 3H, CH_{Ar}), 10.9 (br s, 3H, CH_{Ar}), 9.3 (br s, 6H, CH_{Ar}), 8.8 (t, 3H, CH_{Ar}), 1.3 (s, 27H, $SiMe_3$), -3.1 (s, 27H, $SiMe_3$). FT-IR (cm^{-1}) 2915 (s), 2851 (s), 1463 (s), 1386 (s), 1245 (s), 1157 (w), 1075 (w), 1000 (w), 973 (s), 920 (w), 841 (s), 784 (s), 759 (s), 704 (s), 477(s). M.p. 317(2)°C, decomp. Anal. calcd for $C_{39}H_{69Cl}N_6Si_6U$: C, 44.02; H, 6.54; N, 7.90. Found: C, 43.98; H, 6.47; N, 7.93.

ThCl(amid)$_3$ (2)

This compound was prepared in an analogous fashion to that previously stated for **1**. (amid)Li(TMEDA) (5.01 g, 12.9 mmol, 3 equiv.) and $ThCl_4(DME)_2$ (2.39 g, 4.3 mmol, 1 equiv.) yielded 2.71 g of **2** (59 % yield). Colorless crystals suitable for X-ray diffraction were grown from a concentrated toluene solution kept at -40 °C for 24 h. ^{1}H NMR (500 MHz, C_6D_6, 293 K) δ = 7.5 (br s, 6H, CH_{Ar}), 7.0 (m, 9H, CH_{Ar}), 0.4 (s, 27H, $SiMe_3$), 0.2 (s, 27H, $SiMe_3$). FT-IR (cm^{-1}) 2922 (s), 2853 (s), 1463 (s), 1246 (s), 1160 (w), 1025 (w), 1000 (w), 978 (s), 920 (w), 842 (s), 785 (m), 761 (s), 717 (s), 479 (s). M.p. 317(2)°C, decomp. Anal. calcd for $C_{39}H_{69}N_6Si_6ClTh$: C, 44.15; H, 6.84; N, 7.92. Found: C, 44.16; H, 6.71; N, 7.85.

U(OCP)(amid)$_3$ (3)

A 10 mL THF solution of UCl(amid)$_3$ (300 mg, 0.282 mmol, 1 equiv.) and Na(OCP)(dioxane)$_{2.9}$ (100 mg, 0.296 mmol, 1.05 equiv.) was stirred at room temperature for 24 h. The THF solvent was then removed *in vacuo* and the residue was extracted with 2 mL toluene and filtered. The green filtrate was allowed to crystallize at -40°C in a freezer for 48 h. This produced green block-shaped crystals that were recovered and dried *in vacuo* (234 mg, 76 % yield). Single crystals suitable for X-ray diffraction were obtained similarly. ^{1}H NMR (400 MHz, C_6D_6, 293 K) δ = 13.7 (br s, 3H, CH_{Ar}), 10.3 (br s, 3H, CH_{Ar}), 8.9 (t, 6H, CH_{Ar}), 8.4 (t, 3H, CH_{Ar}), 0.5 (s, 27H, $SiMe_3$), -2.6 (s, 27H, $SiMe_3$). ^{31}P NMR (243 MHz, C_6D_6, 293 K) δ = -285.2 (s, OC*P*). ^{13}C NMR (125.8 MHz, C_6D_6, 293 K) δ = 142.7 (C_{Ar}), 132.9 (C_{Ar}), 4.6 (SiMe_3), 1.8 (SiMe_3). FT-IR (cm^{-1}) 2955 (s), 2925 (s), 2852 (s), 1685 (s, PCO), 1463 (s), 1386 (s), 1247 (s), 1179 (w), 1157 (w), 1000 (w), 974 (s), 931 (w), 920 (w), 838 (s), 783 (s), 761 (s), 704 (s). M.p. 233(2)°C, decomp. μ_{eff} = 3.23 μ_B (C_6D_6, 293 K, Evans'method). Anal. calcd for $C_{40}H_{69}N_6OPSi_6U$: C, 44.18; H, 6.40; N, 7.73. Found: C, 43.83; H, 6.31; N, 7.62.

Th(OCP)(amid)$_3$ (4)

This compound was prepared in an analogous fashion to that previously stated for **3**. ThCl(amid)$_3$ (298 mg, 0.282 mmol, 1 equiv.) and Na(OCP)(dioxane)$_{2.9}$ (100 mg, 0.296 mmol, 1.05 equiv.) yielded 191 mg of **4** (63%). Colorless crystals suitable for X-ray diffraction were grown from a concentrated toluene solution kept at -40 °C for 24 h. ^{1}H NMR (400 MHz, C_6D_6, 293 K) δ = 7.3 (br s, 6H, CH_{Ar}), 7.0 (m, 9H, CH_{Ar}), 0.3 (s, 27H, $SiMe_3$), 0.1 (s, 27H, $SiMe_3$). ^{31}P NMR (243 MHz, C_6D_6, 293 K) δ = -334.4 (s, OC*P*). ^{13}C NMR (125.8 MHz, C_6D_6, 293 K) δ = 181.7 (N*C*N), 143.0 (C_{Ar}), 128.8 (C_{Ar}), 126.8 (C_{Ar}), 3.4 (SiMe_3), 2.7 (SiMe_3). FT-IR (cm^{-1}) 2923 (s), 2853 (s), 1683 (s, PCO), 1385 (s), 1248 (s), 978 (s), 839 (s), 785 (s), 761 (s), 703 (s). M.p. 277(2)°C, decomp. Anal. calcd for $C_{40}H_{69}N_6OPSi_6Th$: C, 44.42; H, 6.43; N, 7.77. Found: C, 44.35; H, 6.35; N, 7.69.

U(NCO)(amid)$_3$ (5)

A 5 mL THF suspension of UCl(amid)$_3$ (195 mg, 0.183 mmol, 1 equiv.) and sodium cyanate (13.7 mg, 0.211 mmol, 1.15 equiv.) was stirred at room temperature for 24 h. The THF solvent was then removed *in vacuo* and the residue was extracted with 1.5 mL toluene and filtered. The green filtrate was allowed to crystallize at -40°C in a freezer for 48 h. This produced green block-shaped crystals that were recovered and dried *in vacuo* (123 mg, 63 % yield). Single crystals suitable for X-ray diffraction were obtained similarly. ^{1}H NMR (400 MHz, C_6D_6, 293 K) δ = 13.2 (br s, 3H, CH_{Ar}), 10.1 (br s, 3H, CH_{Ar}), 8.8 (t, 6H, CH_{Ar}), 8.4 (t, 3H, CH_{Ar}), 0.5 (s, 27H, $SiMe_3$), -2.5 (s, 27H, $SiMe_3$). ^{13}C NMR (125.8 MHz, C_6D_6, 293 K) δ = 144.0 (C_{Ar}), 132.7 (C_{Ar}), 3.4 (SiMe_3), 1.9 (SiMe_3). FT-IR (cm^{-1}) 3063 (w), 2922 (s), 2853 (s), 2199 (s, OCN), 1398 (s), 1246 (s), 1157 (w), 1074 (w), 1000 (w), 973 (s), 920 (w), 847 (s), 784 (s), 758 (s), 706 (s). M.p. 288(2)°C, decomp. Anal. calcd for $C_{40}H_{69}N_7OSi_6U$: C, 44.88; H, 6.50; N, 9.16. Found: C, 44.72; H, 6.61; N, 9.06.

Th(NCO)(amid)$_3$ (6)

This compound was prepared in an analogous fashion to that previously stated for **5**, although a longer reaction time of 96 h and an excess of sodium cyanate were required. ThCl(amid)$_3$ (297 mg, 0.281 mmol, 1 equiv.) and sodium cyanate (40 mg, 0.615 mmol, 2.19 equiv.) yielded 120 mg of **6** (40%). Colorless crystals suitable for X-ray diffraction were grown from a concentrated toluene solution kept at -40 °C for 24 h. ^{1}H NMR (400 MHz, C_6D_6, 293 K) δ = 7.4 (br s, 6H, CH_{Ar}), 7.0 (m, 9H, CH_{Ar}), 0.3 (s, 27H, $SiMe_3$), 0.2 (s, 27H, $SiMe_3$). ^{13}C NMR (125.8 MHz, C_6D_6, 293 K) δ = 181.7 (N*C*N), 143.0 (C_{Ar}), 128.8 (C_{Ar}), 126.6 (C_{Ar}), 3.2 (SiMe_3), 2.3

(Si*Me*$_3$). FT-IR (cm^{-1}) 2923 (s), 2853 (s), 2200 (s), 1663 (w), 1463 (s), 1378 (s), 1246 (s), 1157 (w), 1074 (w), 1000 (w), 978 (s), 842 (s), 785 (w), 758 (m), 716 (m), 703 (m), 630 (w), 479 (s). M.p. 205(2)°C, decomp. Anal. calcd for $C_{40}H_{69}N_7OSi_6Th$: C, 45.15; H, 6.53; N, 9.21. Found: C, 44.93; H, 6.72; N, 9.15.

U(NCS)(amid)$_3$ (7)

A 5 mL THF suspension of UCl(amid)$_3$ (163 mg, 0.153 mmol, 1 equiv.) and potassium thiocyanate (17.1 mg, 0.176 mmol, 1.15 equiv.) was stirred at room temperature for 24 h. The THF solvent was then removed *in vacuo* and the residue was extracted with 1.5 mL toluene and filtered. The green filtrate was allowed to crystallize at -40°C in a freezer for 48 h. This produced green block-shaped crystals that were recovered and dried *in vacuo* (133 mg, 80 % yield). Single crystals suitable for X-ray diffraction were obtained similarly. ^{1}H NMR (500 MHz, C_6D_6, 293 K) δ = 15.6 (br s, 3H, CH_{Ar}), 11.1 (br s, 3H, CH_{Ar}), 9.5 (br s, 6H, CH_{Ar}), 8.9 (t, 3H, CH_{Ar}), 0.9 (s, 27H, SiMe$_3$), -3.4 (s, 27H, SiMe$_3$). ^{13}C NMR (125.8 MHz, C_6D_6, 293 K) δ = 151.1 (C_{Ar}), 133.7 (C_{Ar}), 4.6 (Si*Me*$_3$), 0.8 (Si*Me*$_3$). FT-IR (cm^{-1}) 3062 (w), 3024 (w), 2924 (s), 2854 (s), 2021 (s, SCN), 1386 (s), 1247 (s), 1179 (w), 1158 (w), 1000 (w), 973 (s), 922 (w), 834 (s), 783 (s), 761 (s), 704 (s). M.p. 288(2)°C, decomp. Anal. calcd for $C_{40}H_{69}N_7S_1Si_6U$: C, 44.21; H, 6.40; N, 9.02. Found: C, 44.19; H, 6.50; N, 8.98.

Th(NCS)(amid)$_3$ (8)

This compound was prepared in an analogous fashion to that previously stated for **7**. ThCl(amid)$_3$ (303 mg, 0.286 mmol, 1 equiv.) and potassium thiocyanate (34 mg, 0.350 mmol, 1.22 equiv.) yielded 201 mg of **8** (65%). Colorless crystals suitable for X-ray diffraction were grown from a concentrated toluene solution kept at -40 °C for 24 h. ^{1}H NMR (400 MHz, C_6D_6, 293 K) δ = 7.4 (br s, 6H, CH_{Ar}), 7.0 (m, 9H, CH_{Ar}), 0.3 (s, 27H, SiMe$_3$), 0.1 (s, 27H, SiMe$_3$). ^{13}C NMR (125.8 MHz, C_6D_6, 293 K) δ = 182.1 (N*C*N), 142.7 (C_{Ar}), 129.0 (C_{Ar}), 126.6 (C_{Ar}), 3.1 (Si*Me*$_3$), 2.3 (Si*Me*$_3$). FT-IR (cm^{-1}) 2921 (s), 2854 (s), 2018 (s), 1444 (s), 1382 (s), 1246 (s), 1179 (w), 1158 (w), 1001 (w), 978 (s), 921 (w), 846 (s), 784 (w), 760 (m), 702 (s), 604 (w), 479 (s). M.p. 308(2)°C, decomp. Anal. Calcd for $C_{40}H_{69}N_7S_1Si_6Th$: C, 44.46; H, 6.44; N, 9.07. Found: C, 44.51; H, 6.51; N, 8.89.

(amid)$_3$Th(μ-η^1(O):η^2(C,P)-OCP)Ni(COD) (9)

A 2 mL toluene solution of Th(OCP)(amid)$_3$ **4** (255 mg, 0.236 mmol, 1 equiv.) and *bis*(1,5-cyclooctadiene)nickel(0) (64.9 mg, 0.236 mmol, 1 equiv.) was stirred at room temperature for 12 h during which time the solution turned from pale yellow to dark brown within a few hours. Monitoring the reaction by ^{1}H and ^{31}P NMR showed that the reaction reaches equilibrium after approximatively 12h at room temperature. The solvent was then removed *in vacuo* and the residue was extracted with 0.5 mL hexane and filtered. The dark brown filtrate was allowed to crystallize at -40°C in a freezer for 72 h. This produced a dark brown crystalline material (122 mg) containing **9** as well as **4** and Ni(COD)$_2$. Despite several attempts, the purification of **9** from its starting materials by fractional recrystallization proved highly challenging since these species exhibit similar high-solubility in common solvents and limited stability. In one occurrence, few crystals of **9** (1% yield) were obtained upon 4 successive recrystallizations of the reaction materials in hexanes at -40°C. These were sufficient to get X-ray diffraction and NMR spectroscopy data. The latter revealed that **9** exhibits limited stability in C_6D_6 solution (Figure S17 and S18) and is converted back to **4** through the loss of free COD, Ni(COD)$_2$ and other non-identified Ni species. ^{1}H NMR (600 MHz, C_6D_6, 293 K) δ = 7.5 (3, 6H, CH_{Ar}), 7.0 (m, 12H, CH_{Ar}), 6.1 (m, 2H, CH=CH$_{COD}$), 5.5 (m, 2H, CH=CH_{COD}), 2.4 (m, 2H, CH_{2COD}), 2.2 (m, 2H, CH_{2COD}), 2.0 (m, 2H, CH_{2COD}), 1.9 (m, 2H, CH_{2COD}), 0.3 (s, 27H, SiMe$_3$). ^{31}P NMR (243 MHz, C_6D_6, 293 K) δ = -7.7 (s, OC*P*).

B. NMR and IR spectroscopic data

Figure S1. ^{1}H NMR spectrum for $UClL_3$ **1** (C_6D_6, 293K, 400MHz).

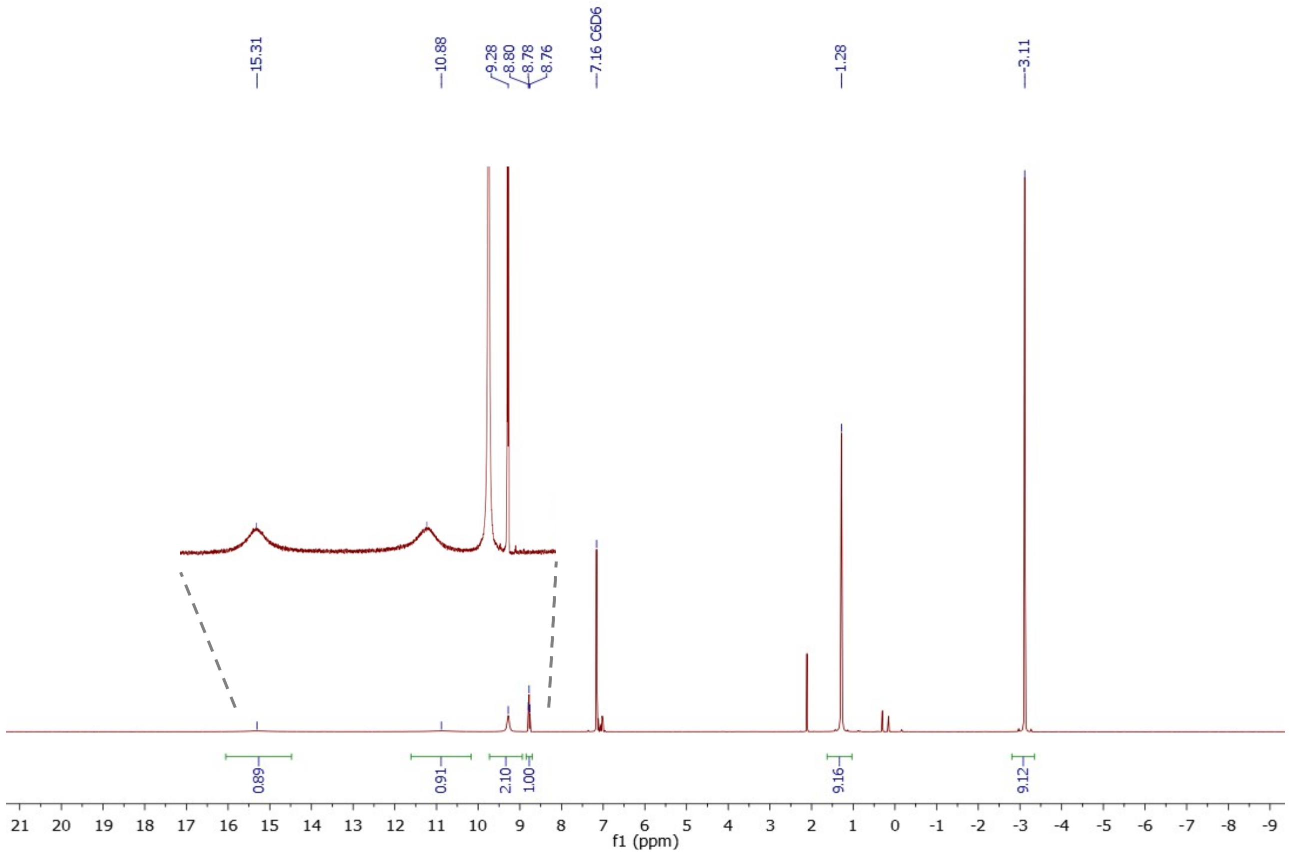

Figure S2. ^{1}H NMR spectrum for $ThClL_3$ **2** (C_6D_6, 293K, 400MHz).

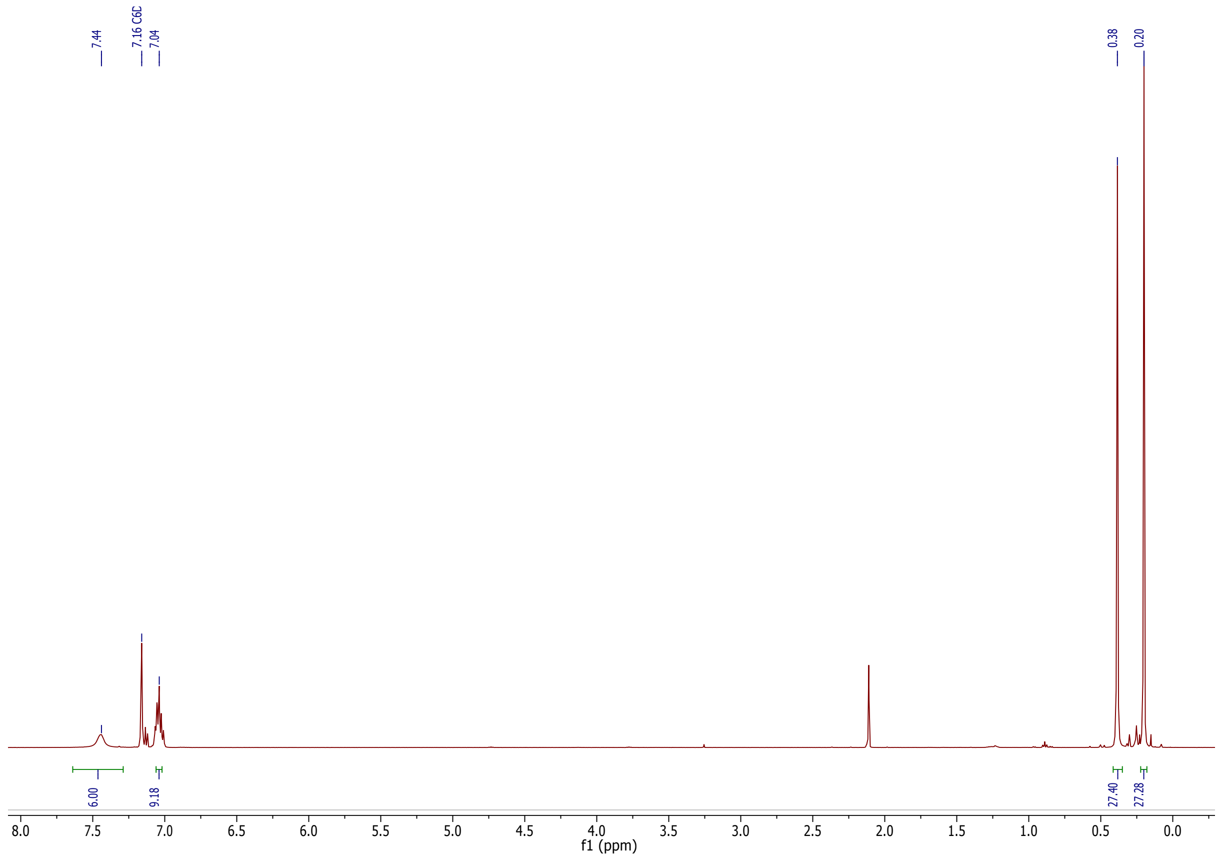

Figure S3. ^{1}H NMR spectrum for $U(OCP)L_3$ **3** (C_6D_6, 293K, 400MHz).

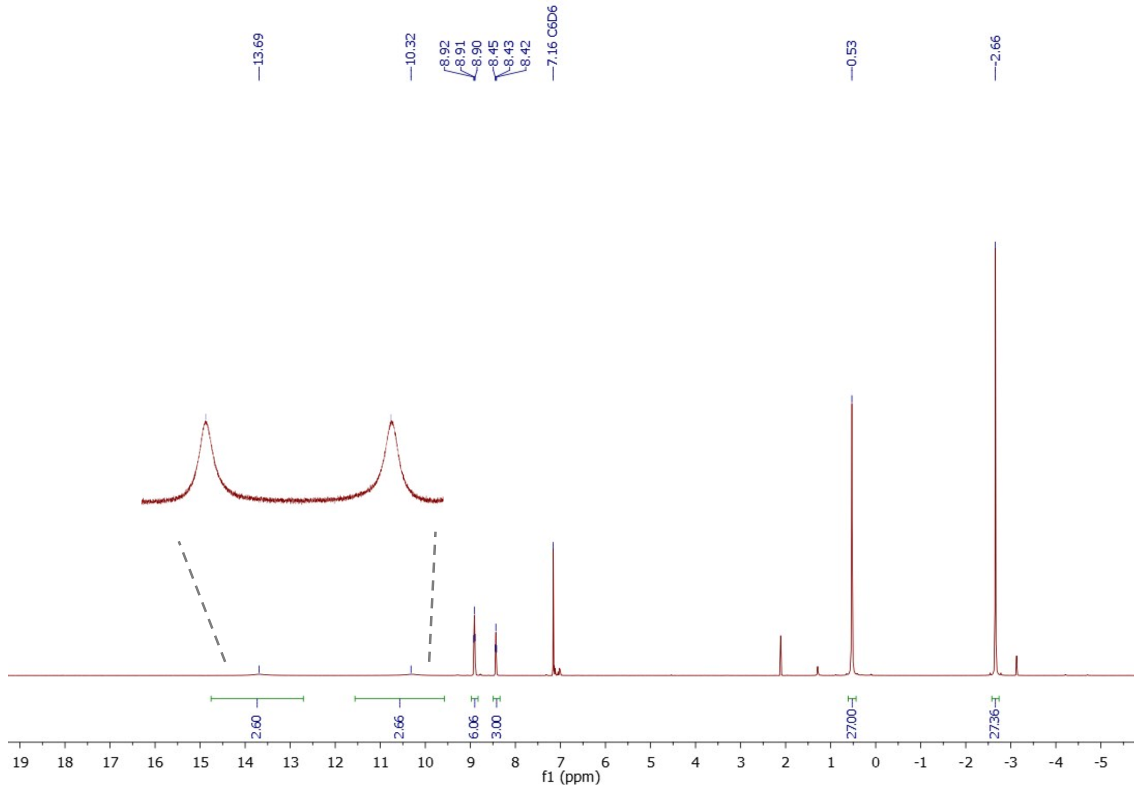

Figure S4. ^{1}H NMR spectrum for $Th(OCP)L_3$ **4** (C_6D_6, 293K, 400MHz).

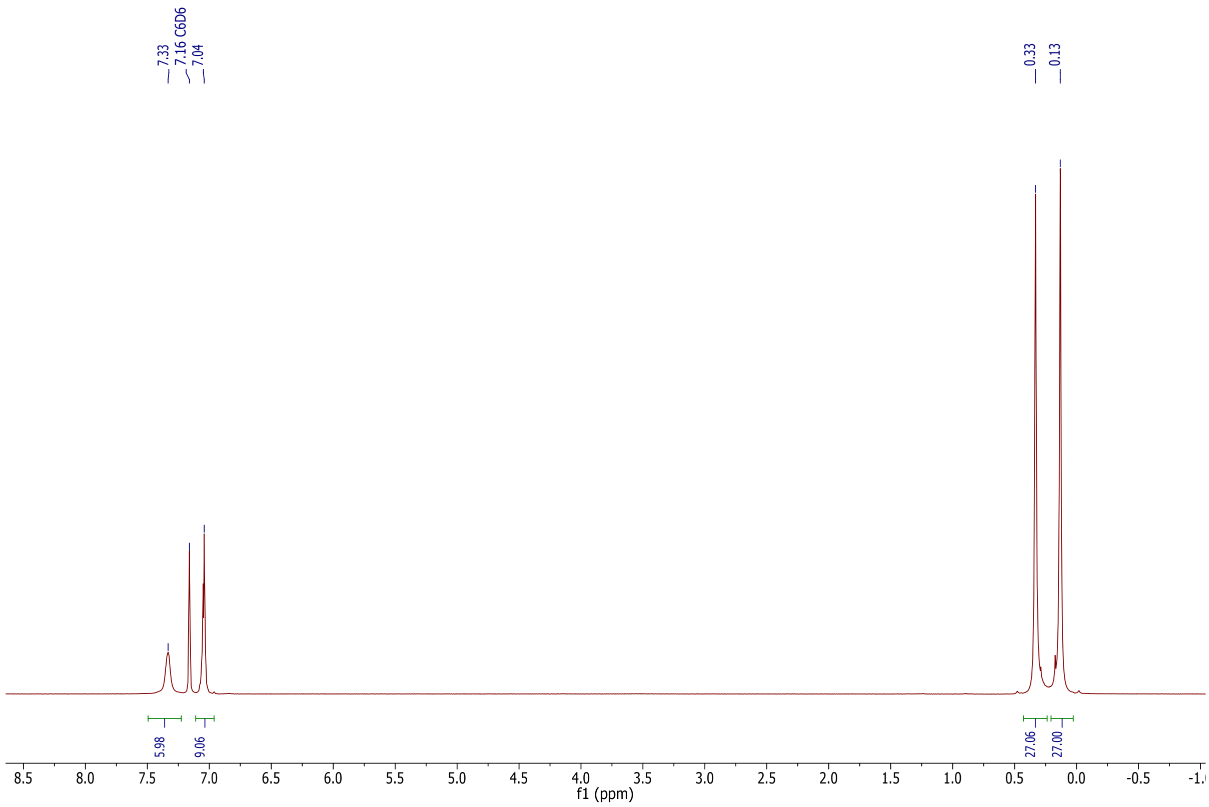

Figure S5. ^{31}P NMR spectrum for U(OCP)L_3 **3** (C_6D_6, 293K, 243MHz).

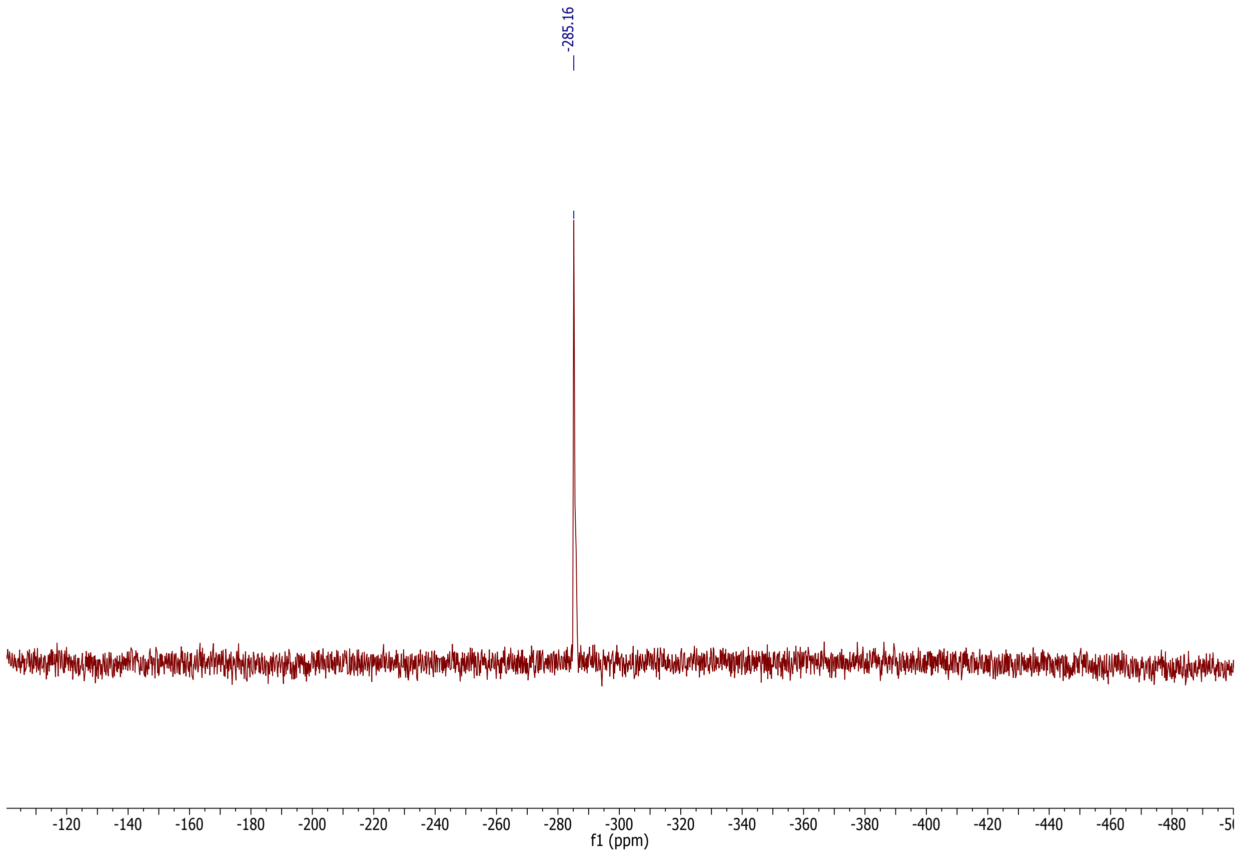

Figure S6. ^{31}P NMR spectrum for Th(OCP)L_3 **4** (C_6D_6, 293K, 243MHz).

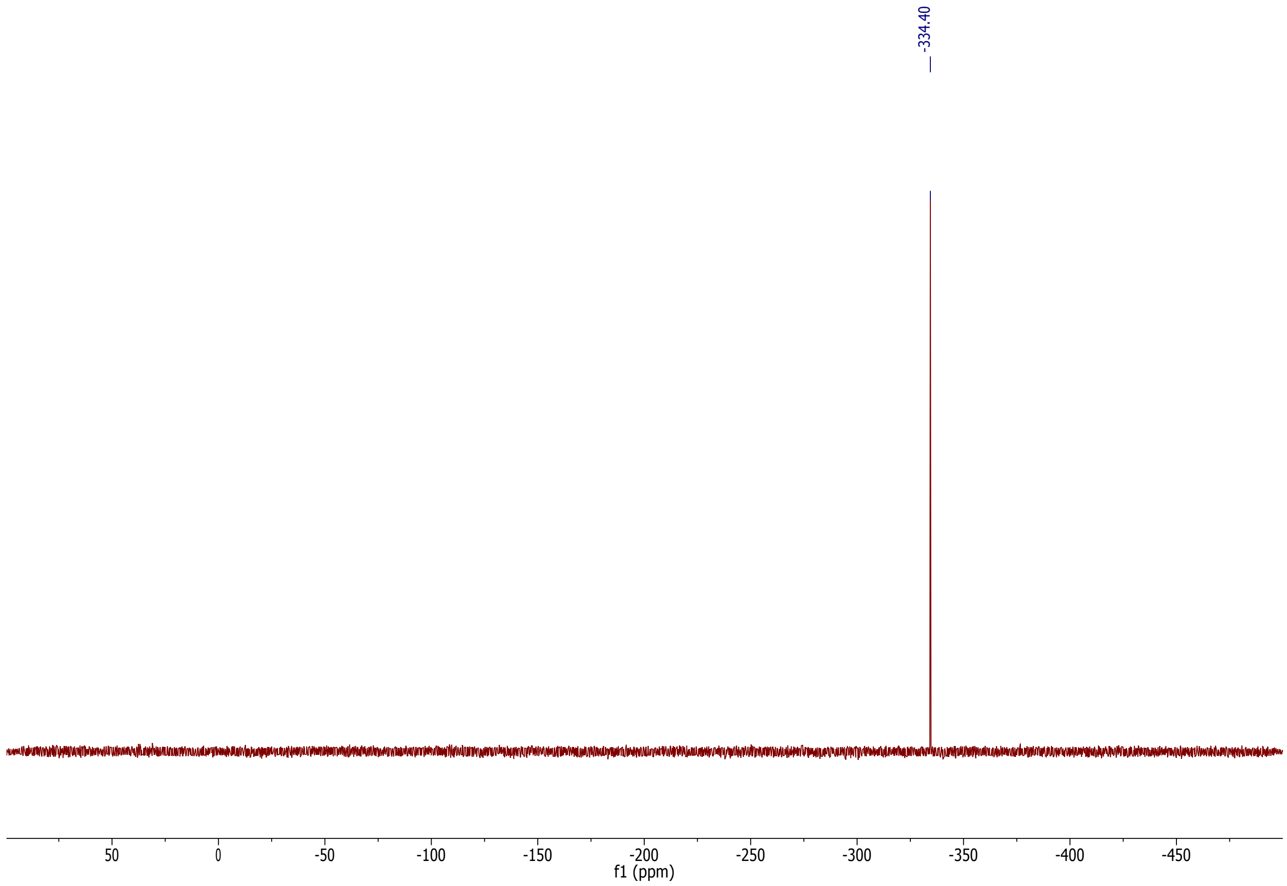

Figure S7. ^{1}H NMR spectrum for U(NCO)L$_3$ **5** (C_6D_6, 293K, 400MHz).

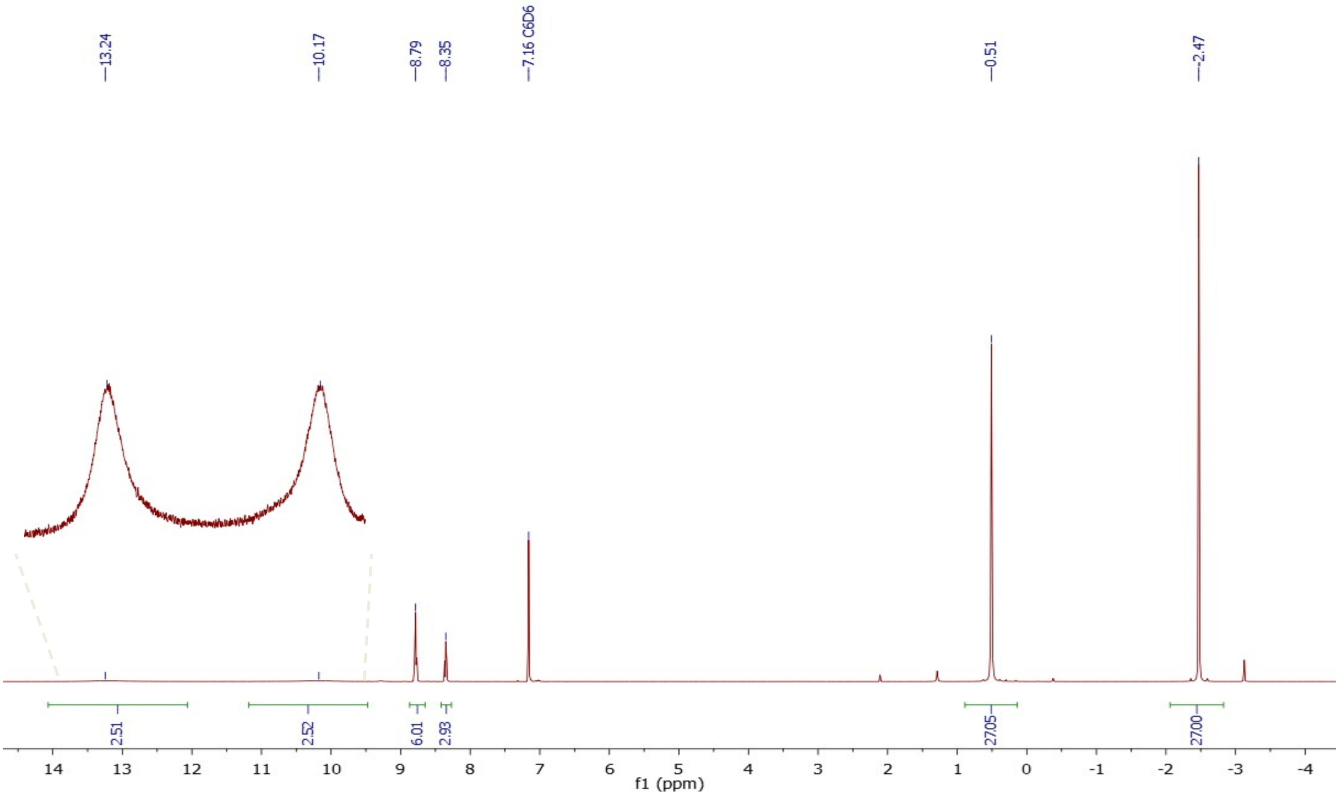

Figure S8. ^{1}H NMR spectrum for Th(NCO)L$_3$ **6** (C_6D_6, 293K, 400MHz).

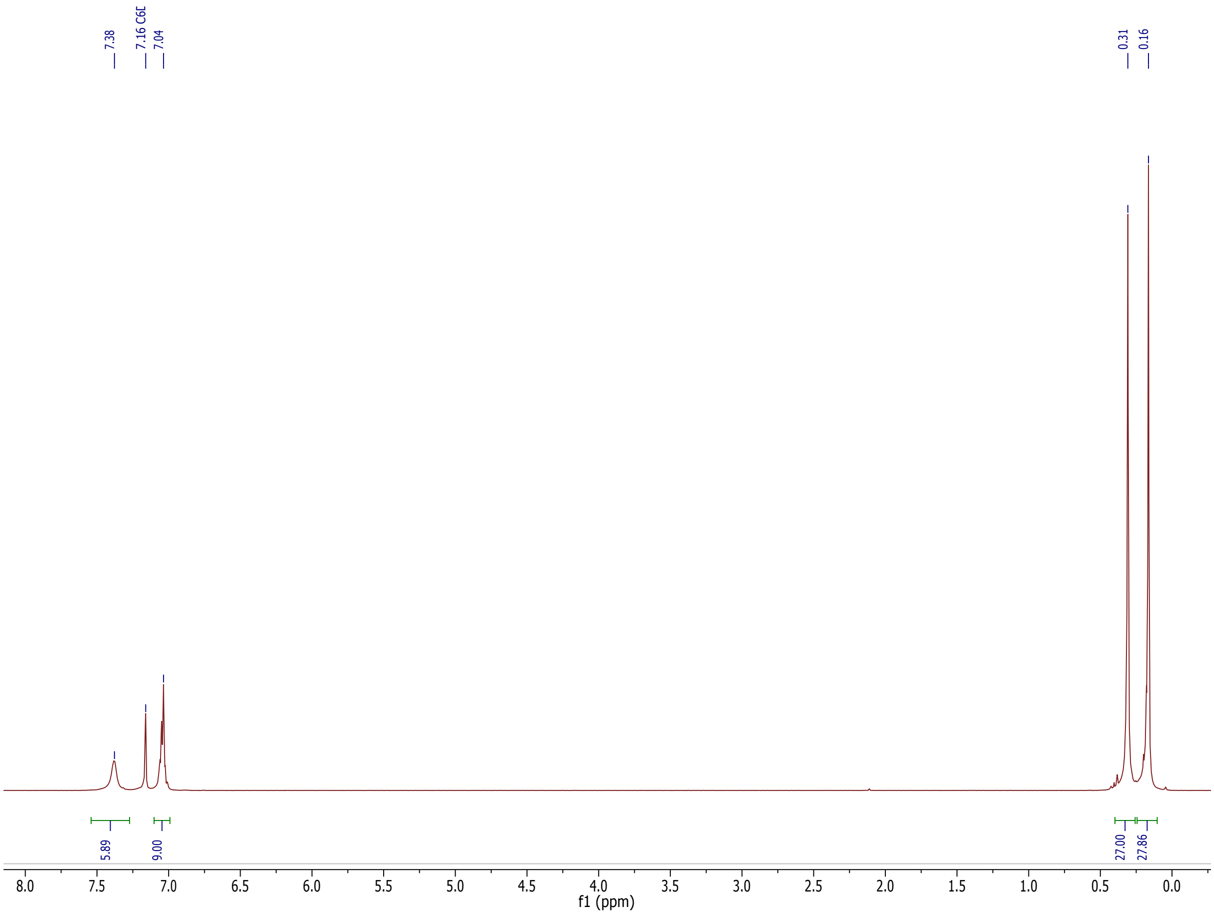

Figure S9. ^{1}H NMR spectrum for U(NCS)L_3 **7** (C_6D_6, 293K, 400MHz).

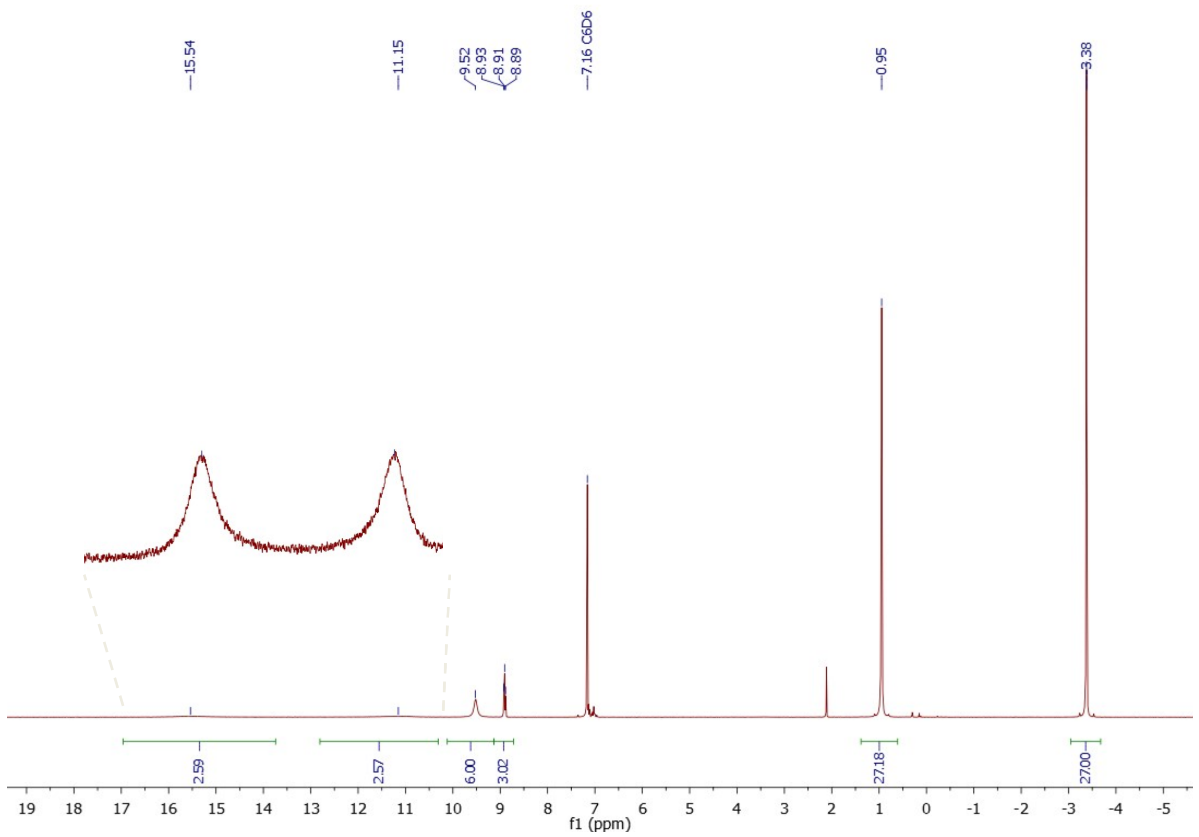

Figure S10. ^{1}H NMR spectrum for Th(NCS)L_3 **8** (C_6D_6, 293K, 400MHz).

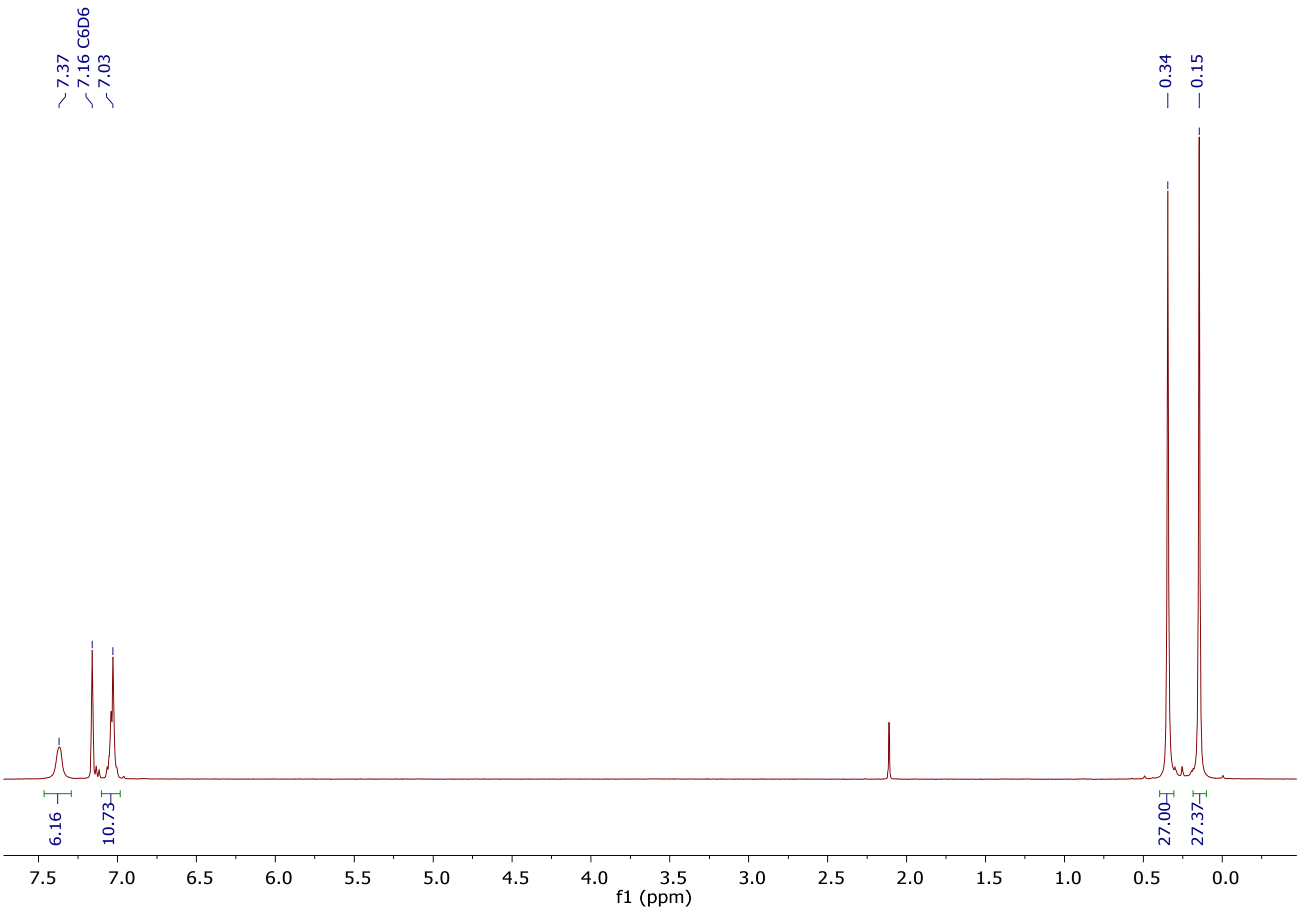

Figure S11. FT-IR spectra (nujol mull) for $UClL_3$ **1** (red), $U(OCP)L_3$ **3** (green), $U(NCO)L_3$ **5** (blue) and $U(NCS)L_3$ **7** (orange) ; L = amid.

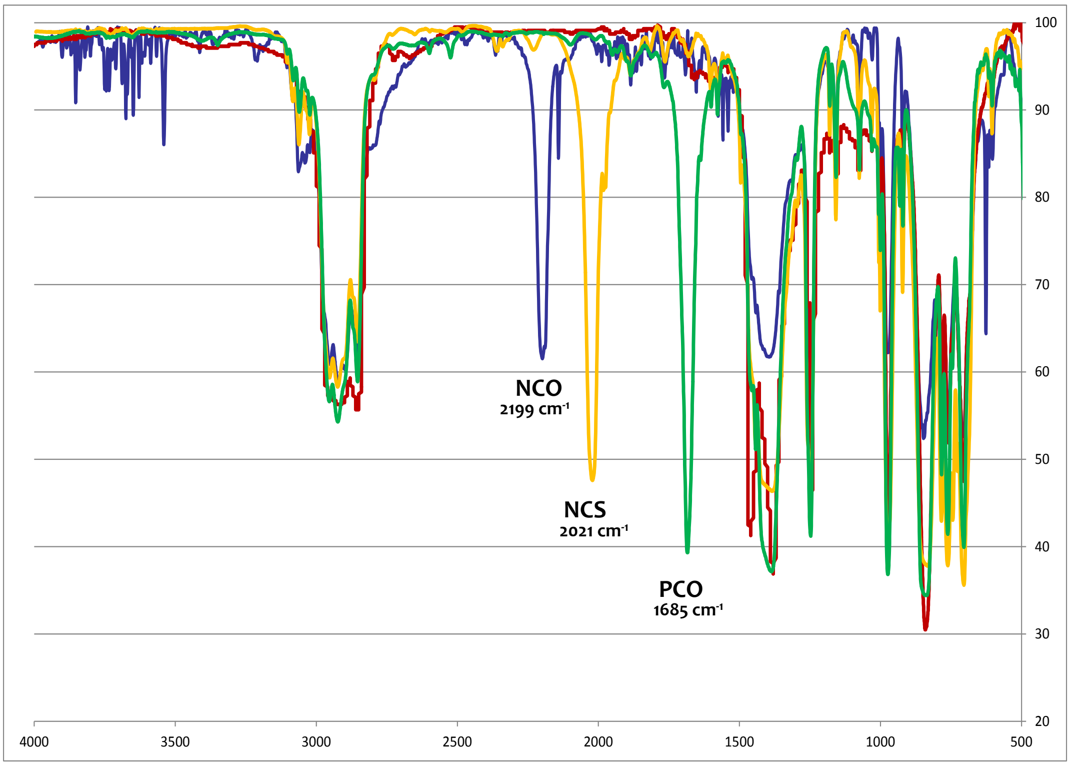

Figure S12. FT-IR spectra (nujol mull) for $ThClL_3$ **2** (red), $Th(OCP)L_3$ **4** (green), $Th(NCO)L_3$ **6** (blue) and $Th(NCS)L_3$ **8** (orange) ; L = amid.

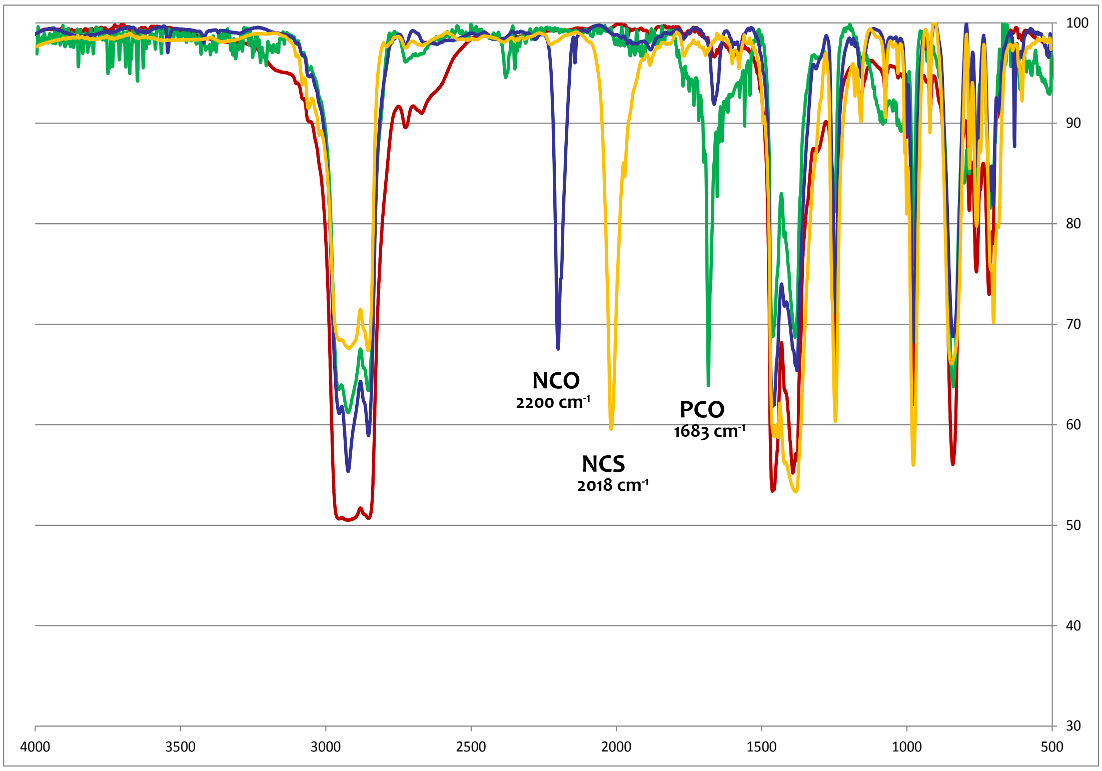

Table S.1 Infrared stretches for the anisotropic vibration of the heteroallene moiety in compounds **3-8**.

Compd	**3**	**4**	**5**	**6**	**7**	**8**
IR stretch (cm^{-1})	**1685**	**1683**	**2199**	**2200**	**2021**	**2018**

Figure S13. In-situ ^{1}H NMR spectrum for the reaction between **4** and Ni(COD)$_2$, recorded after 24h at r.t. (C_6D_6, 293K, 600MHz). The spectrum features the resonances for **9**, **4**, Ni(COD)2 and free COD. The relative ratio **9**:**4** does not evolve further when the reaction is kept at r.t.

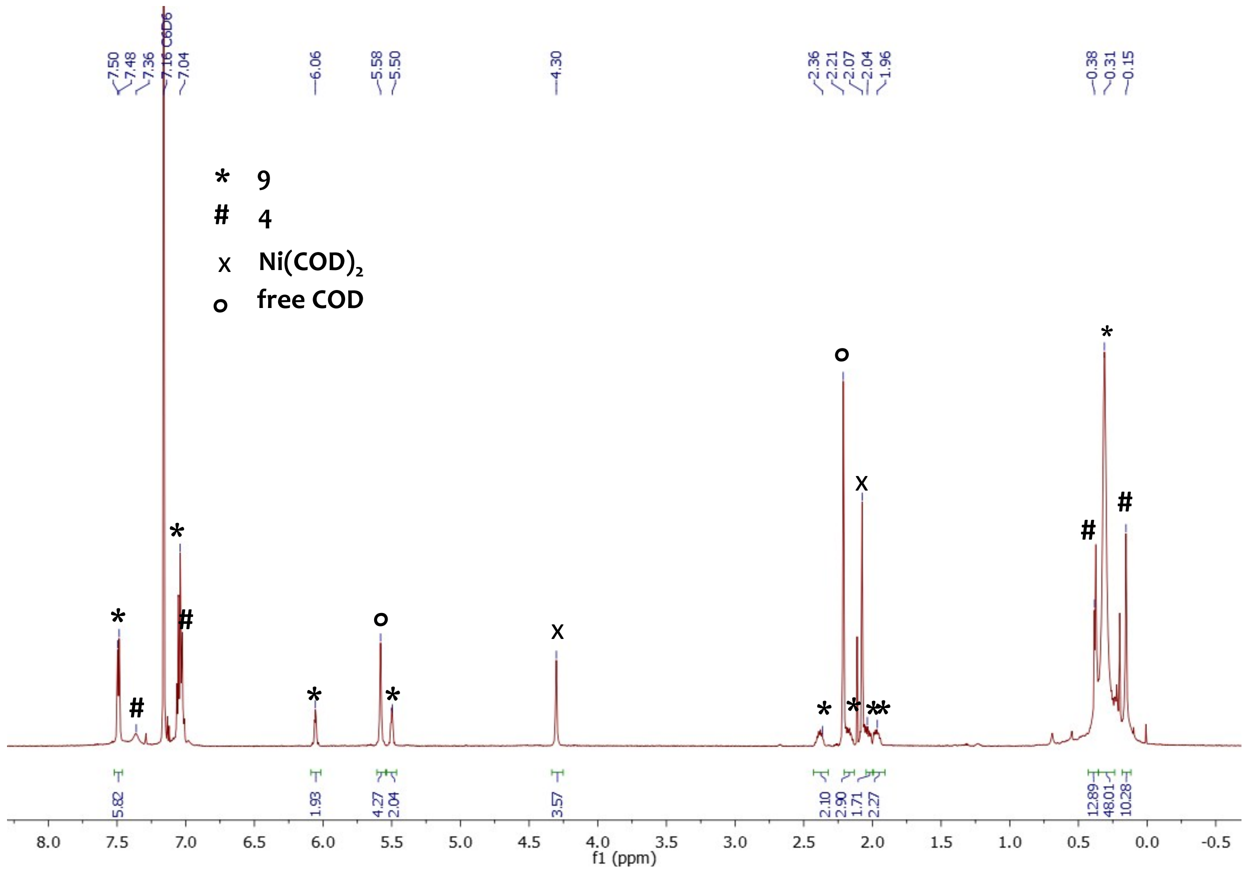

Figure S14. In-situ ^{31}P NMR spectrum for the reaction between **4** and Ni(COD)$_2$, recorded after 24h at r.t. (C_6D_6, 293K, 243MHz).

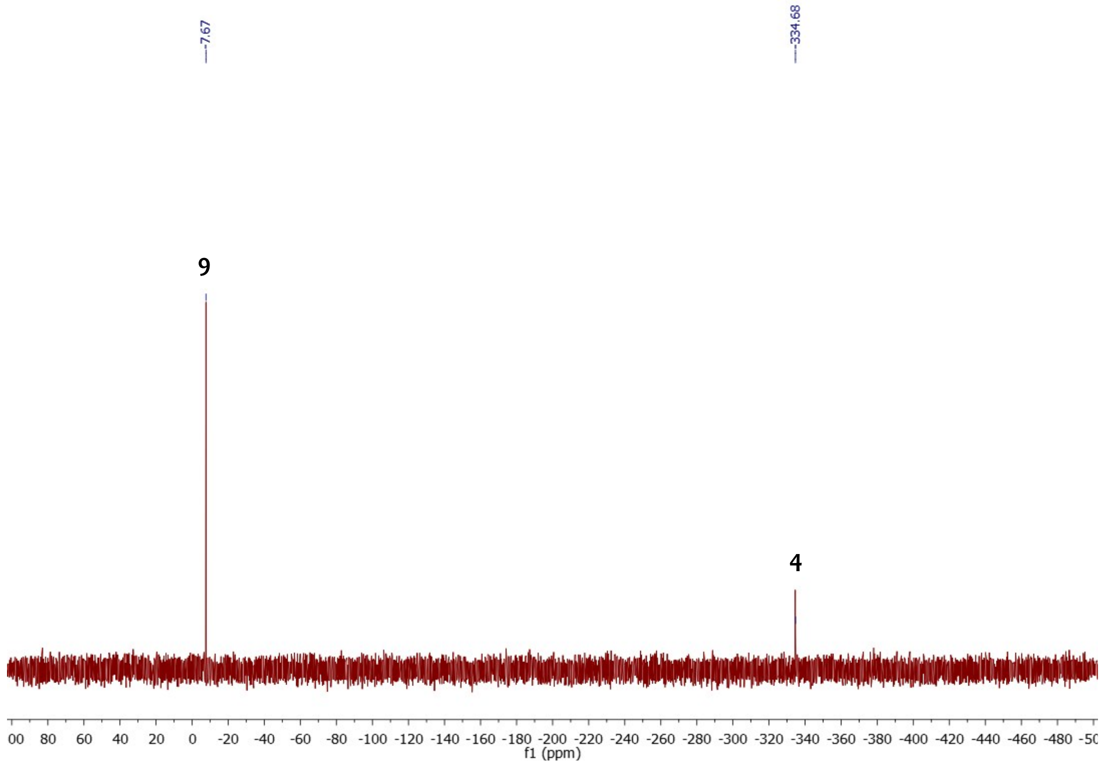

Figure S15. ^{1}H NMR spectrum for **9** (C_6D_6, 293K, 600MHz).

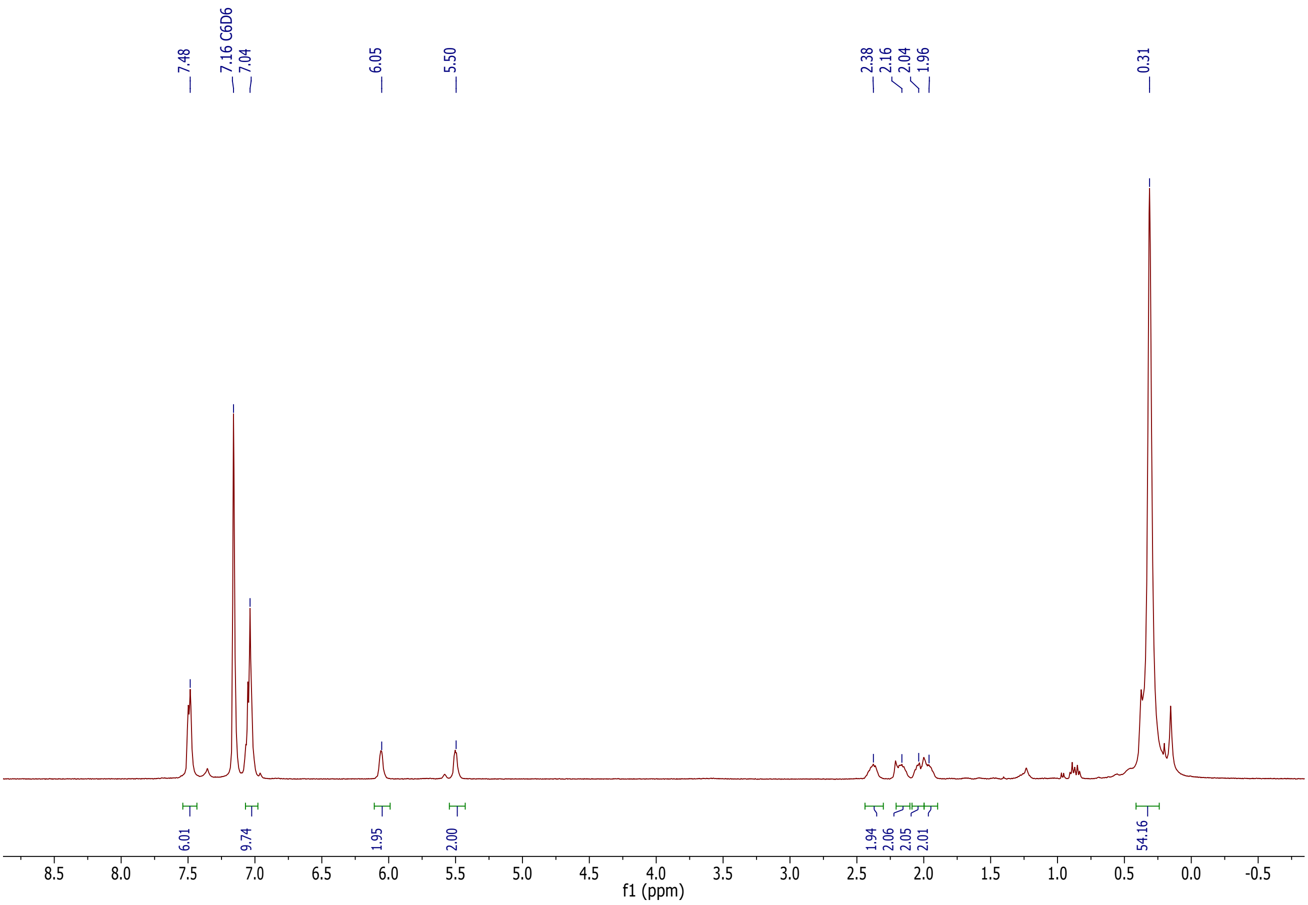

Figure S16. ^{1}H-^{1}H COSY NMR spectrum for **9** (C_6D_6, 293K, 600MHz).

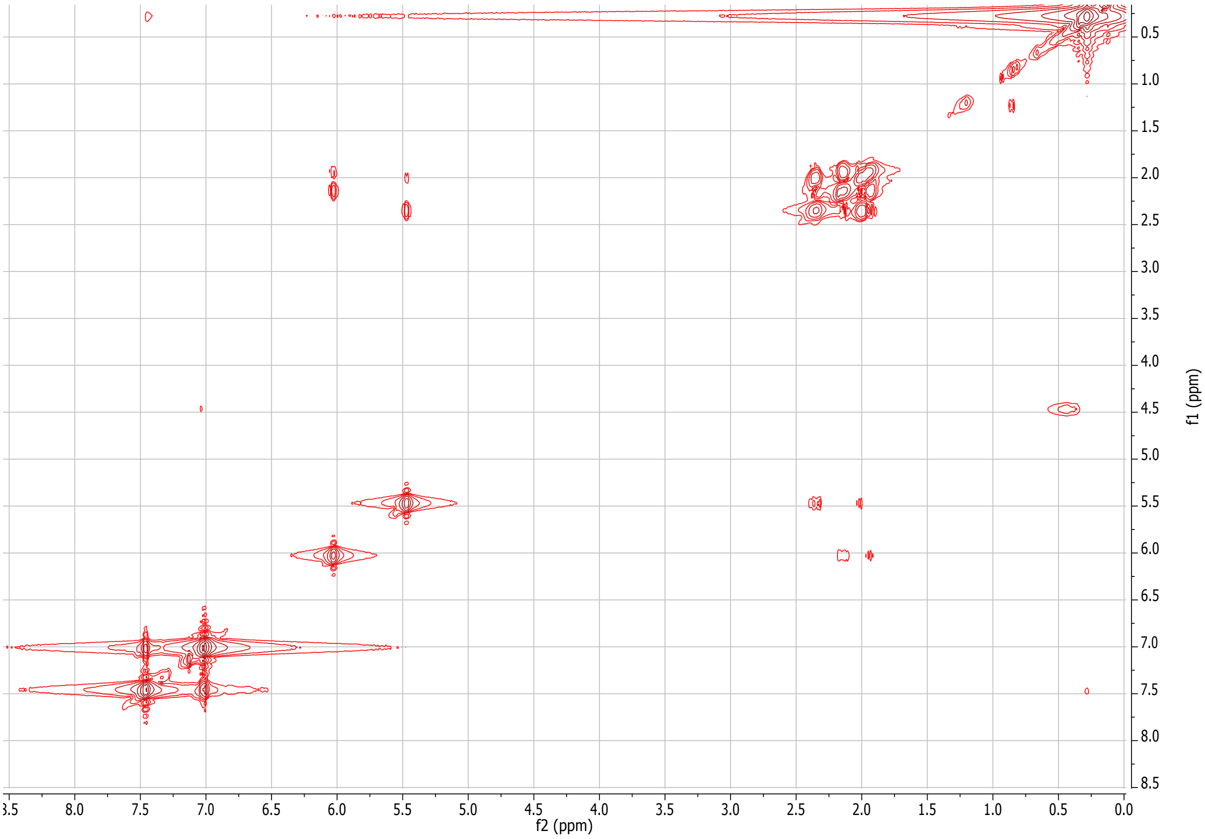

Figure S17. Evolution of the ^{31}P NMR spectrum for **9** (C_6D_6, 293 K, 600 MHz) over time showing that **9** is converted into **4**, releasing COD and $Ni(COD)_2$.

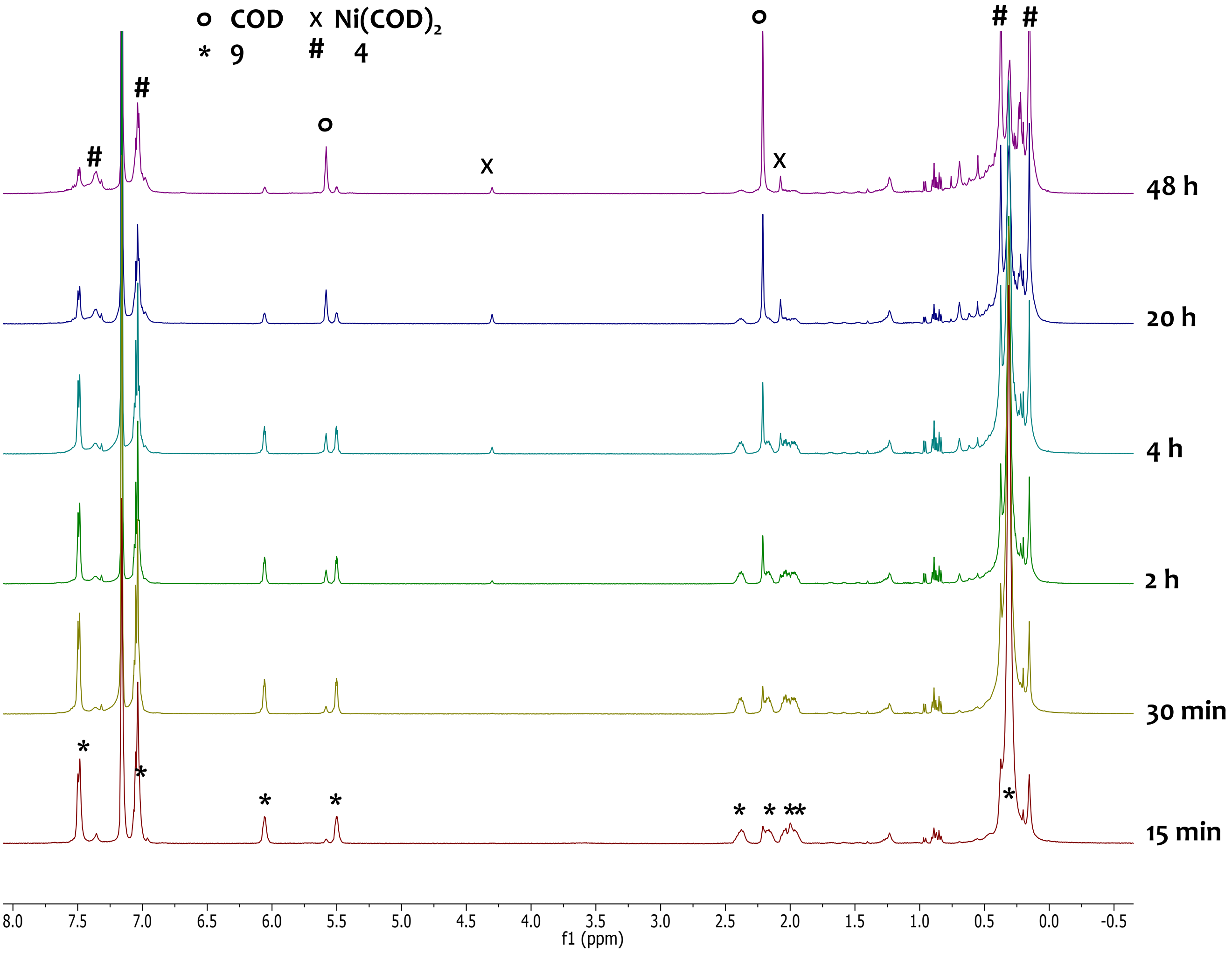

Figure S18. Evolution of the ^{31}P NMR spectrum for **9** (C_6D_6, 293 K, 243 MHz) over time.

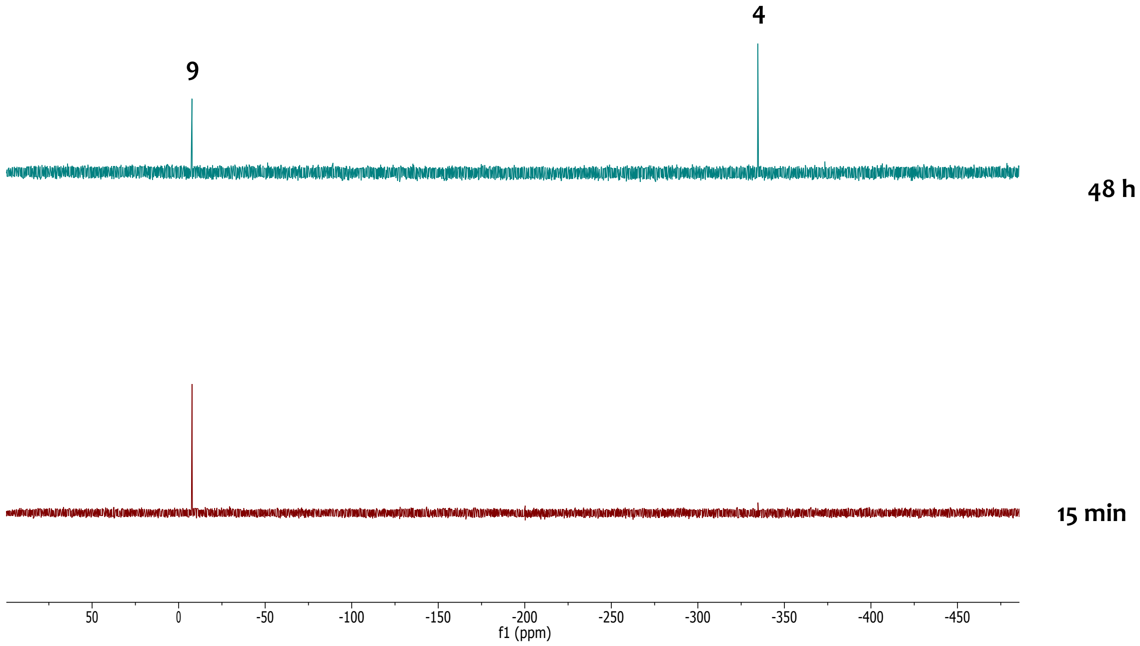

C. X-ray crystallography

X-ray structural determinations were performed on a Bruker SMART QUAZAR diffractometer which is a 3-circle diffractometer that couple a CCD detector with a sealed-tube source of monochromated Mo Ka radiation (λ = 0.71073 Å). A crystal of appropriate size was coated in Paratone-N oil and mounted on a Kapton® loop. The loop was transferred to the diffractometer, centered in the beam, and cooled by a nitrogen flow low-temperature apparatus that had been previously calibrated by a thermocouple placed at the same position as the crystal. Preliminary orientation matrices and cell constants were determined by collection of 60 10 s frames, followed by spot integration and least-squares refinement. The reported cell dimensions were calculated from all reflections with $I > 10\ \sigma$. The data were corrected for Lorentz and polarization effects; no correction for crystal decay was applied. An empirical absorption correction based on comparison of redundant and equivalent reflections was applied using SADABS.[6] All software used for diffraction data processing and crystal-structure solution and refinement are contained in the APEX2 program suite (Bruker AXS, Madison, WI).[7] Thermal parameters for all non-hydrogen atoms were refined anisotropically. For all structures, $R_1 = \Sigma(|F_o| - |F_c|)/\Sigma(|F_o|)$; $wR_2 = [\Sigma\{w(F_o^2 - F_c^2)^2\}/\Sigma\{w(F_o^2)^2\}]^{1/2}$. Thermal ellipsoid plots were created using Mercury supplied with Cambridge Structural Database (CCDC: Cambridge, U.K., 2004-2009). In the case of **5** and **6**, both O- and N- coordination to the metal were proposed and refined. Improved ellipsoid e.s.d's values were found at the O and N positions for the An-N=C=O coordination mode compared to the An-O=C=N one, the latter featuring additional residual electron intensity located at close proximity of the misattributed N-atom. This is in agreement with N-coordination to the actinide metal in both cases. No evidence of any sort of disorder involving the An-NCO unit was found.

Table S.2 Experimental metrical parameters for complexes **3**-**9** determined by single-crystal X-ray diffraction.

Compound	**3**	**4**	**5**	**6**	**7**	**8**	**9**
An-X	2.297(3)	2.3118(2)	2.340(3)	2.410(2)	2.385(4)	2.428(4)	2.279(3)
X-C1	1.219(6)	1.246(4)	1.128(5)	1.146(3)	1.165(7)	1.169(6)	1.287(5)
C1-Y	1.576(5)	1.561(4)	1.211(5)	1.206(3)	1.609(5)	1.608(5)	1.660(4)
An-$N_{amidavg}$	2.44(3)	2.49(3)	2.44(2)	2.50(5)	2.43(3)	2.49(4)	2.51(5)
Ni1-C1	/	/	/	/	/	/	1.895(4)
Ni1-P1	/	/	/	/	/	/	2.1705(13)
An-X-C1	170.9(3)	176.4(3)	171.6(3)	177.1(2)	171.3(4)	169.6(4)	157.5(3)
X-C1-Y	179.1(4)	179.7(4)	179.2(5)	179.5(3)	179.3(5)	178.7(5)	148.1(3)

*Distances are in [Å] and angles in [°]. Complexes **4, 6** and **9** crystallized as two independent molecules in the asymmetric unit, therefore the discussion of metrical parameters for these compounds is performed on the Th1 molecule only.*

Table S.3 Calculated metrical parameters for complexes **3**-**8** determined by DFT.

Compound	**3**	**4**	**5**	**6**	**7**	**8**
An-X	2.30	2.32	2.32	2.34	2.38	2.41
X-C1	1.24	1.24	1.20	1.27	1.19	1.19
C1-Y	1.60	1.60	1.18	1.18	1.63	1.63
An-X-C1	177.55	179.21	179.09	178.12	179.62	179.01
X-C1-Y	179.44	179.85	179.82	179.97	179.91	179.90

Distances are in [Å] and angles in [°].

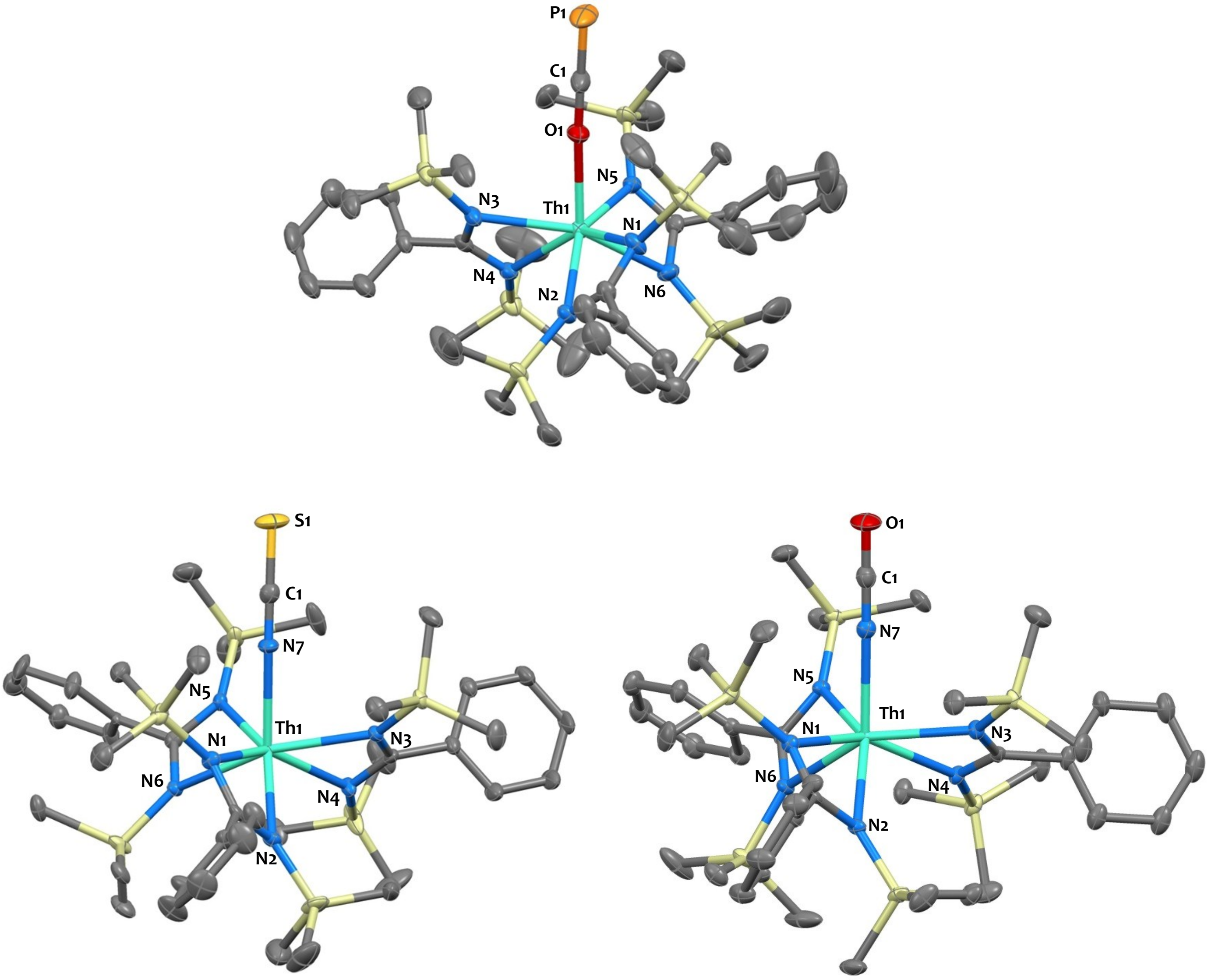

Figure S19. Solid-state molecular structures of compounds **4** (top), **6** (bottom-left) and **8** (bottom-right) determined by single-crystal X-ray diffraction. Ellipsoids are represented with 50% probability.

Table S.4 Crystallographic parameters for complexes **3** to **6**.

Compound	**3.(toluene)$_{0.5}$**	**4**	**5**	**6.(toluene)$_{0.5}$**
Formula	$C_{43.5}H_{73}N_6OPSi_6U$	$C_{40}H_{69}N_6OPSi_6Th$	$C_{43.5}H_{73}N_6OPSi_6U$	$C_{43.5}H_{73}N_6OPSi_6Th$
cryst syst	Triclinic	Triclinic	Monoclinic	Triclinic
space group	P-1	P-1	C2/c	P-1
volume (Å^3)	2750.1(5)	5882.7(8)	10388.9(8)	5419.7(16)
a (Å)	11.5703(12)	11.4301(9)	15.5201(7)	11.2966(19)
b (Å)	11.7055(13)	21.3302(16)	17.9868(8)	23.057(4)
c (Å)	21.152(2)	24.614(2)	37.3806(17)	23.270(4)
α (deg)	86.747(5)	99.881(4)	90	66.405(6)
β (deg)	83.810(5)	90.073(4)	97.783(2)	87.390(6)
γ (deg)	75.030(5)	95.606(4)	90	77.614(6)
Z	2	4	4	4
formula weight (g/mol)	1133.61	1081.56	2141.18	1110.66
density (g cm^{-3})	1.369	1.221	1.376	1.361
absorption coefficient (mm^{-1})	3.146	2.715	3.314	2.921
F(000)	1150	2192	4336	2260
temp (K)	100(2)	100(2)	100(2)	100(2)
total no. reflections	67609	89182	90811	86355
unique reflections [R(int)]	10048 [0.033]	21550 [0.036]	9521 [0.034]	19891 [0.033]
Final R indices [I > 2σ(I)]	R1 = 0.0279, wR2 = 0.0702	R1 = 0.0282, wR2 = 0.0683	R1 = 0.0285, wR2 = 0.0545	R1 = 0.0195, wR2 = 0.0482
Largest diff. peak and hole (e.A^{-3})	0.865 and -2.201	3.278 and -0.874	1.204 and -1.664	2.311 and -0.454
GoF	1.371	0.975	1.319	1.019

Table S.5 Crystallographic parameters for complexes **7** to **9**.

Compound	**7.(toluene)$_{0.5}$**	**8.(toluene)$_{0.5}$**	**9.(hexane)$_{0.75}$**
Formula	$C_{43.5}H_{73}N_7SSi_6U$	$C_{43.5}H_{73}N_7SSi_6Th$	$C_{52.5}H_{91.5}N_6NiOPSi_6Th$
cryst syst	Triclinic	Triclinic	Triclinic
space group	P-1	P-1	P-1
volume (Å^3)	2733.3(3)	2759.5(2)	6587.8 (8)
a (Å)	11.5810(7)	11.6047(6)	14.8598(10)
b (Å)	11.6278(7)	11.6602(6)	19.6824(13)
c (Å)	21.1150(13)	21.2040(11)	24.2681(17)
α (deg)	86.775(2)	86.479(2)	105.8698(27)
β (deg)	83.982(2)	83.836(2)	101.3208(26)
γ (deg)	75.259(3)	75.464(2)	96.5472(27)
Z	2	2	4
formula weight (g/mol)	1132.71	1126.72	1313.07
density (g cm^{-3})	1.376	1.356	1.324
absorption coefficient (mm^{-1})	3.174	2.905	2.711
F(000)	1150	1146	2694
temp (K)	100(2)	100(2)	100(2)
total no. reflections	45826	50449	171492
unique reflections [R(int)]	9978 [0.024]	10054 [0.028]	23909 [0.031]
Final R indices [I > 2σ(I)]	R1 = 0.0294, wR2 = 0.0796	R1 = 0.0278, wR2 = 0.0781	R1 = 0.0333, wR2 = 0.0857
Largest diff. peak and hole (e.A^{-3})	1.005 and -2.243	1.059 and -2.196	3.024 and -3.064
GoF	1.383	1.417	1.034

D. DFT calculations

Computational details

Calculations were carried out at the DFT level using the hybrid functional B3PW91[8,9] with the Gaussian 03[10] suite of programs. Polarized all-electron triple-ζ 6-31G(d,p)[11] basis sets were used for C, H and N. Thorium, Uranium, Phosphorus and silicon atoms were treated with an effective core potential from the Stuttgart-Dresden-Köln group in association with its adapted basis set.[12] Geometry optimizations were carried out without any symmetry restriction. The nature of the extrema (minimum) was verified with analytical frequency calculations. NBO analysis[13] was also carried out in order to analyze the bonding.

CONiPThL3xyz
145
scf done: -2890.127887
Th 12.499578 6.614290 10.103720
P 10.170644 7.755060 11.525925
Ni 8.408660 8.911315 12.278902
C 8.247831 9.232817 14.490101
C 7.633064 8.062400 14.143600
C 6.168562 7.870355 13.829077
C 5.544196 9.013957 12.996505
C 6.491939 9.570773 11.951600
C 7.352795 10.648098 12.135266
C 7.558692 11.430810 13.411031
C 7.564100 10.565502 14.690737
N 12.914482 4.132757 9.685574
Si 14.163965 2.892549 10.013878
C 13.766559 1.857122 11.547941
N 10.832596 4.974395 9.230488
Si 9.139153 5.011170 8.613870
C 8.995261 6.600529 7.613167
N 12.977019 7.221000 7.685359
C 12.798873 8.523721 7.937652
C 12.624260 9.519705 6.826015
C 13.724504 9.979282 6.093463
C 13.543982 10.884469 5.048557
C 12.263012 11.328320 4.721577
C 11.165077 10.876703 5.453725
C 11.341957 9.982643 6.507880
N 14.821989 7.024930 11.129941
C 14.379201 6.724773 12.354064
C 15.195814 7.018192 13.583878
C 16.270082 6.199472 13.948672
C 17.010651 6.477398 15.096808
C 16.690572 7.579835 15.887938

C 15.620525 8.399533 15.530213
C 14.872332 8.117445 14.388946
N 13.161857 6.182596 12.465100
Si 12.358488 5.557057 13.941648
C 10.984594 4.423733 13.316401
N 12.732834 8.909289 9.216149
Si 12.749519 10.594695 9.855177
C 11.235154 11.640464 9.430355
C 11.620894 3.913980 9.408026
C 11.052887 2.521023 9.333037
C 10.239654 2.040605 10.367328
C 9.695489 0.759334 10.299985
C 9.942050 -0.050291 9.191451
C 10.736689 0.428596 8.151185
C 11.293233 1.704571 8.222785
C 14.525921 1.716892 8.566464
C 15.772987 3.828746 10.297543
C 8.714162 3.602294 7.412553
C 7.886973 4.969859 10.027121
Si 13.181778 6.454844 6.076518
C 12.725288 4.633304 6.240694
C 12.110778 7.161923 4.681412
C 15.002318 6.525002 5.549241
Si 16.391728 7.791062 10.706517
C 16.722238 9.409390 11.635494
C 17.907487 6.683394 10.991215
C 16.325207 8.134137 8.853061
C 11.597864 6.934896 14.988558
C 13.507676 4.514456 15.032934
C 12.873093 10.429564 11.731610
C 14.280250 11.532267 9.237231
H 6.303173 9.271170 10.921933
H 7.744556 11.119858 11.235347
H 6.801547 12.225232 13.505678
H 8.522886 11.945778 13.327767
H 8.081572 11.115339 15.485039
H 6.543450 10.409336 15.050951
H 9.297818 9.179163 14.771666
H 8.227530 7.152916 14.198457
H 6.075040 6.934777 13.266752
H 5.591487 7.725240 14.755365
H 5.197239 9.818416 13.651947
H 4.648067 8.629210 12.497131
H 11.219824 12.509014 10.100058
H 11.273011 12.009236 8.402183
H 10.306403 11.082121 9.563965

H 13.037483 11.415749 12.180806
H 11.953650 10.002523 12.143078
H 13.710614 9.785619 12.013826
H 14.334526 12.492892 9.763375
H 15.211329 10.993969 9.432393
H 14.222170 11.744114 8.166374
H 14.722814 9.636603 6.349619
H 14.404811 11.241433 4.489725
H 12.121687 12.027705 3.902197
H 10.165311 11.223149 5.207054
H 10.488576 9.641809 7.087431
H 12.976701 4.113906 5.309052
H 13.268205 4.161719 7.062480
H 11.654459 4.512053 6.422218
H 12.169396 6.470677 3.831804
H 11.059692 7.239322 4.973745
H 12.440981 8.145954 4.341900
H 15.120402 6.004483 4.591701
H 15.364781 7.547692 5.417883
H 15.646088 6.026054 6.280077
H 8.006479 6.646679 7.142230
H 9.109382 7.504488 8.216187
H 9.743925 6.608861 6.814870
H 7.751032 3.856683 6.952697
H 9.445047 3.503474 6.604339
H 8.607827 2.630359 7.898993
H 6.877183 5.109930 9.624178
H 7.909760 4.010561 10.553380
H 8.092184 5.772646 10.740965
H 11.895056 2.079845 7.401600
H 10.920658 -0.189579 7.276666
H 9.511828 -1.046394 9.136265
H 9.073757 0.395835 11.113632
H 10.036839 2.672830 11.226025
H 14.597616 1.174353 11.759317
H 13.625735 2.496775 12.423891
H 12.863308 1.256017 11.412792
H 15.499671 1.249221 8.755776
H 13.785728 0.920859 8.467881
H 14.598379 2.245821 7.611034
H 16.574273 3.120241 10.535755
H 16.065188 4.375373 9.395043
H 15.689358 4.551015 11.110168
H 10.424231 4.010046 14.162394
H 10.282641 4.967294 12.676186
H 11.407960 3.587250 12.752815

H 10.921825 6.499010 15.733233
H 12.357995 7.513795 15.519344
H 11.020927 7.605822 14.345446
H 12.889421 3.960473 15.749417
H 14.076393 3.782112 14.451869
H 14.215064 5.124403 15.599597
H 14.040409 8.757977 14.112276
H 15.366334 9.261855 16.140459
H 17.270833 7.797816 16.780145
H 17.836797 5.828452 15.374243
H 16.516642 5.333538 13.341832
H 17.616904 9.879815 11.210233
H 15.898546 10.121470 11.547394
H 16.910195 9.234055 12.697861
H 18.764128 7.162460 10.501741
H 18.153816 6.572720 12.049705
H 17.787633 5.689029 10.554794
H 17.247558 8.636148 8.540236
H 16.237805 7.206627 8.281099
H 15.479699 8.774126 8.594284
C 9.083329 8.815963 10.555992
O 8.774230 9.303417 9.493754

NiPCOThL3xyz

145
scf done : -2890.14586603
C 9.451190 -0.190988 14.160841 -0.120551
C 8.871323 -0.069165 12.893155 -0.033466
C 8.536540 -1.226447 12.180649 -0.118054
C 8.768749 -2.484825 12.732197 -0.128960
C 9.346834 -2.600577 13.996221 -0.116996
C 9.690593 -1.451046 14.706850 -0.131004
C 8.606520 1.283903 12.291867 0.412912
N 7.569127 2.031478 12.684536 -0.673109
Th 7.931414 3.695942 10.751205 62.142223
O 8.142060 2.714409 8.693715 -0.581771
C 8.630258 2.466208 7.530199 -0.182624
Ni 8.481223 1.204001 6.159650 9.548993
P 9.752683 3.032121 6.353161 10.223744
N 9.388521 1.759311 11.323258 -0.679776
Si 10.914170 1.107406 10.650644 10.531438
C 12.105063 0.451506 11.973596 -0.536425
Si 6.387894 1.637083 13.968509 10.541046
C 4.859754 2.693785 13.656253 -0.528808
C 11.724282 2.574964 9.788005 -0.548064

C 10.630182 -0.256191 9.375235 -0.533326
C 5.817624 -0.171118 14.014483 -0.530198
C 7.051765 2.111335 15.681975 -0.526659
C 6.721186 0.095347 6.228645 -0.078318
C 6.229535 -0.218031 4.829006 -0.262671
C 6.946722 0.612314 3.741975 -0.246287
C 8.408352 0.858229 4.027812 -0.091266
C 9.302819 -0.041849 4.547663 -0.077518
C 9.007973 -1.489407 4.868463 -0.267689
C 8.587730 -1.693106 6.339861 -0.236866
C 7.736978 -0.573574 6.892543 -0.091846
N 5.506685 3.541748 10.260981 -0.686214
Si 4.513438 2.371269 9.349714 10.535344
C 2.730900 2.174394 9.965384 -0.530606
C 5.149523 4.690351 10.846692 0.401816
N 6.067266 5.343049 11.558752 -0.662910
Si 5.832590 6.887578 12.434630 10.540330
C 5.200460 6.558564 14.193609 -0.529081
C 3.751359 5.220038 10.672145 -0.026557
C 2.818702 5.131481 11.711447 -0.115603
C 1.523518 5.616650 11.539063 -0.131240
C 1.147849 6.201218 10.330111 -0.117266
C 2.072673 6.291105 9.290011 -0.130526
C 3.365268 5.798457 9.457317 -0.116337
C 4.461853 2.833035 7.515958 -0.542373
C 5.385270 0.716559 9.619457 -0.552824
C 7.516130 7.715767 12.588160 -0.524389
C 4.690309 8.146431 11.592507 -0.529878
N 9.014384 5.693713 9.778085 -0.690024
Si 8.724175 6.716662 8.331698 10.544778
C 10.150232 6.795655 7.094848 -0.536603
C 9.899030 5.866826 10.767022 0.410258
N 9.762031 5.121247 11.868031 -0.676776
Si 10.817866 5.103571 13.315351 10.542736
C 10.462360 3.487385 14.217758 -0.529035
C 11.038450 6.838030 10.616124 -0.036233
C 11.043861 8.054380 11.308802 -0.120442
C 12.117439 8.934419 11.177896 -0.133464
C 13.199907 8.603531 10.363967 -0.115423
C 13.197761 7.394360 9.669000 -0.130592
C 12.119687 6.519478 9.785780 -0.116995
C 7.219839 5.945193 7.492899 -0.551357
C 8.282993 8.492238 8.831776 -0.534739
C 12.678815 5.174726 12.960328 -0.534528
C 10.408470 6.494223 14.540543 -0.525545
H 6.069011 0.719422 6.832689 0.149381

H 7.785863 -0.453304 7.973437 0.142779
H 8.072985 -2.660785 6.451116 0.130338
H 9.488548 -1.758711 6.959962 0.150490
H 9.904185 -2.088360 4.670929 0.136446
H 8.237838 -1.872271 4.192729 0.130399
H 10.346178 0.263446 4.591319 0.171049
H 8.806246 1.801234 3.661019 0.175888
H 6.459635 1.590803 3.667668 0.154226
H 6.819057 0.134430 2.758331 0.133706
H 6.336842 -1.288341 4.627138 0.129553
H 5.154622 -0.011674 4.773717 0.133072
H 3.973919 2.042030 6.934827 0.131072
H 3.888703 3.752444 7.363591 0.131902
H 5.468638 2.986498 7.117121 0.178185
H 4.917332 -0.082080 9.034469 0.137688
H 6.437984 0.781923 9.329994 0.164811
H 5.334089 0.436341 10.676448 0.146721
H 2.301198 1.295566 9.469096 0.132885
H 2.679645 2.000470 11.044315 0.133069
H 2.102736 3.035961 9.728079 0.149821
H 3.103932 4.663301 12.647955 0.133674
H 0.805796 5.534601 12.350797 0.127426
H 0.139255 6.582721 10.198026 0.126460
H 1.788639 6.747791 8.345870 0.129154
H 4.083364 5.874896 8.646049 0.139434
H 7.457117 8.544838 13.302053 0.127441
H 8.272522 7.010322 12.933929 0.152247
H 7.837325 8.118386 11.624610 0.139571
H 4.830869 9.112332 12.092807 0.131850
H 4.948287 8.280919 10.537623 0.137260
H 3.631740 7.884842 11.651511 0.148132
H 5.179594 7.502030 14.751455 0.134427
H 4.188515 6.144172 14.209202 0.130088
H 5.860286 5.870198 14.731364 0.138217
H 6.886600 6.581102 6.665226 0.132390
H 7.460424 4.959731 7.083135 0.178390
H 6.384425 5.842548 8.192830 0.139820
H 8.097140 9.079720 7.924899 0.133838
H 7.376045 8.528826 9.441947 0.137430
H 9.093254 8.980139 9.380901 0.138767
H 9.774914 7.241351 6.165828 0.131923
H 10.979469 7.409914 7.453724 0.138166
H 10.520517 5.795335 6.853565 0.170897
H 10.200638 8.315462 11.940341 0.134787
H 12.106802 9.879597 11.713902 0.126538
H 14.039425 9.286462 10.268047 0.125519

H 14.036525 7.130600 9.030701 0.130570
H 12.116316 5.581205 9.239530 0.155377
H 13.208183 4.962814 13.897278 0.132039
H 12.990446 4.423277 12.229285 0.139568
H 13.005431 6.154158 12.603516 0.151398
H 11.057097 6.373541 15.416563 0.134722
H 10.585282 7.493156 14.134934 0.132870
H 9.372827 6.440988 14.889107 0.137087
H 10.971065 3.485375 15.188117 0.127168
H 9.390856 3.358401 14.389641 0.151368
H 10.817519 2.632452 13.638923 0.143655
H 12.714073 2.295362 9.410862 0.133920
H 11.128811 2.903857 8.928948 0.168931
H 11.847164 3.413563 10.478869 0.141115
H 11.598933 -0.567989 8.967359 0.136325
H 10.146241 -1.137389 9.805034 0.125295
H 10.026434 0.126781 8.546739 0.183122
H 13.052114 0.202926 11.479482 0.133886
H 12.325622 1.193473 12.746945 0.132614
H 11.734520 -0.452779 12.462207 0.148414
H 8.077653 -1.136452 11.200318 0.144504
H 8.495060 -3.376371 12.174597 0.128238
H 9.530160 -3.582058 14.424419 0.126146
H 10.149046 -1.533973 15.688534 0.127766
H 9.730596 0.702759 14.709734 0.137027
H 4.947884 -0.237532 14.679208 0.131856
H 5.506457 -0.522542 13.026003 0.135319
H 6.584356 -0.852233 14.390208 0.150105
H 6.259330 1.966690 16.425725 0.134437
H 7.906186 1.501434 15.986711 0.131216
H 7.350778 3.163675 15.718635 0.135902
H 4.150905 2.569261 14.482334 0.127463
H 5.125851 3.750763 13.582544 0.150389
H 4.364345 2.398827 12.728062 0.147003

ThClL3xyz

122
scf done: -2302.431792
Th 10.267832 5.440382 15.049147
N 8.457311 4.675123 16.553531
Si 7.763076 5.277780 18.094215
C 7.114259 3.884402 19.205760
N 11.707578 5.098419 12.971646
Si 13.328406 4.502798 12.496205
C 14.321371 4.245735 14.077171

N 9.710942 6.206800 12.775959
Si 8.329784 7.098467 12.054340
C 8.610725 8.966354 12.088812
N 12.253019 6.460634 16.116243
C 12.552064 5.391922 16.855580
C 13.540529 5.498891 17.987030
C 13.143251 6.054530 19.209978
C 14.054929 6.178352 20.257138
C 15.375280 5.762132 20.090680
C 15.778764 5.217248 18.872559
C 14.866534 5.082073 17.827099
N 11.920398 4.246922 16.572253
Si 12.071265 2.671625 17.405532
C 11.324621 2.714998 19.142789
N 8.689932 3.460652 14.622685
Si 8.230931 2.302965 13.328912
C 8.621481 0.508761 13.796434
Cl 9.197841 7.872073 15.686352
C 7.982659 3.744545 15.722604
C 6.672359 3.065367 16.010515
C 5.465227 3.741914 15.799619
C 4.250884 3.115893 16.074176
C 4.230215 1.812343 16.569896
C 5.430063 1.136279 16.788334
C 6.645778 1.758153 16.508527
C 9.189086 6.122193 18.998064
C 6.381771 6.537767 17.817739
C 10.870547 5.832291 12.228861
C 11.225442 6.254369 10.830506
C 6.834797 6.666255 13.124762
C 7.935448 6.559178 10.279794
C 13.199882 2.829844 11.612683
C 14.353741 5.681643 11.424106
Si 12.967133 8.105524 16.120275
C 12.562780 8.824284 14.420622
C 14.855471 8.089315 16.290809
C 12.233112 9.194062 17.477926
C 13.837272 1.989725 17.531630
C 6.413062 2.381633 12.794544
C 9.263016 2.738902 11.813806
H 6.982820 7.024803 9.997827
H 8.690581 6.877587 9.557200
H 7.809226 5.475739 10.192175
H 7.701079 9.491472 11.776386
H 8.862259 9.288512 13.102923
H 9.416608 9.261467 11.410732

H 5.933020 7.130312 12.709964
H 6.675871 5.584194 13.152910
H 6.966182 7.032423 14.146921
H 12.118702 6.390767 19.338300
H 13.732531 6.605437 21.202874
H 16.086838 5.865005 20.905249
H 16.807934 4.897945 18.732146
H 15.186696 4.664495 16.877655
H 12.605553 10.219632 17.371927
H 11.143017 9.214521 17.398996
H 12.512629 8.837893 18.473777
H 12.993601 9.827356 14.325673
H 12.987026 8.198434 13.628557
H 11.482065 8.901724 14.273363
H 15.226961 9.101897 16.092643
H 15.185419 7.798892 17.290959
H 15.326538 7.417491 15.566108
H 5.480458 4.754297 15.406776
H 3.319480 3.647306 15.898625
H 3.283179 1.326162 16.786856
H 5.421202 0.123707 17.182532
H 7.578761 1.233977 16.694085
H 6.107822 6.986494 18.779894
H 6.721323 7.335812 17.152117
H 5.483189 6.078218 17.397291
H 6.926843 4.297680 20.204196
H 6.178106 3.457083 18.837911
H 7.838460 3.071348 19.315423
H 8.827124 6.553314 19.938313
H 9.977645 5.403881 19.239521
H 9.610097 6.931977 18.395230
H 9.028778 3.752849 11.479562
H 10.334410 2.686298 12.019657
H 9.032025 2.043843 10.999118
H 13.801561 3.595370 14.783145
H 15.291719 3.799301 13.833781
H 14.498508 5.207214 14.567628
C 11.602244 7.577541 10.572561
C 11.936752 7.973721 9.279048
C 11.886477 7.057274 8.229106
C 11.501476 5.740588 8.478972
C 11.175018 5.338791 9.773386
H 11.648674 8.290673 11.390054
H 12.237951 9.000956 9.092431
H 12.143870 7.368498 7.220576
H 11.452183 5.022686 7.664800

H 10.861673 4.316845 9.964011
H 15.369300 5.271433 11.362960
H 13.971588 5.794849 10.408321
H 14.428284 6.675020 11.877410
H 14.207191 2.445761 11.414813
H 12.677429 2.089905 12.227415
H 12.680021 2.908299 10.654474
C 11.112053 1.453593 16.335382
H 11.083119 0.468197 16.813439
H 10.087137 1.789435 16.163822
H 11.593214 1.336628 15.358740
H 11.380570 1.720221 19.599059
H 11.859984 3.413048 19.793089
H 10.272920 3.012476 19.109744
H 13.766670 0.930832 17.808734
H 14.370400 2.043234 16.577327
H 14.436359 2.496537 18.290622
H 6.301849 1.771563 11.889971
H 5.726692 1.993435 13.550015
H 6.104053 3.400521 12.542987
H 8.432056 -0.130472 12.926115
H 9.667388 0.382069 14.086330
H 7.991877 0.148348 14.613792

ThNCOL3xyz

124
scf done : -2455.39870789
C 4.887396 2.268516 19.724406 -0.118405
C 4.917213 2.496693 21.105358 -0.033724
C 3.923662 3.291434 21.687720 -0.117785
C 2.916181 3.847581 20.901999 -0.128966
C 2.893583 3.620452 19.526280 -0.115224
C 3.882124 2.831534 18.939346 -0.133214
C 5.987145 1.868632 21.957079 0.395349
N 7.091957 2.536578 22.303836 -0.681634
Si 7.582513 4.183811 21.805673 10.542782
C 8.038941 4.244243 19.969068 -0.538945

Th 8.196333 0.422276 23.223175 62.164759
N 5.862275 0.608624 22.378504 -0.669940
Si 4.539156 -0.566577 22.108172 10.540491
C 2.800235 0.170333 22.282553 -0.532223
C 10.906849 1.175472 22.429791 0.394424
N 10.001099 0.759087 21.542950 -0.668281
Si 10.207136 0.239980 19.842823 10.541625
C 11.247185 1.427487 18.791989 -0.534645
C 12.357439 1.299182 22.047581 -0.033059
C 13.217979 0.204202 22.190386 -0.118045
C 14.560390 0.311913 21.829227 -0.133054
C 15.055064 1.511982 21.320313 -0.115210
C 14.201145 2.604668 21.172386 -0.129057
C 12.859284 2.499598 21.533305 -0.117277
N 10.483321 1.455132 23.666022 -0.678158
Si 11.446724 2.000000 25.070531 10.538765
C 12.435583 0.569745 25.820164 -0.538745
N 8.038659 -1.638302 22.122567 -0.498937
C 7.935853 -2.681262 21.528245 0.360905
O 7.834817 -3.710023 20.942291 -0.430580
N 8.620596 -1.086070 25.162460 -0.669574
C 7.798763 -0.442557 25.993609 0.394254
C 7.453706 -1.020962 27.338654 -0.032778
C 8.245062 -0.729382 28.455014 -0.115668
C 7.926549 -1.262296 29.701885 -0.127250
C 6.815490 -2.093037 29.846323 -0.116787
C 6.024723 -2.389868 28.736915 -0.132746
C 6.341486 -1.856985 27.487692 -0.118190
N 7.290663 0.725777 25.590251 -0.679613
Si 6.120708 1.778649 26.439496 10.535933
C 4.355386 1.137381 26.199000 -0.536295
C 10.974415 -1.483931 19.721084 -0.533261
C 8.463695 0.237317 19.113791 -0.545226
C 10.206258 2.622188 26.350632 -0.534150
C 12.627768 3.437809 24.710399 -0.531139
Si 9.390172 -2.693685 25.329919 10.541186
C 10.173094 -3.019074 27.026591 -0.532499
C 8.157383 -4.086197 24.984686 -0.531185
C 10.790369 -2.719739 24.061234 -0.544784
C 6.267094 3.480687 25.637961 -0.531406
C 6.430627 2.015276 28.293930 -0.527652
C 4.653152 -1.384242 20.405852 -0.531791
C 4.734768 -1.864879 23.467429 -0.543110
C 9.108650 4.608254 22.831543 -0.530946
C 6.302255 5.538785 22.155321 -0.533151
H 10.423100 -2.513835 23.052764 0.162295

H 11.273665 -3.702756 24.053215 0.136643
H 11.549070 -1.974114 24.318821 0.134744
H 10.827229 -2.203077 27.348175 0.129699
H 10.789152 -3.922901 26.945661 0.133662
H 9.428558 -3.187649 27.807851 0.149287
H 7.349340 -4.087959 25.722486 0.134485
H 8.668227 -5.053687 25.049391 0.136363
H 7.717433 -4.006678 23.987803 0.156035
H 9.111042 -0.083241 28.344598 0.135904
H 8.547548 -1.028520 30.562281 0.127756
H 6.567879 -2.508489 30.819106 0.126551
H 5.159484 -3.038693 28.841664 0.129393
H 5.727886 -2.092278 26.622617 0.142224
H 6.178736 1.132992 28.886017 0.149686
H 5.799549 2.845170 28.634510 0.133106
H 7.470508 2.284729 28.501666 0.133933
H 4.125808 1.029181 25.135341 0.149374
H 3.635083 1.839607 26.633907 0.132565
H 4.210277 0.167655 26.684397 0.135079
H 7.220661 3.951027 25.896912 0.139027
H 5.463001 4.134021 25.994544 0.128092
H 6.206932 3.412237 24.548996 0.155487
H 5.732607 -2.310549 23.457090 0.161655
H 4.003382 -2.668868 23.330391 0.136327
H 4.561377 -1.415885 24.450427 0.135247
H 2.684909 0.752782 23.201506 0.130710
H 2.085163 -0.660068 22.327240 0.133786
H 2.524351 0.806171 21.438201 0.149324
H 4.541379 -0.647448 19.605025 0.132490
H 3.841316 -2.113244 20.300677 0.136590
H 5.596649 -1.915803 20.260544 0.156914
H 3.938245 3.468641 22.759114 0.136620
H 2.146750 4.458366 21.366262 0.128120
H 2.108219 4.055466 18.914571 0.126651
H 3.870895 2.650326 17.868084 0.129688
H 5.652375 1.647816 19.266810 0.143555
H 5.452098 5.507143 21.470928 0.147009
H 6.797965 6.509703 22.035724 0.133851
H 5.924759 5.486546 23.181080 0.134337
H 8.833747 3.527976 19.742550 0.147521
H 8.399953 5.245101 19.706125 0.133365
H 7.179019 4.020354 19.331323 0.139676
H 8.848976 4.708051 23.889889 0.141891
H 9.527506 5.564196 22.497707 0.128370
H 9.880554 3.839879 22.747814 0.154873
H 7.782218 -0.383031 19.701810 0.162810

H 8.479997 -0.152787 18.090313 0.136819
H 8.067956 1.257182 19.078713 0.135809
H 10.906530 2.463944 18.878948 0.130505
H 11.140865 1.133491 17.740801 0.134171
H 12.309044 1.393990 19.045276 0.149090
H 11.994217 -1.492213 20.116964 0.135794
H 11.022730 -1.789676 18.669749 0.136494
H 10.385991 -2.231013 20.259052 0.156932
H 12.196896 3.352269 21.416435 0.135896
H 14.579507 3.542113 20.774089 0.128185
H 16.100865 1.594875 21.038119 0.126673
H 15.218860 -0.544734 21.943950 0.129751
H 12.831781 -0.733531 22.579814 0.144586
H 13.504874 3.136001 24.134604 0.148916
H 12.972787 3.841388 25.670015 0.133632
H 12.123364 4.247683 24.174474 0.134902
H 11.775171 -0.261263 26.083391 0.148679
H 12.944049 0.901479 26.732617 0.132528
H 13.198026 0.200381 25.128227 0.137934
H 9.753802 3.564347 26.025982 0.139346
H 10.714028 2.806876 27.303910 0.128347
H 9.399719 1.903792 26.516732 0.155731
ThNCSL3xyz

124
scf done : -2390.39780427
Th 12.383750 6.515604 5.963759 62.174156
N 11.746291 7.986880 4.070641 -0.678262
C 10.601882 8.414911 4.609812 0.411031
C 9.810004 9.506639 3.947667 -0.033744
C 10.257168 10.832062 3.988770 -0.120807
C 9.522323 11.840487 3.368732 -0.129885
C 8.340872 11.533461 2.693508 -0.116941
C 7.893673 10.213428 2.645605 -0.129493
C 8.621612 9.204452 3.273746 -0.119568
N 12.754613 7.549701 8.170005 -0.685575
Si 13.362015 9.146143 8.712817 10.537891
C 15.225304 9.143271 9.024456 -0.528917
N 14.494025 7.604380 5.537789 -0.460340
C 15.543482 8.132615 5.344201 -0.129502
S 16.984939 8.858105 5.081037 9.896721
N 11.859792 5.442243 8.224421 -0.679085
Si 11.365882 3.859819 8.900480 10.542538
N 13.803521 4.540532 5.470110 -0.674473
C 12.938114 3.987813 4.618370 0.399117
C 13.355506 2.853698 3.719228 -0.035309

C 13.159077 1.522223 4.101948 -0.115890
C 13.560194 0.482060 3.265170 -0.131380
C 14.158029 0.759852 2.036577 -0.113441
C 14.357596 2.084235 1.649861 -0.135012
C 13.962348 3.126573 2.487104 -0.113856
N 11.704341 4.502490 4.570939 -0.680142
Si 10.324689 3.994072 3.554251 10.540287
N 10.191425 7.848623 5.750503 -0.684819
Si 8.792410 8.347959 6.755319 10.541940
C 9.126476 7.699586 8.494649 -0.532777
Si 12.552040 8.504516 2.552802 10.539190
C 13.584791 10.069762 2.790745 -0.528066
C 12.397500 6.475616 8.880709 0.412045
C 12.622167 6.435298 10.365666 -0.032305
C 12.478753 9.787223 10.262590 -0.533209
C 12.969323 10.323771 7.287916 -0.552315
Si 15.494704 4.085329 5.853631 10.541414
C 16.689050 4.590604 4.481518 -0.532955
C 15.700325 2.235023 6.212457 -0.534130
C 15.904860 5.001224 7.454557 -0.548763
C 10.517945 4.594343 1.770667 -0.538030
C 9.986858 2.128947 3.531985 -0.531831
C 8.812642 4.808514 4.334904 -0.538963
C 11.349157 8.781510 1.113106 -0.532007
C 13.692014 7.074703 2.079918 -0.544516
C 7.161311 7.588284 6.158860 -0.527684
C 8.551002 10.223413 6.896242 -0.532739
H 13.495752 10.031435 6.375100 0.170834
H 13.281269 11.342229 7.543614 0.137895
H 11.894409 10.341145 7.082258 0.142717
H 15.494119 8.532092 9.890382 0.129751
H 15.557661 10.168413 9.224976 0.140232
H 15.778191 8.782303 8.153051 0.169542
H 11.389697 9.759061 10.158927 0.132388
H 12.769092 10.833695 10.414184 0.135964
H 12.750997 9.229843 11.161960 0.150357
H 15.788523 6.082260 7.345280 0.170229
H 16.943204 4.802502 7.741696 0.139176
H 15.260138 4.657328 8.270157 0.137729
H 16.494322 4.035361 3.559498 0.136152
H 17.718391 4.377451 4.791431 0.139135
H 16.621489 5.660834 4.268328 0.168212
H 14.964921 1.878050 6.940595 0.129647
H 16.695258 2.079093 6.646314 0.136117
H 15.624097 1.617331 5.315124 0.148385
H 12.690249 1.301423 5.055808 0.134433

H 13.403769 -0.547551 3.575126 0.128393
H 14.468924 -0.051806 1.384845 0.127117
H 14.825827 2.309674 0.695672 0.130682
H 14.129533 4.156757 2.187646 0.143447
H 14.278609 7.342249 1.194273 0.139989
H 13.105311 6.183366 1.839149 0.134632
H 12.960689 10.956352 2.931375 0.127764
H 14.196013 10.233888 1.895782 0.140947
H 14.265173 9.979541 3.641255 0.168785
H 10.658168 7.943623 0.981169 0.132535
H 11.935576 8.874715 0.191172 0.135212
H 10.759093 9.693662 1.230019 0.149524
H 8.278276 8.175111 3.226901 0.137076
H 6.977937 9.967283 2.115128 0.129201
H 7.771284 12.319962 2.206528 0.128375
H 9.872180 12.868061 3.415155 0.131693
H 11.170997 11.072544 4.523689 0.146730
H 8.258322 7.895713 9.133599 0.129036
H 9.993885 8.203238 8.929246 0.147139
H 9.321385 6.624624 8.498055 0.151633
H 8.586266 4.353983 5.305738 0.137977
H 7.933840 4.676812 3.694116 0.132183
H 8.967663 5.878231 4.495462 0.158995
H 11.373462 4.120777 1.280039 0.141022
H 10.656318 5.678374 1.735439 0.149016
H 9.620880 4.346981 1.191754 0.134972
C 11.544165 6.543308 11.251347 -0.119778
C 11.760133 6.510287 12.627928 -0.131236
C 13.051643 6.359137 13.131936 -0.116443
C 14.129155 6.250564 12.253011 -0.130254
C 13.917756 6.295728 10.876458 -0.119105
H 10.540264 6.674466 10.859646 0.136620
H 10.917746 6.605607 13.307603 0.128694
H 13.218139 6.328565 14.205040 0.127960
H 15.137516 6.129794 12.639162 0.131413
H 14.756043 6.207384 10.191880 0.146234
C 9.591893 3.923474 9.568272 -0.528255
C 11.386959 2.625782 7.474236 -0.533385
C 12.512891 3.171360 10.242825 -0.530860
H 11.006602 1.657577 7.818065 0.129658
H 10.772360 2.963257 6.636527 0.155516
H 12.408396 2.486661 7.109907 0.145180
H 12.234338 2.126768 10.426876 0.133638
H 13.558684 3.182233 9.920500 0.137883
H 12.442423 3.711985 11.188999 0.151614
H 9.288437 2.915952 9.875925 0.136287

H 9.491843 4.578675 10.437537 0.132286
H 8.887427 4.260533 8.801314 0.137443
H 7.819314 10.410985 7.691367 0.133599
H 9.476678 10.737028 7.173075 0.136594
H 8.172438 10.674330 5.976171 0.151293
H 6.376042 7.829505 6.884857 0.135217
H 6.849105 7.987683 5.190288 0.131569
H 7.222243 6.499804 6.079569 0.140921
H 14.392477 6.834615 2.884709 0.169027
H 8.989458 1.973627 3.103103 0.134680
H 10.705337 1.572538 2.926797 0.150431
H 9.982642 1.703252 4.539991 0.132867
ThOCNL3xyz

124
scf done : -2455.37737947
C 13.185837 0.151263 22.123075 -0.118393
C 12.353060 1.274883 22.060995 -0.033164
C 12.878830 2.491203 21.612287 -0.116859
C 14.216020 2.582160 21.230847 -0.129432
C 15.042585 1.461004 21.298940 -0.114704
C 14.524992 0.246387 21.747179 -0.132823
C 10.902680 1.162624 22.445257 0.396315
N 10.468143 1.500013 23.665050 -0.684808
Th 8.194494 0.459264 23.244061 62.181879
O 8.019812 -1.558781 22.182964 -0.557644
C 7.935855 -2.654970 21.577035 0.350100
N 7.857413 -3.681829 21.010642 -0.422316
N 10.005042 0.697220 21.574543 -0.671950
Si 10.214164 0.171396 19.872142 10.534430
C 10.928911 -1.571861 19.746095 -0.536050
Si 11.433363 2.056991 25.066544 10.540108
C 10.188153 2.617957 26.369861 -0.534653
C 8.475308 0.205708 19.132939 -0.546908
C 11.289757 1.351551 18.850841 -0.533455
C 12.556101 3.544538 24.722728 -0.532432
C 12.481903 0.647270 25.770991 -0.540349
N 8.596381 -1.078936 25.147677 -0.672038
Si 9.379296 -2.685596 25.304125 10.535324
C 8.161342 -4.087609 24.957031 -0.534956
C 7.784833 -0.435911 25.988939 0.395261
N 7.288175 0.743143 25.597764 -0.684536
Si 6.116870 1.791138 26.454490 10.535797
C 6.253540 3.493217 25.651105 -0.532620
C 7.438955 -1.025590 27.328566 -0.033236
C 8.220197 -0.730507 28.451135 -0.115625

C 7.903945 -1.278513 29.692026 -0.127489
C 6.804370 -2.126318 29.824410 -0.116245
C 6.023396 -2.425915 28.708916 -0.132504
C 6.338846 -1.879625 27.465183 -0.117727
C 10.765909 -2.690702 24.020737 -0.547059
C 10.180925 -2.989553 26.995041 -0.532935
C 6.437921 2.028384 28.306304 -0.527956
C 4.354749 1.139295 26.222438 -0.537010
N 5.886367 0.620973 22.365081 -0.673695
Si 4.567809 -0.565630 22.093955 10.534429
C 4.705142 -1.400546 20.404826 -0.534688
C 6.001476 1.886211 21.957489 0.397355
N 7.100662 2.560315 22.316718 -0.687571
Si 7.583395 4.210371 21.812462 10.542677
C 9.121164 4.635590 22.819682 -0.530293
C 4.927564 2.512369 21.110496 -0.033767
C 3.941141 3.313584 21.696017 -0.117933
C 2.925721 3.862668 20.915619 -0.128946
C 2.889080 3.622873 19.542379 -0.114783
C 3.870975 2.827893 18.952428 -0.132754
C 4.883398 2.270613 19.732256 -0.118827
C 4.771014 -1.851309 23.463688 -0.545180
C 2.829771 0.173049 22.260211 -0.532259
C 6.307364 5.563890 22.180462 -0.533362
C 8.013726 4.267669 19.969582 -0.539399
H 10.386500 -2.546259 23.006078 0.165740
H 11.290906 -3.651697 24.044960 0.140512
H 11.494062 -1.904164 24.242539 0.134602
H 10.823224 -2.160318 27.306794 0.129882
H 10.811272 -3.883304 26.914022 0.135380
H 9.445111 -3.166644 27.782630 0.150193
H 7.315661 -4.062584 25.650629 0.132136
H 8.671527 -5.048523 25.090825 0.138457
H 7.780145 -4.059243 23.932521 0.169301
H 9.077375 -0.071201 28.350537 0.135853
H 8.517860 -1.043140 30.557021 0.128639
H 6.558430 -2.553324 30.792569 0.127744
H 5.167457 -3.088301 28.804103 0.131021
H 5.733850 -2.119305 26.595277 0.143200
H 6.191279 1.145426 28.899461 0.150685
H 5.807536 2.857027 28.651070 0.134223
H 7.478645 2.299651 28.507332 0.134445
H 4.117296 1.030476 25.160511 0.149810
H 3.633101 1.836498 26.663127 0.133917
H 4.218722 0.168238 26.707732 0.136925
H 7.204489 3.969345 25.909115 0.139306

H 5.446398 4.142037 26.008960 0.129680
H 6.192202 3.425751 24.562138 0.155366
H 5.742300 -2.349734 23.413984 0.165742
H 3.997987 -2.621762 23.370378 0.139623
H 4.663998 -1.383947 24.447538 0.134488
H 2.712047 0.762087 23.174634 0.130805
H 2.114518 -0.656893 22.307914 0.135459
H 2.556985 0.802970 21.410401 0.150040
H 4.633786 -0.674487 19.589845 0.129670
H 3.876310 -2.108870 20.290083 0.138765
H 5.634913 -1.965884 20.295904 0.170126
H 3.966368 3.500236 22.765608 0.136913
H 2.160951 4.477477 21.382192 0.129035
H 2.097512 4.052410 18.934867 0.127957
H 3.848489 2.636657 17.883156 0.131422
H 5.642376 1.644126 19.272606 0.144899
H 5.449106 5.533743 21.506309 0.147682
H 6.801824 6.534889 22.056635 0.134917
H 5.942564 5.509911 23.210709 0.134895
H 8.807195 3.554401 19.728437 0.147940
H 8.367148 5.269582 19.700449 0.134824
H 7.144479 4.041176 19.345594 0.141243
H 8.876952 4.722250 23.882946 0.142468
H 9.526466 5.598538 22.489366 0.129869
H 9.897856 3.874741 22.716200 0.154251
H 7.790110 -0.454658 19.670984 0.165546
H 8.505486 -0.127976 18.090039 0.140109
H 8.071553 1.222922 19.149337 0.135537
H 10.984923 2.395665 18.970983 0.130902
H 11.172179 1.092095 17.791967 0.135557
H 12.350474 1.274989 19.100389 0.150457
H 11.926834 -1.631414 20.189655 0.130998
H 11.019615 -1.843463 18.687959 0.138637
H 10.283777 -2.316429 20.220805 0.170670
H 12.237438 3.365800 21.556235 0.135796
H 14.611728 3.530654 20.878266 0.129111
H 16.084990 1.533111 21.001718 0.127988
H 15.162645 -0.631597 21.801934 0.131589
H 12.781138 -0.797718 22.462895 0.145976
H 13.442436 3.285779 24.140337 0.148152
H 12.889333 3.945627 25.687526 0.135026
H 12.018181 4.342973 24.202712 0.135525
H 11.857319 -0.214185 26.024041 0.149102
H 12.991778 0.976471 26.683482 0.133840
H 13.246156 0.322410 25.059078 0.141764
H 9.699224 3.547688 26.062585 0.139972

H 10.703448 2.810212 27.317547 0.130143
H 9.409015 1.871271 26.539876 0.155530
ThOCPL3xyz

124
scf done : -2407.17391454
Th 10.280992 5.402948 15.037350 62.168695
N 8.429389 4.676494 16.508894 -0.679832
Si 7.755713 5.264762 18.064167 10.536697
C 7.208947 3.847624 19.197762 -0.535207
N 11.735259 5.112399 12.952972 -0.679891
Si 13.366667 4.537161 12.489857 10.538944
C 14.350533 4.334547 14.084704 -0.532851
N 9.751195 6.242582 12.783338 -0.687419
Si 8.355156 7.122086 12.079731 10.537613
C 8.628275 8.990366 12.036550 -0.529739
N 12.205949 6.459606 16.173206 -0.676046
C 12.542078 5.380975 16.882589 0.408566
C 13.544109 5.483979 18.000689 -0.036168
C 13.178222 6.070751 19.218672 -0.119106
C 14.106637 6.185371 20.251978 -0.133520
C 15.412807 5.730276 20.075021 -0.113882
C 15.785246 5.154608 18.861080 -0.131645
C 14.855468 5.026467 17.830320 -0.117840
N 11.937237 4.225406 16.578052 -0.688613
Si 12.098106 2.650916 17.414169 10.548497
C 11.539008 2.731607 19.220724 -0.542792
N 8.679130 3.449006 14.590981 -0.684115
Si 8.231760 2.297035 13.290787 10.539719
C 8.542365 0.492318 13.780968 -0.527395
O 9.354806 7.466753 15.574007 -0.513127
C 8.848602 8.568830 15.848070 -0.129938
P 8.192741 9.989421 16.198902 9.981253
C 7.956681 3.743891 15.677875 0.409134
C 6.642422 3.069503 15.957935 -0.037590
C 5.439702 3.749742 15.733874 -0.120107
C 4.220623 3.128676 15.998490 -0.130126
C 4.190363 1.827498 16.499579 -0.115960
C 5.385631 1.147977 16.731900 -0.130488
C 6.606244 1.763642 16.459316 -0.118785
C 9.187967 6.169488 18.899192 -0.549791
C 6.327309 6.477692 17.830542 -0.531455
C 10.898736 5.855130 12.219931 0.413253
C 11.235023 6.268585 10.814764 -0.030713
C 6.903726 6.736974 13.226421 -0.552660
C 7.904462 6.510674 10.342999 -0.534791

C 13.262677 2.837889 11.655388 -0.527387
C 14.362432 5.717121 11.390549 -0.528382
Si 12.891791 8.117027 16.184895 10.536573
C 12.415021 8.844472 14.507352 -0.551461
C 14.782942 8.128805 16.308708 -0.532969
C 12.171936 9.177077 17.570224 -0.530963
C 13.839342 1.898965 17.359348 -0.533600
C 6.438972 2.448363 12.693281 -0.535255
C 9.343365 2.696593 11.821320 -0.532947
H 6.946228 6.960331 10.056797 0.135957
H 8.645255 6.793281 9.591336 0.149954
H 7.780734 5.423739 10.311339 0.132809
H 7.689682 9.482862 11.757151 0.139315
H 8.916861 9.372417 13.020223 0.165877
H 9.386734 9.280086 11.304425 0.127502
H 5.988681 7.193278 12.833361 0.137557
H 6.738341 5.657496 13.297404 0.140298
H 7.067487 7.137466 14.230794 0.173268
H 12.163964 6.433210 19.356371 0.146048
H 13.808554 6.635135 21.195020 0.131419
H 16.137575 5.826411 20.878569 0.127711
H 16.803685 4.806152 18.712667 0.128894
H 15.150637 4.586618 16.882987 0.138178
H 12.534786 10.205999 17.464474 0.138457
H 11.079474 9.208329 17.522547 0.166754
H 12.475978 8.816265 18.556710 0.129726
H 12.831746 9.851983 14.402175 0.138286
H 12.810720 8.228935 13.692834 0.141065
H 11.329718 8.921781 14.396718 0.171982
H 15.132890 9.153293 16.134646 0.136190
H 15.139828 7.813837 17.292001 0.150310
H 15.249184 7.488480 15.553406 0.130765
H 5.461523 4.760652 15.338003 0.149310
H 3.293161 3.662895 15.811800 0.132309
H 3.239534 1.345933 16.709716 0.128406
H 5.369534 0.137165 17.130101 0.129260
H 7.535066 1.235496 16.653529 0.136668
H 6.081343 6.928483 18.799117 0.138302
H 6.605181 7.290347 17.152671 0.167242
H 5.425232 5.989995 17.452428 0.129493
H 7.026327 4.251781 20.200342 0.135218
H 6.287767 3.370663 18.854230 0.150595
H 7.979800 3.075811 19.285749 0.133053
H 8.868580 6.555161 19.873446 0.138555
H 10.031567 5.493018 19.065501 0.137283
H 9.526654 7.020996 18.301844 0.171477

H 9.110578 3.690681 11.430937 0.146366
H 10.402647 2.673034 12.087510 0.150752
H 9.172906 1.966133 11.022706 0.129637
H 13.819156 3.707911 14.803895 0.153664
H 15.321292 3.875765 13.866720 0.129283
H 14.527047 5.308412 14.549850 0.146461
C 11.577153 7.597194 10.538019 -0.120224
C 11.897439 7.984190 9.238268 -0.129228
C 11.865213 7.052407 8.201049 -0.116207
C 11.516312 5.729257 8.470222 -0.131711
C 11.209078 5.336144 9.771629 -0.120411
H 11.607504 8.322120 11.346054 0.147843
H 12.173539 9.015384 9.036070 0.131461
H 12.109913 7.356395 7.187177 0.128028
H 11.482073 5.000151 7.665312 0.128673
H 10.930717 4.307541 9.978599 0.135725
H 15.397849 5.356552 11.363091 0.133576
H 13.995251 5.771570 10.363784 0.150610
H 14.377736 6.731138 11.801775 0.137429
H 14.275072 2.465280 11.461140 0.136137
H 12.759633 2.109048 12.298934 0.137263
H 12.732396 2.873388 10.699854 0.132600
C 10.984453 1.466909 16.458279 -0.533321
H 10.921686 0.504276 16.977352 0.132221
H 9.974639 1.864708 16.337639 0.157849
H 11.391499 1.280764 15.458986 0.141451
H 11.577228 1.728391 19.660580 0.135375
H 12.184524 3.383064 19.816273 0.146730
H 10.511272 3.095927 19.305005 0.146894
H 13.759787 0.840954 17.637077 0.136084
H 14.275926 1.942765 16.356941 0.133247
H 14.528647 2.377288 18.058226 0.144623
H 6.330919 1.851097 11.779889 0.133648
H 5.715880 2.080269 13.424459 0.151563
H 6.178059 3.481222 12.443468 0.138258
H 8.452436 -0.135874 12.887057 0.135165
H 9.543564 0.346934 14.194907 0.138787
H 7.812562 0.133934 14.511403 0.133748
ThPCOL3xyz

124
scf done : -2407.16159701
Th 10.222287 5.540014 15.072552 61.770569
N 8.396221 4.752284 16.526210 -0.687789
Si 7.740560 5.173706 18.151844 10.543688
C 7.346799 3.643928 19.204393 -0.535245

N 11.682625 5.152049 13.021065 -0.676464
Si 13.302762 4.523992 12.576314 10.541461
C 14.253970 4.225148 14.174771 -0.527087
N 9.711692 6.298589 12.787812 -0.684324
Si 8.338461 7.140067 11.989336 10.543793
C 8.524978 9.016690 12.076204 -0.530641
N 12.251902 6.516744 16.097599 -0.679412
C 12.500042 5.457763 16.872840 0.410507
C 13.500165 5.546010 17.991514 -0.037979
C 13.188806 6.274724 19.145591 -0.119656
C 14.099921 6.347272 20.197435 -0.130093
C 15.335086 5.706690 20.101154 -0.115995
C 15.655561 4.991545 18.947642 -0.131454
C 14.741367 4.906701 17.898896 -0.121266
N 11.803527 4.336328 16.646039 -0.683762
Si 11.925661 2.788477 17.547710 10.544167
C 12.042344 2.967472 19.430332 -0.534769
N 8.701689 3.557168 14.591898 -0.673404
Si 8.328847 2.368539 13.299199 10.539743
C 8.989720 0.650557 13.755840 -0.525021
P 9.395801 8.305055 15.959026 10.039449
C 7.763435 8.556042 15.722933 -0.009569
O 6.618890 8.808387 15.593080 -0.358964
C 7.957155 3.820828 15.672095 0.410549
C 6.646681 3.123110 15.909698 -0.035365
C 5.443590 3.803135 15.688559 -0.117567
C 4.225149 3.156641 15.887293 -0.129586
C 4.195948 1.832117 16.322521 -0.115255
C 5.392129 1.153906 16.555748 -0.131817
C 6.612524 1.793563 16.344966 -0.122846
C 9.119326 6.102548 19.047812 -0.544169
C 6.178616 6.235360 18.085981 -0.535258
C 10.875509 5.903695 12.263675 0.404715
C 11.265276 6.331088 10.876020 -0.030749
C 6.807275 6.555893 12.928708 -0.546658
C 8.079543 6.647759 10.175265 -0.530062
C 13.152926 2.849917 11.697022 -0.527470
C 14.368861 5.686489 11.525192 -0.530927
Si 13.157126 8.069930 15.999466 10.551641
C 12.724812 8.772091 14.300248 -0.546607
C 15.038870 7.840554 16.057514 -0.533936
C 12.670935 9.294686 17.353195 -0.534499
C 13.379843 1.727884 16.945617 -0.525342
C 6.499618 2.214908 12.826062 -0.530088
C 9.244673 2.915335 11.746030 -0.528940
H 7.120628 7.072174 9.853520 0.135770

H 8.857025 7.035732 9.513490 0.149541
H 8.020813 5.564063 10.036841 0.130646
H 7.573403 9.497094 11.822925 0.138655
H 8.812618 9.337433 13.080922 0.168969
H 9.277549 9.369926 11.364949 0.128749
H 5.906239 6.987207 12.479201 0.138867
H 6.726405 5.466239 12.870216 0.142507
H 6.818294 6.851856 13.979928 0.166872
H 12.225800 6.770658 19.222058 0.154213
H 13.843353 6.905005 21.093845 0.131889
H 16.046288 5.767524 20.920149 0.127914
H 16.621035 4.500567 18.861273 0.128745
H 14.997460 4.363873 16.994750 0.136566
H 13.100140 10.276130 17.120528 0.134074
H 11.585002 9.405864 17.413126 0.179085
H 13.052225 8.985503 18.330198 0.130900
H 13.244064 9.723941 14.143989 0.134053
H 13.037513 8.080807 13.511401 0.140530
H 11.649564 8.952993 14.208251 0.177866
H 15.499065 8.810486 15.832884 0.135965
H 15.395904 7.520992 17.039118 0.149815
H 15.399650 7.127951 15.309846 0.131447
H 5.464244 4.832957 15.344699 0.154754
H 3.297094 3.689915 15.700893 0.132599
H 3.245120 1.331157 16.481311 0.127842
H 5.376678 0.124742 16.903916 0.128476
H 7.542849 1.268657 16.538061 0.136036
H 5.998924 6.653579 19.083447 0.135874
H 6.259706 7.068876 17.385384 0.167881
H 5.304430 5.636872 17.814969 0.134514
H 7.055855 3.997251 20.201207 0.135109
H 6.519470 3.052088 18.807137 0.147452
H 8.212632 2.987457 19.333481 0.131372
H 8.839346 6.243372 20.097947 0.130816
H 10.050188 5.527304 19.022684 0.137280
H 9.301370 7.084551 18.603070 0.178755
H 8.832168 3.849968 11.358887 0.146954
H 10.308389 3.063431 11.942945 0.149384
H 9.137802 2.145957 10.973193 0.129249
H 13.692015 3.595889 14.866951 0.150589
H 15.204295 3.729871 13.946547 0.128352
H 14.472407 5.171142 14.675684 0.143355
C 11.619633 7.663302 10.632585 -0.119140
C 11.988631 8.069442 9.351790 -0.130492
C 11.992106 7.154215 8.299320 -0.116421
C 11.626871 5.829036 8.534275 -0.131264

C 11.269999 5.416883 9.816921 -0.118355
H 11.618671 8.376069 11.451752 0.149092
H 12.273939 9.103020 9.176208 0.130987
H 12.274981 7.473118 7.300043 0.127733
H 11.615848 5.113654 7.716497 0.128275
H 10.969693 4.389104 9.993539 0.133895
H 15.383977 5.272362 11.494627 0.133260
H 14.015175 5.793052 10.497933 0.150576
H 14.434536 6.684246 11.969607 0.138721
H 14.155290 2.428208 11.558782 0.136288
H 12.573406 2.136994 12.292147 0.138572
H 12.688940 2.929479 10.710616 0.131386
C 10.361793 1.810669 17.164671 -0.528961
H 10.446443 0.804523 17.589852 0.128987
H 9.483764 2.295147 17.596845 0.144548
H 10.204011 1.717731 16.088125 0.151803
H 11.899574 1.974860 19.874280 0.133587
H 13.007839 3.351366 19.766774 0.151516
H 11.258053 3.620653 19.823974 0.141617
H 13.333524 0.759595 17.458050 0.136424
H 13.321524 1.532869 15.870366 0.136987
H 14.355216 2.169335 17.163589 0.131729
H 6.443877 1.630274 11.899758 0.133371
H 5.897634 1.709013 13.583780 0.150531
H 6.046522 3.190688 12.627623 0.139211
H 8.827268 -0.028698 12.910863 0.136422
H 10.065508 0.675200 13.956278 0.136237
H 8.488593 0.220335 14.626796 0.132346
ThSCNL3xyz

124
scf done :-2390.36997049
Th 12.457816 6.589876 5.948995 61.870038
N 11.782988 8.038351 4.077193 -0.691815
C 10.644781 8.447554 4.648990 0.414182
C 9.850245 9.573141 4.051860 -0.034970
C 10.343666 10.881651 4.108724 -0.120550
C 9.602778 11.933460 3.573167 -0.130041
C 8.373534 11.686647 2.962471 -0.115306
C 7.884122 10.382463 2.892343 -0.131896
C 8.614963 9.330032 3.440513 -0.123453
N 12.767268 7.572342 8.185222 -0.689542
Si 13.274744 9.176807 8.829917 10.539535
C 15.146069 9.263416 9.056438 -0.525784
S 15.136872 7.665552 5.396814 9.973188
C 15.317506 9.364870 5.365415 -0.052536

N 15.489030 10.522655 5.328412 -0.422154
N 11.937034 5.433725 8.155308 -0.678755
Si 11.460547 3.826658 8.793761 10.540125
N 13.908246 4.643249 5.427969 -0.684286
C 13.033275 4.085082 4.584962 0.412884
C 13.439543 2.930109 3.714126 -0.038688
C 12.948310 1.641857 3.953137 -0.119762
C 13.329953 0.581061 3.133639 -0.131865
C 14.194907 0.799292 2.061675 -0.115808
C 14.686741 2.081630 1.819193 -0.130363
C 14.318766 3.141493 2.645569 -0.119281
N 11.801226 4.607611 4.517262 -0.687611
Si 10.461239 4.097948 3.435435 10.542091
N 10.259165 7.842698 5.780064 -0.680902
Si 8.827732 8.233358 6.790542 10.540558
C 9.203593 7.634664 8.537340 -0.531096
Si 12.431877 8.455747 2.444467 10.544070
C 13.318332 10.119628 2.393570 -0.534922
C 12.436381 6.462740 8.852167 0.403817
C 12.651802 6.374427 10.336237 -0.030864
C 12.438437 9.638253 10.468815 -0.530257
C 12.689000 10.425361 7.541796 -0.552411
Si 15.530228 4.026853 5.917128 10.550075
C 16.898831 4.517936 4.710208 -0.534719
C 15.579575 2.144080 6.140193 -0.531860
C 15.863114 4.801742 7.607494 -0.551476
C 10.985284 3.647235 1.671458 -0.533300
C 9.473167 2.652203 4.166679 -0.525960
C 9.270485 5.552898 3.309368 -0.529640
C 11.088377 8.464291 1.104461 -0.534402
C 13.639745 7.068547 2.013810 -0.549721
C 7.298717 7.281012 6.196564 -0.525794
C 8.397120 10.074551 6.911868 -0.531687
H 13.240433 10.349962 6.601143 0.180107
H 12.847171 11.441212 7.920496 0.140924
H 11.619425 10.301281 7.346960 0.139614
H 15.473284 8.660649 9.908678 0.126723
H 15.444767 10.301006 9.241687 0.144957
H 15.668343 8.921123 8.159758 0.177532
H 11.348732 9.550785 10.422669 0.130480
H 12.674608 10.689958 10.671819 0.137604
H 12.794656 9.044917 11.313696 0.149251
H 15.901419 5.893036 7.547152 0.183701
H 16.826998 4.450192 7.992136 0.136085
H 15.088665 4.512234 8.324558 0.141667
H 16.824190 3.972395 3.765560 0.131646

H 17.869718 4.277369 5.159131 0.136785
H 16.877504 5.591268 4.505021 0.188784
H 14.786409 1.777838 6.798787 0.131847
H 16.537870 1.891817 6.610272 0.136797
H 15.518004 1.602719 5.193617 0.151010
H 12.286924 1.468530 4.795820 0.136194
H 12.952412 -0.417701 3.334854 0.129616
H 14.486744 -0.026952 1.419521 0.129117
H 15.358637 2.258806 0.984002 0.132906
H 14.697826 4.140646 2.453767 0.154682
H 13.985996 7.193344 0.981663 0.133307
H 13.153083 6.091114 2.089578 0.138617
H 12.608331 10.951334 2.395794 0.132916
H 13.896354 10.179983 1.463768 0.136025
H 14.011475 10.251129 3.228110 0.185092
H 10.533490 7.522918 1.051611 0.130766
H 11.588407 8.602771 0.138091 0.136593
H 10.373572 9.280532 1.229260 0.149515
H 8.240140 8.313447 3.374949 0.135569
H 6.931917 10.182781 2.408565 0.128677
H 7.800307 12.507261 2.540298 0.128694
H 9.988189 12.947385 3.633600 0.135178
H 11.299538 11.074800 4.586458 0.161452
H 8.317367 7.772142 9.166810 0.130637
H 10.027815 8.202613 8.975545 0.149343
H 9.473356 6.576324 8.547482 0.149955
H 8.942846 5.896021 4.292885 0.151463
H 8.386474 5.247833 2.738460 0.130611
H 9.738330 6.394073 2.793318 0.145751
H 11.555174 2.717405 1.616191 0.151672
H 11.578303 4.441401 1.208187 0.141548
H 10.075315 3.523530 1.071873 0.134723
C 11.567328 6.401487 11.219865 -0.119120
C 11.779939 6.323150 12.594964 -0.130991
C 13.075110 6.206504 13.098973 -0.116411
C 14.159127 6.178809 12.221677 -0.130261
C 13.950622 6.269955 10.847060 -0.118803
H 10.560252 6.508543 10.829535 0.134920
H 10.932146 6.357031 13.273716 0.129035
H 13.239303 6.140982 14.170859 0.128678
H 15.170479 6.086217 12.607729 0.132036
H 14.794118 6.245066 10.163758 0.149045
C 9.656712 3.842980 9.379345 -0.527534
C 11.574385 2.604572 7.364608 -0.528445
C 12.557977 3.137933 10.177326 -0.530794
H 11.171787 1.635327 7.679224 0.130076

H 11.013542 2.947142 6.493210 0.150603
H 12.615912 2.462700 7.065892 0.144159
H 12.284107 2.086350 10.326485 0.134170
H 13.617126 3.165191 9.903830 0.138951
H 12.438731 3.657055 11.130283 0.151460
H 9.358623 2.821833 9.644151 0.137130
H 9.503672 4.469843 10.261781 0.132407
H 8.981597 4.189815 8.590590 0.137778
H 7.644780 10.189915 7.701558 0.134432
H 9.263946 10.679749 7.192917 0.142119
H 7.982939 10.482640 5.987600 0.151869
H 6.475505 7.463770 6.897093 0.137702
H 6.962668 7.592389 5.204172 0.132445
H 7.479040 6.201593 6.175092 0.136263
H 14.512236 7.079710 2.672629 0.185807
H 8.611396 2.461603 3.516152 0.137709
H 10.048400 1.725305 4.230246 0.132453
H 9.086353 2.887325 5.163032 0.137607
UClL3xyz

122
scf done: -1932.116298
U 10.238875 5.513642 15.063936
N 8.455444 4.696941 16.547031
Si 7.760939 5.288098 18.095359
C 7.114700 3.888674 19.201108
N 11.684670 5.088699 12.981678
Si 13.302884 4.475191 12.518420
C 14.298686 4.244616 14.101918
N 9.709226 6.221930 12.780673
Si 8.336187 7.119693 12.051790
C 8.613930 8.988274 12.086005
N 12.232731 6.487336 16.125658
C 12.546067 5.404282 16.843327
C 13.540180 5.498613 17.969078
C 13.151763 6.050025 19.196476
C 14.068121 6.162663 20.240742
C 15.384960 5.738949 20.066537
C 15.779813 5.197692 18.843926
C 14.862572 5.073548 17.801923
N 11.917097 4.266255 16.539601
Si 12.060046 2.680637 17.357579
C 11.273157 2.706323 19.078698
N 8.706452 3.465993 14.634424
Si 8.242986 2.314091 13.337275
C 8.622660 0.511426 13.785584

Cl 9.175090 7.946431 15.700998
C 7.990742 3.750169 15.723398
C 6.693609 3.054344 16.021462
C 5.475204 3.708700 15.807798
C 4.272305 3.063970 16.087945
C 4.274401 1.763487 16.592162
C 5.485699 1.108609 16.811859
C 6.689927 1.749017 16.525113
C 9.189241 6.127713 19.002529
C 6.379483 6.551996 17.835752
C 10.865327 5.829379 12.232305
C 11.220599 6.242541 10.831456
C 6.829132 6.694223 13.110870
C 7.950439 6.576038 10.275769
C 13.170422 2.782208 11.675616
C 14.331229 5.629402 11.418424
Si 12.960401 8.128380 16.140393
C 12.560294 8.858836 14.443447
C 14.848691 8.102772 16.309670
C 12.238101 9.217790 17.504015
C 13.823878 2.002252 17.529403
C 6.423348 2.405300 12.809145
C 9.274794 2.754574 11.824910
H 6.991989 7.022775 9.984014
H 8.705030 6.904761 9.558008
H 7.845598 5.490293 10.190984
H 7.694746 9.499133 11.778245
H 8.863314 9.315514 13.098986
H 9.412680 9.296135 11.405252
H 5.932873 7.147620 12.672459
H 6.672066 5.612146 13.150508
H 6.937603 7.075539 14.129511
H 12.129616 6.391256 19.330665
H 13.752270 6.586341 21.190055
H 16.100227 5.832337 20.878866
H 16.805997 4.871900 18.697932
H 15.174937 4.657536 16.849555
H 12.617305 10.240507 17.395614
H 11.147865 9.247508 17.433521
H 12.522389 8.859265 18.497619
H 13.000751 9.857946 14.352845
H 12.977466 8.233657 13.647218
H 11.481003 8.950704 14.295817
H 15.224136 9.110579 16.095912
H 15.178377 7.826643 17.313660
H 15.316922 7.418769 15.595180

H 5.472690 4.717851 15.406884
H 3.331979 3.578264 15.910044
H 3.336090 1.262835 16.813591
H 5.494367 0.098150 17.211073
H 7.632404 1.242038 16.709105
H 6.122043 7.003235 18.801239
H 6.711401 7.349037 17.165370
H 5.472989 6.095589 17.429610
H 6.953589 4.291496 20.208095
H 6.164497 3.482312 18.846238
H 7.827031 3.062677 19.285371
H 8.824822 6.552576 19.944521
H 9.977957 5.408972 19.241765
H 9.611199 6.943593 18.409094
H 9.018335 3.754823 11.467683
H 10.344113 2.733158 12.046019
H 9.070750 2.037376 11.022527
H 13.777567 3.604984 14.815927
H 15.267694 3.792899 13.861608
H 14.479601 5.211638 14.579839
C 11.593662 7.565143 10.564358
C 11.933044 7.952754 9.269843
C 11.892137 7.026801 8.227597
C 11.514373 5.709753 8.486766
C 11.184319 5.316889 9.782466
H 11.631124 8.284661 11.377104
H 12.232249 8.978969 9.075149
H 12.153541 7.330554 7.217751
H 11.473818 4.985621 7.677619
H 10.881778 4.293703 9.981677
H 15.353748 5.233873 11.387101
H 13.964050 5.699833 10.392852
H 14.383556 6.640685 11.833269
H 14.178791 2.395208 11.488467
H 12.650693 2.057324 12.310006
H 12.650990 2.833576 10.715125
C 11.133780 1.471427 16.250494
H 11.055239 0.491755 16.734215
H 10.129374 1.832469 16.019174
H 11.665125 1.335120 15.302674
H 11.321901 1.707844 19.527832
H 11.794264 3.399056 19.746335
H 10.222117 3.002558 19.027120
H 13.742319 0.939870 17.790312
H 14.389146 2.066549 16.594568
H 14.397463 2.497640 18.315051

H 6.310633 1.812841 11.893056
H 5.739486 2.000976 13.558308
H 6.111728 3.428556 12.578330
H 8.429993 -0.114338 12.906249
H 9.666972 0.371961 14.075860
H 7.988000 0.144428 14.596337
UNCOL3xyz

124
scf done: -2085.086121
C 7.907000 -2.762264 21.505725
C 10.765102 -2.772192 24.050516
H 10.395426 -2.599681 23.036739
H 11.262704 -3.747826 24.067633
H 11.513377 -2.008938 24.285989
C 10.143651 -3.054929 27.019800
H 10.814763 -2.250093 27.333687
H 10.741112 -3.971883 26.945029
H 9.397153 -3.203063 27.803255
C 8.123045 -4.115665 24.982938
H 7.311854 -4.101859 25.717218
H 8.623747 -5.087610 25.059124
H 7.687967 -4.040977 23.983593
C 7.818786 -0.444997 25.985518
C 7.486951 -1.003687 27.340479
C 8.285719 -0.692610 28.446044
H 9.146600 -0.042581 28.319657
C 7.981746 -1.212026 29.702151
H 8.608978 -0.963943 30.554007
C 6.876762 -2.046989 29.867037
H 6.640343 -2.451572 30.847187
C 6.077788 -2.362354 28.768568
H 5.216720 -3.013934 28.889533
C 6.381331 -1.844709 27.509801
H 5.761489 -2.094646 26.653151
C 6.433327 2.016656 28.269394
H 6.172283 1.133322 28.855741
H 5.793392 2.844792 28.598241
H 7.468088 2.289378 28.497287
C 4.399006 1.137158 26.136053
H 4.196197 1.025533 25.067309
H 3.664615 1.834743 26.554110
H 4.251143 0.166648 26.619199
C 6.310606 3.492467 25.620635
H 7.250735 3.970002 25.912436
H 5.488693 4.134479 25.956887

H 6.285583 3.428396 24.530268
C 4.700507 -1.895095 23.426166
H 5.711678 -2.309143 23.447927
H 4.004791 -2.718243 23.230066
H 4.465408 -1.486458 24.414091
C 2.776196 0.164187 22.278235
H 2.647523 0.715592 23.214227
H 2.066890 -0.672636 22.289541
H 2.503960 0.823464 21.451137
C 4.646017 -1.330734 20.374968
H 4.528146 -0.572263 19.595118
H 3.845287 -2.067870 20.244502
H 5.598199 -1.843223 20.219386
C 5.983208 1.867674 21.991701
C 4.903074 2.527924 21.180639
C 3.897981 3.270653 21.809609
H 3.907275 3.376867 22.890475
C 2.889283 3.868316 21.057315
H 2.112664 4.441286 21.556519
C 2.875009 3.731994 19.669294
H 2.088201 4.198879 19.083428
C 3.873501 2.992573 19.036554
H 3.867520 2.880990 17.955739
C 4.882292 2.390985 19.787885
H 5.655599 1.808766 19.294472
C 6.313969 5.497908 21.974785
H 5.527889 5.433383 21.219859
H 6.818447 6.465012 21.857988
H 5.845046 5.489811 22.963383
C 8.239932 4.109672 20.017065
H 9.034499 3.368164 19.898022
H 8.642243 5.090053 19.737360
H 7.433630 3.867767 19.318452
C 9.055239 4.634039 22.936703
H 8.694368 4.832544 23.950297
H 9.524293 5.550841 22.562205
H 9.813736 3.849833 22.995448
C 8.501161 0.155668 19.084555
H 7.805943 -0.450605 19.670987
H 8.538607 -0.259880 18.071712
H 8.106549 1.174263 19.014811
C 11.294663 1.332423 18.789857
H 10.963783 2.373552 18.849798
H 11.197975 1.015973 17.744026
H 12.352999 1.293231 19.056841
C 11.002486 -1.549848 19.778388

H 12.007078 -1.548540 20.212078
H 11.091938 -1.870744 18.734219
H 10.397537 -2.291695 20.304882
C 10.880820 1.185995 22.420488
C 12.318633 1.386762 22.029851
C 12.745856 2.610431 21.503062
H 12.032637 3.420484 21.382084
C 14.077511 2.792080 21.136582
H 14.398666 3.746795 20.729111
C 14.995546 1.753489 21.291320
H 16.033749 1.896050 21.004826
C 14.575519 0.530988 21.813795
H 15.284872 -0.283152 21.934702
C 13.242879 0.346738 22.180756
H 12.915048 -0.608102 22.582348
C 12.684550 3.332050 24.717929
H 13.550585 2.969283 24.160705
H 13.036818 3.712960 25.684587
H 12.250316 4.174553 24.171227
C 12.279704 0.507496 25.865811
H 11.559216 -0.272532 26.126256
H 12.789935 0.821380 26.783504
H 13.030694 0.075035 25.197949
C 10.173506 2.704912 26.305347
H 9.785343 3.663201 25.947529
H 10.669845 2.881043 27.266252
H 9.325026 2.034521 26.463658
N 8.610294 -1.117337 25.143872
N 7.335853 0.728336 25.575450
N 5.846705 0.601583 22.400002
N 7.107116 2.505781 22.320090
N 9.992353 0.720012 21.536807
N 10.445424 1.449393 23.652858
N 8.003115 -1.711953 22.088331
O 7.815274 -3.798901 20.933034
Si 9.366792 -2.731558 25.320812
Si 6.156547 1.786160 26.408901
Si 4.518166 -0.562124 22.098743
Si 7.626661 4.140726 21.807804
Si 10.229736 0.174291 19.847518
Si 11.401067 1.982584 25.068846
U 8.182441 0.335957 23.182931
UNCSL3xyz

124
scf done: -2020.08230044

U 12.536699 6.582667 5.924189
N 11.954128 8.004156 4.014655
C 10.778504 8.416439 4.510505
C 10.052391 9.573522 3.886681
C 10.591410 10.863486 3.973490
C 9.921283 11.944807 3.404969
C 8.717758 11.747281 2.728209
C 8.185485 10.462850 2.623704
C 8.845980 9.381710 3.204409
N 12.684000 7.645381 8.108397
Si 13.127393 9.292412 8.696206
C 14.945246 9.453251 9.176745
S 15.247285 7.488057 5.393485
C 15.600375 9.157297 5.527680
N 15.897553 10.287957 5.587766
N 11.958558 5.472920 8.186625
Si 11.567115 3.876593 8.916513
N 13.859936 4.545296 5.434025
C 12.910211 3.972571 4.682349
C 13.229278 2.775030 3.832168
C 12.849347 1.483131 4.210107
C 13.180045 0.390121 3.411811
C 13.881624 0.575596 2.221309
C 14.256877 1.861764 1.834694
C 13.939412 2.955256 2.638691
N 11.701157 4.534409 4.688878
Si 10.188558 4.094373 3.837730
N 10.306206 7.779639 5.587326
Si 8.835587 8.208515 6.530211
C 9.005298 7.324513 8.185551
Si 12.688637 8.417824 2.417827
C 13.765906 9.966143 2.477517
C 12.410544 6.546366 8.831776
C 12.649842 6.540612 10.312561
C 12.049699 9.844039 10.153562
C 12.760310 10.436154 7.243522
Si 15.498062 3.894105 5.829226
C 16.776481 4.320623 4.505571
C 15.546031 2.014773 6.083008
C 15.966168 4.665191 7.491651
C 9.801373 5.426203 2.559432
C 10.125433 2.445771 2.909430
C 8.830477 4.040158 5.152711
C 11.404019 8.662232 1.044961
C 13.724135 6.909907 1.942861
C 7.238604 7.604744 5.701909

C 8.630401 10.057331 6.891416
H 13.408939 10.231384 6.387229
H 12.953512 11.473663 7.539036
H 11.714138 10.362449 6.935839
H 15.180497 8.913946 10.097515
H 15.159851 10.515371 9.344821
H 15.607746 9.110842 8.377759
H 10.983860 9.705113 9.947144
H 12.216716 10.913571 10.326348
H 12.291655 9.315378 11.078198
H 16.106225 5.746170 7.414047
H 16.904187 4.224353 7.847312
H 15.195892 4.462856 8.243151
H 16.561951 3.793571 3.571024
H 17.770127 4.005626 4.845317
H 16.808337 5.394837 4.308779
H 14.783369 1.656282 6.781033
H 16.524475 1.777019 6.518318
H 15.448578 1.454568 5.150716
H 12.307284 1.332889 5.138569
H 12.887550 -0.609633 3.721869
H 14.135615 -0.277849 1.598495
H 14.802176 2.016693 0.907530
H 14.239252 3.955574 2.339286
H 14.177846 7.070962 0.958651
H 13.091328 6.018245 1.878071
H 13.150518 10.869592 2.529503
H 14.355848 10.022914 1.555081
H 14.460162 9.974091 3.320999
H 10.664475 7.857620 1.008048
H 11.943030 8.663063 0.089659
H 10.874291 9.613889 1.129269
H 8.437933 8.380723 3.105995
H 7.255943 10.300005 2.084753
H 8.199497 12.590459 2.279637
H 10.342018 12.943073 3.490038
H 11.528403 11.018026 4.499867
H 8.082731 7.438075 8.764952
H 9.828854 7.755841 8.761519
H 9.206541 6.257691 8.064352
H 8.929027 3.154742 5.788581
H 7.844461 3.994732 4.676786
H 8.858798 4.923910 5.794782
H 10.517368 5.387644 1.732652
H 9.844462 6.423418 2.998939
H 8.799513 5.268696 2.143889

C 11.579735 6.582626 11.213086
C 11.814040 6.558365 12.586816
C 13.118446 6.483449 13.073190
C 14.188703 6.443210 12.180392
C 13.957799 6.477072 10.806399
H 10.565057 6.654368 10.835208
H 10.976695 6.600921 13.277927
H 13.300092 6.459218 14.143967
H 15.207380 6.381506 12.553098
H 14.791548 6.437311 10.112866
C 9.793585 3.835632 9.583176
C 11.669353 2.592808 7.542986
C 12.745180 3.281948 10.277990
H 11.378455 1.612688 7.936515
H 11.012112 2.845716 6.709584
H 12.691161 2.526077 7.162938
H 12.427688 2.272386 10.567128
H 13.776062 3.210021 9.917627
H 12.736926 3.903601 11.175156
H 9.578922 2.820909 9.938758
H 9.632241 4.518159 10.420732
H 9.065375 4.069741 8.801051
H 7.782161 10.173902 7.576693
H 9.509450 10.482212 7.384170
H 8.422057 10.644531 5.994442
H 6.435684 7.625712 6.448470
H 6.936141 8.253813 4.876928
H 7.317681 6.580789 5.328114
H 14.529757 6.727551 2.659266
H 9.133370 2.392454 2.443861
H 10.874331 2.370853 2.116880
H 10.234523 1.577669 3.562894
U 12.436895 6.545468 5.950381 2.323738
N 11.751123 7.991697 4.089385 -0.740395
C 10.600368 8.414342 4.628365 0.411160
C 9.822644 9.524591 3.981166 -0.019736
C 10.299891 10.839931 4.021965 -0.121233
C 9.579148 11.869058 3.420041 -0.129827
C 8.382120 11.593429 2.759180 -0.116089
C 7.905486 10.284094 2.708223 -0.129957
C 8.618589 9.255205 3.320760 -0.118445
N 12.749612 7.547983 8.160159 -0.741586
Si 13.352810 9.144249 8.712438 0.563874
C 15.208433 9.131126 9.074929 -0.529102
N 14.518195 7.644489 5.556717 -0.587557
C 15.562291 8.199143 5.366251 -0.052280

S 16.986783 8.958550 5.107154 -0.093252
N 11.875885 5.436341 8.208803 -0.697378
Si 11.417910 3.841507 8.888579 0.556455
N 13.824229 4.611263 5.365932 -0.736819
C 12.916159 4.000808 4.597033 0.418102
C 13.327025 2.858460 3.711585 -0.021366
C 12.952043 1.544100 4.010767 -0.120864
C 13.378541 0.489441 3.205038 -0.130659
C 14.171698 0.737863 2.085610 -0.113803
C 14.547230 2.045861 1.780720 -0.133348
C 14.137440 3.100069 2.594813 -0.119826
N 11.666934 4.470061 4.633369 -0.703644
Si 10.289987 3.984616 3.595908 0.559764
N 10.188822 7.824408 5.751086 -0.689286
Si 8.792916 8.328213 6.760003 0.562394
C 9.078978 7.592025 8.474462 -0.536433
Si 12.524710 8.475259 2.545112 0.561078
C 13.537329 10.063312 2.709848 -0.527698
C 12.398148 6.469920 8.871475 0.420638
C 12.614561 6.436364 10.356486 -0.022880
C 12.433519 9.792800 10.236603 -0.532918
C 13.000918 10.333024 7.284515 -0.550202
Si 15.505306 4.134277 5.769056 0.566320
C 16.744328 4.726911 4.467990 -0.532572
C 15.712973 2.266860 6.012752 -0.532539
C 15.867008 4.959454 7.431138 -0.549203
C 10.614702 4.351079 1.767858 -0.541155
C 9.755324 2.169680 3.754000 -0.533821
C 8.822274 4.994057 4.209723 -0.524295
C 11.275892 8.670312 1.131626 -0.533809
C 13.692297 7.056076 2.105554 -0.546916
C 7.137603 7.660426 6.120233 -0.527152
C 8.626758 10.209706 6.957610 -0.535996
H 13.367411 9.943200 6.330877 0.159124
H 13.505050 11.288448 7.465424 0.140324
H 11.928089 10.529852 7.196857 0.137265
H 15.432939 8.613368 10.011613 0.130878
H 15.564398 10.163330 9.169339 0.140754
H 15.776610 8.660566 8.268114 0.162567
H 11.348729 9.779371 10.093204 0.133185
H 12.731970 10.835141 10.400556 0.136154
H 12.667879 9.229507 11.142726 0.149734
H 15.792038 6.048334 7.367608 0.162317
H 16.883622 4.712362 7.755905 0.137243
H 15.173000 4.602581 8.199146 0.138171
H 16.830431 4.010843 3.645263 0.133818

H 17.734502 4.841616 4.922176 0.145709
H 16.460693 5.698821 4.056331 0.159389
H 15.056538 1.883176 6.800144 0.131181
H 16.747404 2.077397 6.323943 0.136357
H 15.527682 1.695400 5.100883 0.149827
H 12.343950 1.348301 4.888089 0.139847
H 13.092500 -0.528459 3.455430 0.129085
H 14.499606 -0.084463 1.455899 0.127704
H 15.165930 2.247142 0.910548 0.131309
H 14.442147 4.116535 2.365001 0.145011
H 14.241407 7.299431 1.189468 0.137809
H 13.136535 6.130503 1.928600 0.136742
H 12.901725 10.947588 2.805759 0.129261
H 14.152656 10.192540 1.812000 0.139898
H 14.213914 10.020954 3.567662 0.164777
H 10.509087 7.890200 1.150662 0.133769
H 11.809091 8.590137 0.177229 0.134815
H 10.771471 9.639750 1.156753 0.150087
H 8.250378 8.234849 3.271831 0.135992
H 6.978007 10.061418 2.187870 0.129054
H 7.823622 12.395824 2.285398 0.128074
H 9.952825 12.888096 3.468737 0.131424
H 11.227839 11.055786 4.542886 0.145461
H 8.132215 7.548442 9.023537 0.128298
H 9.777505 8.210804 9.044866 0.141188
H 9.492476 6.582786 8.415263 0.153336
H 8.591634 4.730280 5.247157 0.138023
H 7.935339 4.778311 3.604083 0.128349
H 9.017219 6.067502 4.179152 0.152849
H 11.461227 3.772476 1.386811 0.144984
H 10.822043 5.412198 1.604420 0.147109
H 9.732678 4.084965 1.174313 0.134404
C 11.530201 6.532209 11.235806 -0.117310
C 11.738003 6.503761 12.613643 -0.131227
C 13.028312 6.369855 13.125689 -0.116164
C 14.112362 6.273238 12.253369 -0.130192
C 13.908525 6.313275 10.875564 -0.119019
H 10.527318 6.648781 10.837195 0.136229
H 10.890229 6.588568 13.287976 0.128808
H 13.188648 6.342632 14.199832 0.127933
H 15.119730 6.164920 12.645736 0.131231
H 14.751697 6.232213 10.196101 0.145268
C 9.631603 3.864676 9.524959 -0.525337
C 11.502800 2.590517 7.480596 -0.530436
C 12.554190 3.188150 10.257962 -0.530180
H 11.055601 1.643380 7.802130 0.127324

H 10.972590 2.949019 6.595991 0.153606
H 12.541877 2.399886 7.196810 0.141620
H 12.260999 2.152264 10.467922 0.132733
H 13.600658 3.174508 9.937505 0.135634
H 12.489315 3.753118 11.189882 0.150154
H 9.358872 2.858993 9.865379 0.134292
H 9.493643 4.547740 10.367173 0.132201
H 8.929011 4.149555 8.735505 0.136593
H 8.136866 10.418029 7.915974 0.133297
H 9.596772 10.713790 6.965597 0.137752
H 8.022714 10.656904 6.163773 0.148887
H 6.365805 7.872774 6.869601 0.133486
H 6.831028 8.139603 5.186956 0.131491
H 7.162186 6.579455 5.960928 0.138261
H 14.425681 6.883800 2.898276 0.159949
H 8.772069 2.072919 3.277880 0.134386
H 10.435575 1.474392 3.258823 0.143700
H 9.644786 1.864436 4.799221 0.132591
UOCNL3xyz

124
scf done: -2085.061625
C 13.231644 0.352351 22.146347
C 12.304123 1.393775 22.028922
C 12.721686 2.628513 21.520727
C 14.047059 2.818834 21.136675
C 14.968681 1.779015 21.258235
C 14.558700 0.546422 21.764713
C 10.870723 1.181253 22.427061
N 10.428338 1.476241 23.651342
U 8.183720 0.351381 23.200634
O 8.008724 -1.667951 22.165900
C 7.906450 -2.724378 21.494347
N 7.812486 -3.715260 20.869446
N 9.990160 0.675434 21.557279
Si 10.227734 0.116882 19.867207
C 10.981569 -1.613594 19.806074
Si 11.390981 2.008576 25.065916
C 10.166030 2.711320 26.315989
C 8.495911 0.100671 19.111216
C 11.294759 1.275374 18.812912
C 12.661069 3.371281 24.718636
C 12.285294 0.529988 25.839193
N 8.592017 -1.112223 25.139023
Si 9.337734 -2.737729 25.297009
C 8.088892 -4.108215 24.931738

C 7.811432 -0.435858 25.988001
N 7.334965 0.742562 25.581790
Si 6.157456 1.799819 26.422381
C 6.311502 3.508088 25.638105
C 7.487706 -0.994168 27.344451
C 8.295312 -0.682460 28.443505
C 8.002528 -1.203078 29.701665
C 6.900008 -2.039542 29.875042
C 6.092600 -2.355565 28.783048
C 6.385120 -1.837188 27.521991
C 10.734552 -2.770909 24.024405
C 10.108948 -3.074500 26.995409
C 6.434713 2.021315 28.283401
C 4.400537 1.149805 26.146634
N 5.879654 0.594455 22.371790
Si 4.551178 -0.576290 22.071137
C 4.690515 -1.367243 20.360754
C 6.003627 1.870450 21.989844
N 7.116834 2.515701 22.343979
Si 7.633351 4.154106 21.831139
C 9.051696 4.656841 22.968509
C 4.921281 2.531335 21.183465
C 3.923128 3.279162 21.817357
C 2.907643 3.872378 21.070894
C 2.881174 3.728344 19.683829
C 3.873498 2.984859 19.046234
C 4.887786 2.385581 19.791906
C 4.739927 -1.896002 23.410760
C 2.812620 0.157905 22.246735
C 6.317728 5.510224 21.980670
C 8.258205 4.109975 20.044546
H 10.363261 -2.654126 23.003281
H 11.260907 -3.730002 24.076697
H 11.460732 -1.978233 24.229808
H 10.780155 -2.272909 27.317076
H 10.703984 -3.992392 26.916277
H 9.358918 -3.226010 27.774887
H 7.229741 -4.054507 25.606968
H 8.568470 -5.082318 25.081829
H 7.728542 -4.075003 23.900059
H 9.154727 -0.031664 28.310491
H 8.636958 -0.955233 30.548311
H 6.671855 -2.444774 30.856953
H 5.233934 -3.009121 28.910438
H 5.758969 -2.089413 26.670593
H 6.169948 1.136023 28.865160

H 5.797342 2.849994 28.615456
H 7.470255 2.288674 28.513218
H 4.192970 1.042885 25.078319
H 3.667064 1.844907 26.570400
H 4.254008 0.177176 26.625846
H 7.252051 3.985052 25.929273
H 5.490139 4.149206 25.976863
H 6.284400 3.445854 24.547712
H 5.724023 -2.370449 23.384960
H 3.991597 -2.682242 23.263971
H 4.582361 -1.463219 24.403738
H 2.684271 0.714577 23.179586
H 2.101248 -0.676886 22.260322
H 2.544472 0.813879 21.415697
H 4.656494 -0.615858 19.566361
H 3.841031 -2.044157 20.212196
H 5.603104 -1.959358 20.248663
H 3.942172 3.391662 22.897515
H 2.135359 4.447996 21.573873
H 2.089080 4.192020 19.102424
H 3.857962 2.867223 17.966146
H 5.654939 1.798637 19.294530
H 5.538295 5.441559 21.219378
H 6.824016 6.476125 21.862624
H 5.840387 5.508008 22.965178
H 9.058161 3.372615 19.935051
H 8.654968 5.090327 19.757476
H 7.457076 3.856007 19.344263
H 8.683315 4.857332 23.978957
H 9.518270 5.574557 22.593395
H 9.812949 3.876097 23.033425
H 7.823788 -0.588518 19.628812
H 8.548819 -0.217661 18.064567
H 8.060511 1.104643 19.135479
H 10.962898 2.316304 18.869470
H 11.203228 0.955817 17.767789
H 12.351826 1.237291 19.085134
H 11.960993 -1.642266 20.292540
H 11.122667 -1.906417 18.759263
H 10.332632 -2.361585 20.269729
H 12.005677 3.439698 21.425935
H 14.360382 3.781180 20.741146
H 16.002186 1.928470 20.958329
H 15.271180 -0.268379 21.860436
H 12.911244 -0.610955 22.533521
H 13.526873 3.019182 24.154524

H 13.014971 3.748038 25.686161
H 12.217252 4.213879 24.179973
H 11.572519 -0.255403 26.105462
H 12.808351 0.839477 26.751048
H 13.027739 0.105228 25.157072
H 9.763691 3.666299 25.965046
H 10.670354 2.890572 27.271970
H 9.326430 2.031784 26.481340
UOCPL3xyz

124
scf done: -2036.858239
U 10.254910 5.492380 15.052788
N 8.439766 4.716470 16.509301
Si 7.749390 5.333749 18.046468
C 7.120338 3.953943 19.182834
N 11.707913 5.086785 12.965441
Si 13.321017 4.467110 12.497398
C 14.321568 4.243921 14.078241
N 9.749237 6.249286 12.787882
Si 8.366632 7.140913 12.075829
C 8.662111 9.002852 11.996497
N 12.201410 6.481343 16.166302
C 12.541554 5.389743 16.859650
C 13.545015 5.479820 17.977118
C 13.161703 5.999984 19.219712
C 14.087700 6.104919 20.256319
C 15.409071 5.706585 20.058670
C 15.798966 5.197257 18.820774
C 14.871984 5.078620 17.786721
N 11.934211 4.246862 16.534632
Si 12.073174 2.659667 17.350006
C 11.249309 2.697431 19.052655
N 8.717212 3.447519 14.626043
Si 8.260477 2.295924 13.324840
C 8.605418 0.489244 13.781018
O 9.338519 7.535618 15.590708
C 8.870290 8.661830 15.841250
P 8.261156 10.105878 16.176723
C 7.987780 3.749555 15.701642
C 6.692793 3.053492 16.007262
C 5.471939 3.701062 15.784816
C 4.271324 3.058320 16.078590
C 4.278228 1.767505 16.607114
C 5.491687 1.120094 16.836881
C 6.693789 1.757747 16.535109

C 9.186644 6.199302 18.914608
C 6.367217 6.589567 17.769293
C 10.892337 5.838286 12.223793
C 11.232243 6.242270 10.816665
C 6.913560 6.785434 13.230696
C 7.902070 6.508606 10.349927
C 13.172444 2.771666 11.664450
C 14.342264 5.613514 11.386197
Si 12.891168 8.137460 16.198320
C 12.401224 8.884687 14.533032
C 14.782508 8.144836 16.301391
C 12.189178 9.177973 17.607292
C 13.835554 1.989901 17.553876
C 6.451655 2.423673 12.771082
C 9.324634 2.720129 11.830229
H 6.947093 6.964801 10.062973
H 8.640288 6.773220 9.590263
H 7.766023 5.423655 10.333707
H 7.738608 9.496271 11.675045
H 8.927082 9.409231 12.976659
H 9.446346 9.262998 11.280863
H 6.004950 7.250904 12.834787
H 6.733550 5.708786 13.304538
H 7.079509 7.186382 14.233861
H 12.136840 6.323562 19.371912
H 13.776178 6.503707 21.217155
H 16.131462 5.794910 20.864522
H 16.827905 4.890815 18.657196
H 15.179485 4.685842 16.823550
H 12.583294 10.197803 17.536406
H 11.098999 9.243240 17.551698
H 12.473304 8.778218 18.584328
H 12.812240 9.894705 14.435885
H 12.796126 8.279911 13.710409
H 11.315802 8.961027 14.427365
H 15.133838 9.167544 16.123094
H 15.149423 7.828561 17.279926
H 15.236064 7.502360 15.540963
H 5.465201 4.702282 15.365677
H 3.329700 3.566367 15.893011
H 3.342223 1.268883 16.839989
H 5.503731 0.118149 17.255614
H 7.637561 1.257016 16.726750
H 6.087488 7.027780 18.733916
H 6.693063 7.407086 17.120321
H 5.471608 6.132215 17.342119

H 6.943174 4.377817 20.177874
H 6.181598 3.520618 18.830548
H 7.848163 3.144997 19.291637
H 8.838064 6.639859 19.854501
H 9.983371 5.489050 19.151774
H 9.599204 7.009074 18.306480
H 9.092952 3.725657 11.471958
H 10.390633 2.674943 12.064271
H 9.116521 2.010798 11.022482
H 13.794282 3.620643 14.801750
H 15.283611 3.776548 13.840690
H 14.516794 5.214351 14.542753
C 11.600252 7.563247 10.534783
C 11.923872 7.941938 9.233829
C 11.871983 7.008311 8.199129
C 11.499814 5.692777 8.472499
C 11.185859 5.308754 9.774787
H 11.645522 8.289258 11.341055
H 12.218286 8.967332 9.028149
H 12.119494 7.305273 7.183806
H 11.451912 4.962244 7.669525
H 10.891724 4.286002 9.985415
H 15.361753 5.211428 11.342115
H 13.961002 5.684642 10.366048
H 14.406344 6.624779 11.799528
H 14.177079 2.378890 11.468868
H 12.656190 2.053413 12.309114
H 12.642935 2.819510 10.709136
C 11.168981 1.449137 16.228072
H 11.080526 0.470729 16.712754
H 10.169240 1.810615 15.977559
H 11.718824 1.309597 15.291251
H 11.266814 1.699085 19.503976
H 11.767714 3.381387 19.731391
H 10.206014 3.015497 18.974925
H 13.755390 0.929137 17.821403
H 14.413579 2.050363 16.626634
H 14.395006 2.494260 18.343999
H 6.337496 1.837938 11.851037
H 5.749971 2.030928 13.509789
H 6.165825 3.454806 12.541667
H 8.425182 -0.135094 12.898106
H 9.640594 0.336299 14.095588
H 7.946546 0.132375 14.576755
UPCOL3xyz

124
scf done: -2036.848408
U 10.229426 5.595276 15.107618
N 8.407323 4.779930 16.532440
Si 7.733010 5.221036 18.142267
C 7.248094 3.709258 19.180461
N 11.669053 5.149269 13.024294
Si 13.284048 4.524006 12.565338
C 14.239904 4.192646 14.154408
N 9.691145 6.282033 12.823700
Si 8.294648 7.096057 12.040010
C 8.498655 8.972952 11.990240
N 12.288602 6.510877 16.122873
C 12.546229 5.439412 16.879800
C 13.533347 5.532405 18.014034
C 13.157456 6.165220 19.206261
C 14.057453 6.264898 20.265960
C 15.346502 5.747318 20.142377
C 15.730359 5.125603 18.955121
C 14.827903 5.013411 17.897893
N 11.870207 4.319554 16.614309
Si 11.999220 2.748939 17.475010
C 11.391469 2.839691 19.262561
N 8.712387 3.534824 14.633453
Si 8.279598 2.379285 13.326408
C 8.721902 0.588137 13.758692
P 9.493872 8.309039 16.049685
C 7.938626 8.731723 15.599158
O 6.864179 9.124352 15.310457
C 7.973159 3.817152 15.704404
C 6.666226 3.121162 15.958970
C 5.457261 3.795617 15.752744
C 4.243854 3.144072 15.966281
C 4.225879 1.818237 16.399225
C 5.427683 1.144182 16.616059
C 6.642350 1.790496 16.393052
C 9.104654 6.115648 19.082537
C 6.218236 6.344817 18.033762
C 10.850585 5.890160 12.276707
C 11.207327 6.320072 10.881576
C 6.801892 6.567103 13.070974
C 7.947620 6.507245 10.271068
C 13.157192 2.872892 11.643995
C 14.351546 5.695388 11.524365
Si 13.215043 8.050025 16.018932
C 12.767491 8.784731 14.336291

C 15.093576 7.792279 16.011617
C 12.797089 9.258796 17.408329
C 13.738481 1.988774 17.480969
C 6.458294 2.389561 12.797711
C 9.291301 2.866302 11.819373
H 6.990312 6.950799 9.967791
H 8.705694 6.831875 9.556547
H 7.840534 5.421808 10.197415
H 7.542773 9.443708 11.739759
H 8.834031 9.368488 12.952370
H 9.225874 9.258091 11.222785
H 5.882234 6.978639 12.641768
H 6.723248 5.476272 13.053390
H 6.846546 6.897520 14.110816
H 12.157231 6.577100 19.301545
H 13.751209 6.751390 21.187567
H 16.048793 5.830550 20.966666
H 16.735007 4.726314 18.849029
H 15.131200 4.532426 16.973894
H 13.276422 10.223526 17.208477
H 11.718792 9.422444 17.477747
H 13.161997 8.894456 18.372842
H 13.344971 9.698898 14.160700
H 13.009357 8.075007 13.538588
H 11.705881 9.039384 14.275217
H 15.558763 8.757796 15.780226
H 15.480320 7.455808 16.975413
H 15.416817 7.084361 15.242604
H 5.469274 4.823653 15.404494
H 3.311490 3.673151 15.791602
H 3.279621 1.312691 16.568268
H 5.420226 0.114665 16.962475
H 7.576393 1.268715 16.575775
H 5.972653 6.700286 19.041375
H 6.386410 7.219727 17.402366
H 5.347542 5.803544 17.653544
H 7.031283 4.061479 20.195731
H 6.356100 3.203300 18.805217
H 8.053712 2.972437 19.252582
H 8.756801 6.324408 20.100267
H 10.003065 5.496691 19.156906
H 9.368414 7.066250 18.611658
H 8.949639 3.829096 11.432655
H 10.351193 2.956246 12.065060
H 9.173906 2.112727 11.034213
H 13.684449 3.537554 14.826104

H 15.199682 3.722279 13.912886
H 14.439739 5.128604 14.682342
C 11.549548 7.655047 10.632282
C 11.888083 8.064173 9.344927
C 11.871711 7.150142 8.291632
C 11.520498 5.822692 8.531951
C 11.193801 5.406190 9.821168
H 11.560847 8.366482 11.452617
H 12.164171 9.099206 9.163188
H 12.129841 7.472337 7.286660
H 11.499630 5.108360 7.713429
H 10.918466 4.373521 10.007517
H 15.356710 5.258759 11.467218
H 13.982033 5.823210 10.506204
H 14.450975 6.684245 11.981689
H 14.173692 2.507941 11.455111
H 12.638611 2.111061 12.234374
H 12.651888 2.964047 10.677845
C 10.924527 1.558529 16.491975
H 10.839904 0.595576 17.006888
H 9.923368 1.961769 16.329621
H 11.373657 1.377252 15.510564
H 11.465732 1.849340 19.725767
H 11.989306 3.534895 19.858298
H 10.346445 3.155571 19.312478
H 13.632978 0.923434 17.720272
H 14.222070 2.056302 16.501820
H 14.399260 2.435331 18.226482
H 6.369646 1.731778 11.924109
H 5.781754 2.014753 13.568394
H 6.116947 3.382999 12.493413
H 8.628780 -0.017521 12.849791
H 9.745959 0.489330 14.125404
H 8.045572 0.165407 14.506466
USCNL3xyz

124
scf done: -2020.051361
U 12.536699 6.582667 5.924189
N 11.954128 8.004156 4.014655
C 10.778504 8.416439 4.510505
C 10.052391 9.573522 3.886681
C 10.591410 10.863486 3.973490
C 9.921283 11.944807 3.404969
C 8.717758 11.747281 2.728209
C 8.185485 10.462850 2.623704

C 8.845980 9.381710 3.204409
N 12.684000 7.645381 8.108397
Si 13.127393 9.292412 8.696206
C 14.945246 9.453251 9.176745
S 15.247285 7.488057 5.393485
C 15.600375 9.157297 5.527680
N 15.897553 10.287957 5.587766
N 11.958558 5.472920 8.186625
Si 11.567115 3.876593 8.916513
N 13.859936 4.545296 5.434025
C 12.910211 3.972571 4.682349
C 13.229278 2.775030 3.832168
C 12.849347 1.483131 4.210107
C 13.180045 0.390121 3.411811
C 13.881624 0.575596 2.221309
C 14.256877 1.861764 1.834694
C 13.939412 2.955256 2.638691
N 11.701157 4.534409 4.688878
Si 10.188558 4.094373 3.837730
N 10.306206 7.779639 5.587326
Si 8.835587 8.208515 6.530211
C 9.005298 7.324513 8.185551
Si 12.688637 8.417824 2.417827
C 13.765906 9.966143 2.477517
C 12.410544 6.546366 8.831776
C 12.649842 6.540612 10.312561
C 12.049699 9.844039 10.153562
C 12.760310 10.436154 7.243522
Si 15.498062 3.894105 5.829226
C 16.776481 4.320623 4.505571
C 15.546031 2.014773 6.083008
C 15.966168 4.665191 7.491651
C 9.801373 5.426203 2.559432
C 10.125433 2.445771 2.909430
C 8.830477 4.040158 5.152711
C 11.404019 8.662232 1.044961
C 13.724135 6.909907 1.942861
C 7.238604 7.604744 5.701909
C 8.630401 10.057331 6.891416
H 13.408939 10.231384 6.387229
H 12.953512 11.473663 7.539036
H 11.714138 10.362449 6.935839
H 15.180497 8.913946 10.097515
H 15.159851 10.515371 9.344821
H 15.607746 9.110842 8.377759
H 10.983860 9.705113 9.947144

H 12.216716 10.913571 10.326348
H 12.291655 9.315378 11.078198
H 16.106225 5.746170 7.414047
H 16.904187 4.224353 7.847312
H 15.195892 4.462856 8.243151
H 16.561951 3.793571 3.571024
H 17.770127 4.005626 4.845317
H 16.808337 5.394837 4.308779
H 14.783369 1.656282 6.781033
H 16.524475 1.777019 6.518318
H 15.448578 1.454568 5.150716
H 12.307284 1.332889 5.138569
H 12.887550 -0.609633 3.721869
H 14.135615 -0.277849 1.598495
H 14.802176 2.016693 0.907530
H 14.239252 3.955574 2.339286
H 14.177846 7.070962 0.958651
H 13.091328 6.018245 1.878071
H 13.150518 10.869592 2.529503
H 14.355848 10.022914 1.555081
H 14.460162 9.974091 3.320999
H 10.664475 7.857620 1.008048
H 11.943030 8.663063 0.089659
H 10.874291 9.613889 1.129269
H 8.437933 8.380723 3.105995
H 7.255943 10.300005 2.084753
H 8.199497 12.590459 2.279637
H 10.342018 12.943073 3.490038
H 11.528403 11.018026 4.499867
H 8.082731 7.438075 8.764952
H 9.828854 7.755841 8.761519
H 9.206541 6.257691 8.064352
H 8.929027 3.154742 5.788581
H 7.844461 3.994732 4.676786
H 8.858798 4.923910 5.794782
H 10.517368 5.387644 1.732652
H 9.844462 6.423418 2.998939
H 8.799513 5.268696 2.143889
C 11.579735 6.582626 11.213086
C 11.814040 6.558365 12.586816
C 13.118446 6.483449 13.073190
C 14.188703 6.443210 12.180392
C 13.957799 6.477072 10.806399
H 10.565057 6.654368 10.835208
H 10.976695 6.600921 13.277927
H 13.300092 6.459218 14.143967

H 15.207380 6.381506 12.553098
H 14.791548 6.437311 10.112866
C 9.793585 3.835632 9.583176
C 11.669353 2.592808 7.542986
C 12.745180 3.281948 10.277990
H 11.378455 1.612688 7.936515
H 11.012112 2.845716 6.709584
H 12.691161 2.526077 7.162938
H 12.427688 2.272386 10.567128
H 13.776062 3.210021 9.917627
H 12.736926 3.903601 11.175156
H 9.578922 2.820909 9.938758
H 9.632241 4.518159 10.420732
H 9.065375 4.069741 8.801051
H 7.782161 10.173902 7.576693
H 9.509450 10.482212 7.384170
H 8.422057 10.644531 5.994442
H 6.435684 7.625712 6.448470
H 6.936141 8.253813 4.876928
H 7.317681 6.580789 5.328114
H 14.529757 6.727551 2.659266
H 9.133370 2.392454 2.443861
H 10.874331 2.370853 2.116880
H 10.234523 1.577669 3.562894

NBO Analysis of the O-bound Th(OCP)(amid)$_3$ complex **4** vs the P-bound isomer:

O – bound Th(OCP)(amid)$_3$

162. CR (1) O 25	/193. LP*(3)Th 1	12.11	19.23	0.455
227. LP (1) O 25	/193. LP*(3)Th 1	57.28	1.04	0.223
227. LP (1) O 25	/194. LP*(4)Th 1	18.51	0.91	0.118
227. LP (1) O 25	/194. LP*(4)Th 1	18.51	0.91	0.118

P – bound Th(OCP)(amid)$_3$

230. LP (1) P 27	/193. LP*(3)Th 1	0.18	0.71	0.011
230. LP (1) P 27	/194. LP*(4)Th 1	0.43	0.58	0.015
230. LP (1) P 27	/197. LP*(7)Th 1	0.09	0.61	0.007

230. LP (1) P 27	/200. LP*(10)Th 1	0.89	0.65	0.022
230. LP (1) P 27	/203. LP*(13)Th 1	3.63	0.60	0.042

E. References

[1] J. L. Kiplinger, D. E. Morris, B. L. Scott, C. J. Burns, *Organometallics* **2002**, *21*, 5978–5982.

[2] T. Cantat, B. L. Scott, J. L. Kiplinger, *Chem. Commun.* **2010**, *46*, 919–921.

[3] V. Volkis, E. Nelkenbaum, A. Lisovskii, G. Hasson, R. Semiat, M. Kapon, M. Botoshansky, Y. Eishen, M. S. Eisen, *J. Am. Chem. Soc.* **2003**, *125*, 2179–94.

[4] F. F. Puschmann, D. Stein, D. Heift, C. Hendriksen, Z. a Gal, H.-F. Grützmacher, H. Grützmacher, *Angew. Chem. Int. Ed. Engl.* **2011**, *50*, 8420–8423.

[5] M. Wedler, F. Knösel, M. Noltemeyer, F. T. Edelmann, U. Behrens, *J. Organomet. Chem.* **1990**, *388*, 21–45.

[6] SADABS: Bruker-Nonius Area Detector Scaling and Absorption, **2003**.

[7] G. M. Sheldrick, *Acta Crystallogr. Sect. A* **2008**, *64*, 112–122.

[8] Becke, A. D. Density-functional thermochemistry. III. The role of exact exchange. *J. Chem. Phys.* **1993**, *98*, 5648-5652.

[9] Perdew, J. P. & Wang, Y. Erratum: Pair-distribution function and its coupling-constant average for the spin-polarized electron gas [*Phys. Rev. B* **1992**, *46*, 12 947]. *Phys. Rev. B* **1997**, *56*, 7018-7018.

[10] Gaussian 03 (Pittsburgh PA **2006**).

[11] Hehre, W. J.; Ditchfield, R.; Pople, J. A. *J. Chem. Phys.* **1972**, *56*, 2257.

[12] (a) Kuechle, W.; Dolg, M.; Stoll, H.; Preuss,H. *J. Chem. Phys.* **1994**, *100*, 7535. (b) Cao, X.; Dolg, M.; Stoll, H. *J. Chem. Phys.* **2003**, *118*, 487. (c) Cao, X.; Dolg, M. *J. Molec. Struct.* (Theochem) **2004**, *673*, 203. (d) Bergner, A.; Dolg, M.; Kuechle, W.; Stoll, H.; Preuss, H. *Mol. Phys.* **1993**, 80, 1431.

[13] Reed, A. E., Curtiss, L. A. & Weinhold, F. Intermolecular interactions from a natural bond orbital, donor-acceptor viewpoint. *Chem. Rev.* **1988**, *88*, 899-926.
